# Green hydrothermal synthesis yields perylenebisimide–SiO_2_ hybrid materials with solution-like fluorescence and photoredox activity†

**DOI:** 10.1039/d1ta03214c

**Published:** 2022-06-13

**Authors:** Hipassia M. Moura, Herwig Peterlik, Miriam M. Unterlass

**Affiliations:** Universität Konstanz, Department of Chemistry, Solid State Chemistry Universitätsstrasse 10 D-78464 Konstanz Germany miriam.unterlass@uni-konstanz.de; CeMM – Research Center for Molecular Medicine of the Austrian Academy of Sciences Lazarettgasse 14, AKH BT 25.3 1090 Wien Austria; Universität Wien, Faculty of Physics Boltzmanngasse 5 1090 Wien Austria

## Abstract

In organic–inorganic hybrid materials' (HMs) synthesis, it is intrinsically challenging to, at the same time, achieve (i) the concomitant synthesis of the components, (ii) nanoscopic interpenetration of the components, and (iii) covalent linking of the components. We here report the one-pot hydrothermal synthesis (HTS) of inorganic–organic HMs consisting of perylene bisimide (PBI) dyes and silica, using nothing but water as the medium and directly from the corresponding bisanhydrides, *n*-alkyl amines, and alkoxysilane precursors. First, in the absence of a functionalized alkoxysilane for linking, a mixture of the products, PBI and SiO_2_, is obtained. This evinces that the two products can be synthesized in parallel in the same vessel. Except for minor micromorphological changes, the concomitant synthesis does not affect each component's physicochemical properties. The PBI/SiO_2_ mixtures do not show synergistic properties. Second, through adding the linker aminopropyltriethoxysilane (APTS), covalently-linked class II hybrids are obtained. These PBI@SiO_2_ class II hybrids show synergistic materials properties: increased thermal stability is obtained in combination with nanoscopic homogeneity. The PBI moieties are dissolved in the solid SiO_2_ matrix, while being covalently linked to the matrix. This leads to solution-like fluorescence with vibronic fine-structure of the dyes. Moreover, through tuning the SiO_2_ amount, the band gaps of the class II hybrid materials can be systematically shifted. We exploit these optoelectronic properties by using the PBI@SiO_2_ hybrids as heterogeneous and reusable photoredox catalysts for the reduction of aryl halides. Finally, we present a detailed small-angle X-ray scattering and powder X-ray diffraction study of PBI@SiO_2_ synthesized at various reaction times, revealing the existence of an ordered PBI-oligomeric silesquioxane-type intermediate, which subsequently further condenses to the final nanoscopically homogeneous PBI@SiO_2_ material. These ordered intermediates point at HTS′ propensity to favor crystallinity (to date known for organic and inorganic compounds, respectively) to also apply to hybrid structures, and shed additional light on the long-standing question of structure formation in the early stages of sol–gel processes: they corroborate Brown's hypothesis (1965) that trifunctional hydroxysilanes form surprisingly well controlled oligomers in the early stages of polycondensation.

## Abstract 2

PBI dyes@SiO_2_ hybrid materials are generated by hydrothermal synthesis. They exhibit solution-like fluorescence and photocatalytic activity. An in-depth materials formation study reveals a network of PBI-linked POSS-type cages.

## Graphical abstract

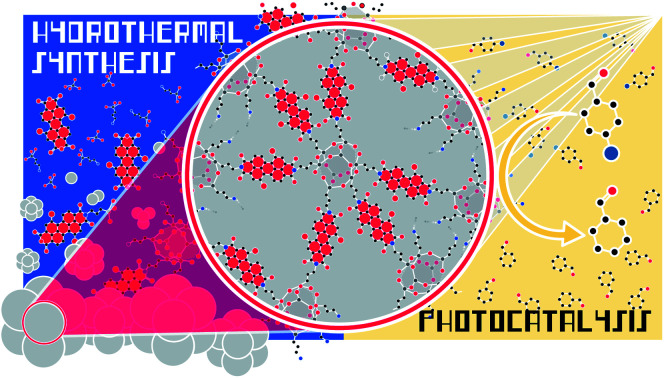

## 1. Introduction

A hybrid material (HM) is defined as an intricate combination of at least two fundamentally different materials that are chemically bonded to each other.^1^ Ideally, the components are connected on the nano- or even molecular level and the HM exhibits synergistic properties, *i.e.*, properties that exceed the mere sum of the individual components' features.^2^ Inorganic–organic HMs bear the most chemically different components, hence often uniting fundamentally different materials properties. One of the already early on heavily studied classes of hybrids were organic colorants, *i.e.*, dyes or pigments,‡‡Note that the term “dye” is used for colorants that are dissolved in a substrate, whereas “pigment” refers to finely dispersed color-generating substances. Note that in the following, we will for ease of readability use the term “dye”. embedded in metal oxides, M_*x*_O_*y*_, as the inorganic component.^3^ Such organic dye@M_*x*_O_*y*_ HMs combine the optoelectronic properties contributed by the dyes with, *e.g.*, high thermomechanical stabilities of the M_*x*_O_*y*_s. This improves the chemical, photochemical and thermal stability of the dyes,^4–10^ and generates promise for a wide range of applications, such as photovoltaic cells and artificial light harvesting,^11^ photocatalysis,^12^ fine chemical production,^13^ optical sensors,^14^ therapeutics carriers,^15^ or smart textiles.^16^

Synthetically speaking, the generation of dyes@M_*x*_O_*y*_, and of organic–inorganic HMs in general, bears one major challenge: the two components can hardly be synthesized and linked concomitantly, as they each require fundamentally different ways of preparation. The organic components' synthesis typically entails rather low reaction temperatures, apolar organic solvents, catalysts or promotors, and laborious purification, while the inorganic components' preparation rather involves high reaction temperatures in polar solvents (if solvents are used at all), and purification methodologies different from those of organics. Therefore, organic–inorganic hybrids are mainly prepared by (i) mixing the preformed components, (ii) the synthesis of the organic component around/within the inorganic component (or *vice versa*), or (iii) modification of the organic component to be both the organic constituent and precursor for synthesizing the inorganic component. Table S1 (ESI†) provides a comprehensive (52 entries) comparison of the to date reported syntheses of perylene bisimide dyes (PBIs)§§Note that PBIs are often also termed ‘perylene diimides’ and then abbreviated as PDIs./SiO_2_ hybrids, which are the most prototypical dye@M_*x*_O_*y*_ example and at the center of this contribution. None of these reported syntheses relies on simultaneous synthesis and linking of both constituents. Hence, non-homogeneous interpenetration of organic and inorganic components typically results, *i.e.*, the components rather self-aggregate. This in turn reduces the interfacial area between the inorganic and organic components compared to a molecular dispersion. As the synergistic effects in HMs are “born at the interface” and scale with the interfacial area, aggregation is disadvantageous with respect to synergistic materials properties. Specifically for dyes@M_*x*_O_*y*_, aggregation of the dyes may quench their optoelectronic properties,^17^ which are the main features for which they were typically chosen as HM component.

We hypothesized that truly concomitant synthesis of the dyes and the M_*x*_O_*y*_ components would allow for overcoming aggregation, and instead promote nanoscopic homogeneity. We have here set out to investigate both the concomitant synthesis of both individual components and class II HMs (components interacting through strong bonding). In particular, we chose perylene bisimides as the organic, and SiO_2_ as the inorganic component. First, PBIs@SiO_2_ are a prototypical example of dyes@M_*x*_O_*y*_, which allows for comparison with a large body of literature examples. Second, both components individually feature intriguing properties: PBIs show fluorescence quantum yields of 100% in solution,^18^ have been applied for organic-electronics, *e.g.*, for light-harvesting in solar cells,^19,20^ and their thermal stabilities *T*_D_ range with typically ≥400 °C at the upper end for organic dyes. SiO_2_ provides high thermal stability and is white and therefore contributes no color by itself. Third, and here most importantly, both components have been individually shown to be synthesizable by the same technique: hydrothermal synthesis (HTS).^21,22^ Therefore, our hypothesis for this work was that a simultaneous preparation (and linking) of both components would be feasible by HTS. Recently, we reported the to date only example of the concomitant synthesis of an organic–inorganic HM by HTS, specifically of polyimide–SiO_2_ HMs.^23^ In hindsight, this HTS was relatively straightforward, as the generated polyimide was semi aliphatic, based on the monomers hexamethylene diamine and pyromellitic acid, which are both well soluble in water, even at room temperature. Furthermore, the amount of SiO_2_ was quite low relative to the polyimide, and hence, the SiO_2_ formation is not expected to tremendously affect the polyimide formation.

The here targeted HTS of PBIs@SiO_2_ is significantly more challenging. First, PBIs themselves as well as their precursors are water insoluble at rt and still barley soluble in ‘hot water’. Second, their highly conjugated nature (which is the reason for their water insolubility) imparts strong intermolecular interactions (π–π stacking), which promotes self-aggregation and counteracts the intended molecular dispersion in the SiO_2_ matrix. Third, in the present work, we target high relative amounts of SiO_2_, to promote the PBI dispersion. For the high amounts of employed alkoxysilane precursors, the rapid generation of SiO_2_ gels (and hence increased viscosities) is expected during the HTS, which potentially makes PBI precursor diffusion and reaction more difficult. Fourth, the reaction times and temperatures required to generate PBIs and SiO_2_, respectively, by HTS are quite different: PBIs have been prepared at approx. 250 °C for several hours,^21^ while SiO_2_ is already generated at approx. 140 °C for 4 hours.^22^ Hence, it was not clear if common reaction parameters would be findable.

Before diving into the results of this study, allow us to briefly summarize HTS with respect to the to date rarely exploited synthesis of organic materials: in a nutshell, HTS is amenable for generating organic materials that are formed by condensations, especially cyclocondensations. All reported examples are fairly recent and comprise fully aromatic polyimides,^24^ perylene bisimide dyes,^21^ the fused heterocyclic compound perinone,^25^ quinoxalines,^26^ imidazole-linked polymers,^27^ polyamides,^28^ and polyazomethines.^29^ Superheated (*T* > 100 °C) liquid water is conventionally subdivided in three regimes: (i) the hydrothermal (100 °C < *T* ≤ 250 °C), (ii) the near-critical (250 °C ≤ *T* ≤ 374 °C), and (iii) the supercritical regime (*T* > 374 °C).^26^ In all three regimes, H_2_O's viscosity, density, and polarity are considerably decreased compared to liquid water at *T* < 100 °C. The hydrothermal regime stands out through featuring the highest ionic product (*K*_w_ = [H^+^][OH^−^]), and is hence especially well-suited as medium for reactions that profit from acid/base catalysis – such as the here employed condensations towards PBIs and SiO_2_. For the use of solely H_2_O as reaction medium, without the need for catalysts, the use of precursors that are exclusively built up of abundant elements (C, H, N, O, Si), quantitative product yields, H_2_O and EtOH as the only byproducts, and no necessity for product purification, the hydrothermal one-pot preparation of PBI@SiO_2_ HMs is considered a green synthetic approach.

## 2. Results and discussion

### 2.1 The simultaneous HTS of *n*-alkyl-PBIs and SiO_2_

**Fig. 1:**
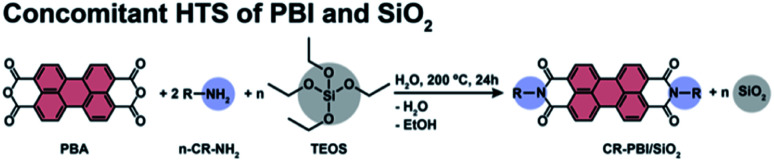
Synthesis of *n*-alkyl-PBI in the presence of TEOS (*n* = 1, 5, 10 and 100 equiv.). R = *n*-propylamine (C3), *n*-pentylamine (C5), *n*-octylamine (C8), *n*-tetradecylamine (C14).

We first aimed at a proof-of-concept that PBI dyes and SiO_2_ can be generated simultaneously by HTS. As PBI precursors we used perylene bisanhydride (PBA) and four different *n*-alkyl amines R–NH_2_ (*n*-propylamine, R = C_3_H_5_ (C3); *n*-pentylamine, R = C_5_H_9_ (C5); *n*-octylamine R = C_8_H_15_ (C8); and *n*-tetradecylamine, R = C_14_H_27_ (C14)) in molar ratio 1 : 2 (PBA : *n*-C_*n*_-NH_2_). Tetraethylorthosilicate (TEOS) was used as SiO_2_ precursor (Fig. 1). In this first set of experiments, we explored different amounts of TEOS, namely 1, 5, 10 and 100 equiv., with respect to PBA (and hence also the formed PBI). The precursors (*c*(PBA) = 0.03 mol L^−1^ = 1/2 *c*(R–NH_2_) = 1/*n c*(TEOS, *n* equiv.)) were supplied in 15 mL of distilled H_2_O and the dispersions were enclosed in an autoclave. The autoclave was then placed in an oven and heated to the reaction temperature (*T*_R_) of 200 °C and kept there for a reaction time (*t*_R_) of 24 h (see ESI†). After *t*_R_, the autoclave was rapidly cooled back to *r.t.* by quenching with cold tap water, and the products were isolated by filtration.

**Fig. 2:**
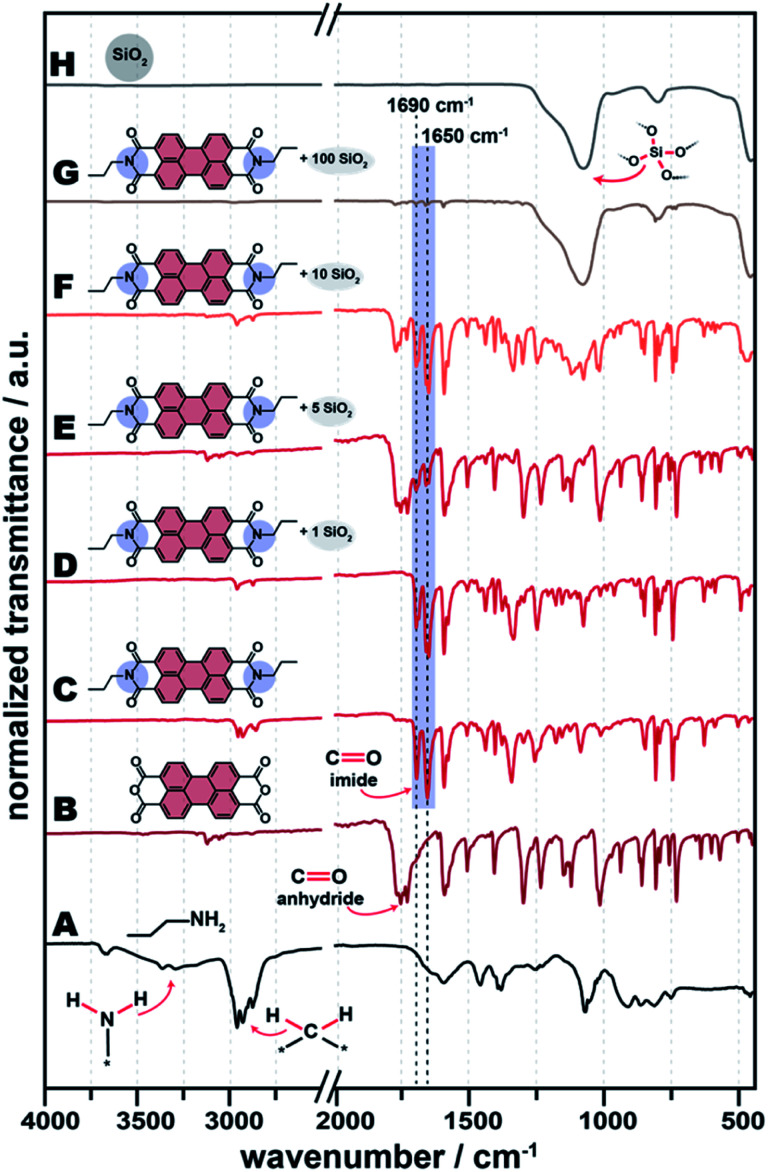
ATR-FTIR spectra of PBI-SiO_2_ synthesized without a linker. Shown are the spectra of starting compounds *n*-propylamine (A) and PBA (B), pure C3-PBI dye (C), C3-PBI/SiO_2_ (1 equiv. TEOS) (D), C3-PBI/SiO_2_ (5 equiv. TEOS) (E), C3-PBI/SiO_2_ (10 equiv. TEOS) (F), C3-PBI/SiO_2_ (100 equiv. TEOS) (G) and pure SiO_2_ (H).

We are in the following representatively discussing the results for C3–NH_2_ as amine. All other tested systems behave similarly, and the corresponding data can be found in the ESI.†Fig. 2 shows the ATR-FTIR spectra of all C3-PBI/SiO_2_ of different ratios of the two compounds (Fig. 2D–G), for reference hydrothermally prepared pure C3-PBI (Fig. 2C) and SiO_2_ (Fig. 2H), and the PBI precursors C3–NH_2_ and PBA (Fig. 2A and B). Already at 1 equiv. of TEOS, *i.e.*, 1 : 1 C3-PBI : SiO_2_, a series of narrow bands at ∼1595, ∼1580, ∼1400, ∼1315, ∼855, ∼810 and ∼745 cm^−1^, belonging to the vibrations of the aromatic perylene core,^17^ is present in the reaction product. Furthermore, the starting compounds are fully converted, since their typical modes are not visible anymore: The characteristic modes of PBA (*ν̃*_as_(C=O) ∼1770 cm^−1^ and *ν̃*_s_(C=O) ∼1730 cm^−1^) are fully absent (compare Fig. 2B for pure PBA). Also, the typical hydrogen bonding modes of NH_2_ groups of C3–NH_2_ (3300 and 3200 cm^−1^, Fig. 2A) are not present in the product. Instead, the characteristic *ν̃*(C=O) imide modes of PBIs are nicely visible at ∼1690 and ∼1650 cm^−1^ (Fig. 2C). The ATR-FTIR spectrum of reference SiO_2_ (Fig. 2H) shows three characteristics SiO_2_ modes, *i.e.*, ∼1100 cm^−1^ (Si–O–Si asymmetric stretching), ∼800 cm^−1^ (Si–O symmetric vibration), and ∼455 cm^−1^ (Si–O–Si bending). At C3-PBI/SiO_2_ 1 : 1 (Fig. 2C), the characteristic SiO_2_ modes are not visible, which we believe to be a consequence of their relative strength. For instance, C=O modes are intense modes, relative to which Si–O–Si modes are weaker. At 5 equiv. TEOS (Fig. 2E), characteristic SiO_2_ modes are not yet visible, but a slight baseline deviation at ∼1100 cm^−1^ can be vaguely discerned. At 10 equiv. (Fig. 2H) the Si–O–Si contribution is clearly visible at ∼1100 cm^−1^. At the highest amount of TEOS used (100 equiv., Fig. 2G), the ATR-FTIR spectrum mostly shows SiO_2_ modes. The major C3-PBI modes (at ∼1690 and ∼1650 cm^−1^) are present at all tested PBI : SiO_2_ ratios, and their intensity decreases with increasing amount of SiO_2_. Note that for the C3-PBI/SiO_2_ products of HTS employing 5, 10 and 100 equiv. (Fig. 2E–G), it becomes clear that *ν̃*(C=O) anhydride modes are present in all cases. This suggests that the formation of SiO_2_ from TEOS is slowing down the hydrothermal formation of C3-PBI. It is well-known that the hydrolysis and condensation of TEOS leads to gelation, with gel-strength increasing with *c* (TEOS). Therefore, it is conceivable that the PBI formation is slower in a stronger gel, due to slower reactant diffusion. Furthermore, it is also conceivable that increasing amounts of SiO_2_ lower the reactivity of R–NH_2_ and PBA (or partly hydrolysed PBA) through H-bonding with surface silanol groups. Overall, we conclude from ATR-FTIR that C3-PBI is formed in all cases, but at amounts of SiO_2_ >1 equiv. the precursors are not fully converted at the chosen *T*_R_ and *t*_R_ (200 °C, 24 h).

**Fig. 3:**
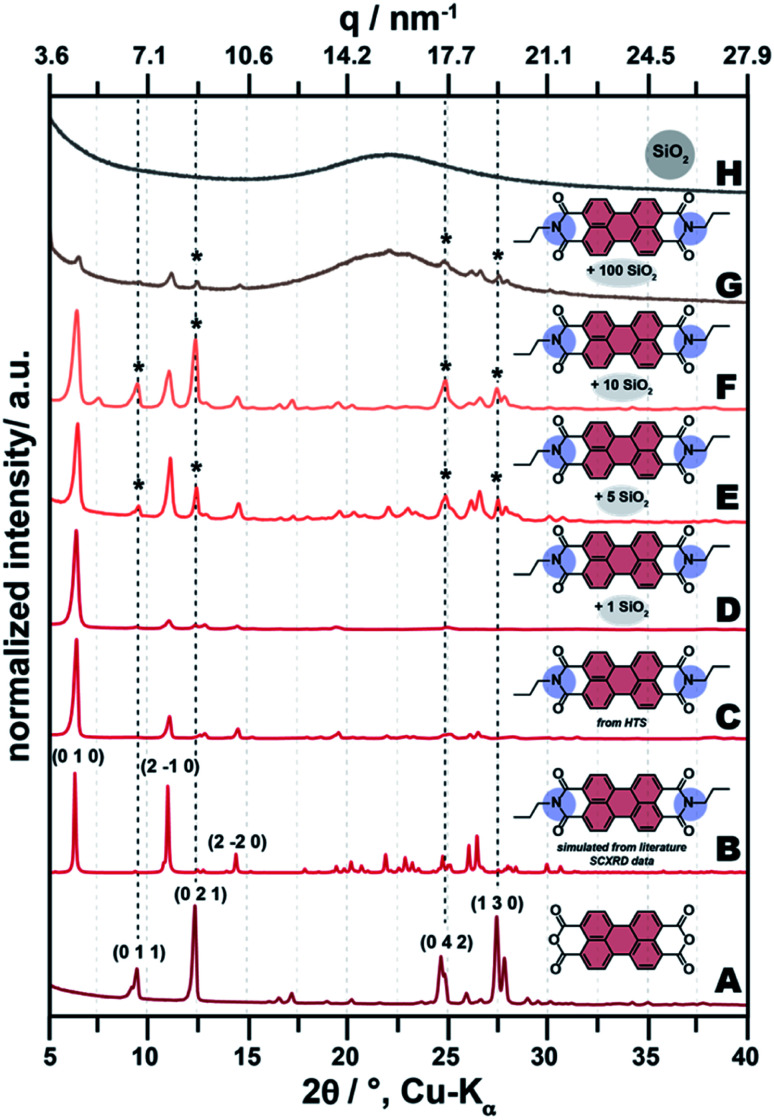
PXRD patterns of PBI-SiO_2_ samples and reference compounds. From bottom to top: starting compound PBA (A), C3-PBI PXRD pattern simulated from the literature SCXRD information file (CCDC: 1140265, CSD Refcode: DICLEG) (B), pure C3-PBI dye made by HTS (C), C3-PBI/SiO_2_ (1 equiv. TEOS) (D), C3-PBI/SiO_2_ (5 equiv. TEOS) (E), C3-PBI/SiO_2_ (10 equiv. TEOS) (F), C3-PBI/SiO_2_ (100 equiv. TEOS) (G) and pure SiO_2_ (H). *indicates reflections that stem from the presence of unreacted starting compound PBA.

HTS is known to promote crystallinity both for organic and inorganic compounds.^25^ Therefore, we investigated all samples by powder X-ray diffraction (PXRD). The C3-PBI dye synthesized hydrothermally for reference is highly crystalline as evinced by PXRD (Fig. 3C). Moreover, C3-PBI's diffractogram is clearly different from the starting compound PBA (Fig. 3A). Furthermore, the diffractogram of C3-PBI nicely fits the simulated diffractogram based on single crystal XRD data (SCXRD) reported in the literature (Fig. 3B).^30^ At 1 equiv. of TEOS used (Fig. 3D), the PBI/SiO_2_ samples present mainly reflections corresponding to pure C3-PBI. With increasing amount of SiO_2_, *i.e.*, 5 or 10 equiv. of TEOS used (Fig. 3E and F), reflections corresponding to the starting compound PBA are also present, evincing that not all precursors are converted to C3-PBI. This is corroborating ATR-FTIR spectroscopy data (*cf.*Fig. 2), where the C=O anhydride modes are still visible at >1 equiv. TEOS. At the highest amount of TEOS employed (100 equiv., Fig. 3G), an amorphous halo, centred around ∼22° (2*ϴ*, Cu-Kα) and corresponding to SiO_2_ is present in the diffractogram (*cf.* SiO_2_ reference, Fig. 3H). The fact that this halo is not visible at 1, 5 and 10 equiv. is explicable by the much lower intensity of the scattering of amorphous halos relative to the Bragg reflections of C3-PBI. In summary, PXRD of all samples corroborates the conclusions drawn from ATR-FTIR spectroscopy: (i) C3-PBI and SiO_2_ can be formed simultaneously by HTS at the chosen reaction conditions; (ii) with increasing amount of TEOS, there is incomplete conversion of the starting materials to C3-PBI.

**Fig. 4:**
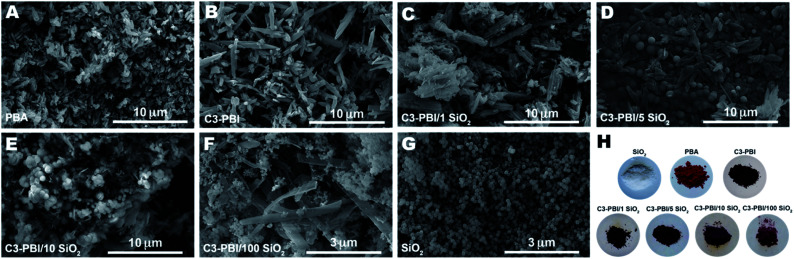
Micromorphology and aspect of C3-PBI/SiO_2_ samples and pure components. Shown are the SEM images of commercial PBA (A), pure C3-PBI dye made by HTS (B), C3-PBI/SiO_2_ (1 equiv. TEOS) (C), C3-PBI/SiO_2_ (5 equiv. TEOS) (D), C3-PBI/SiO_2_ (10 equiv. TEOS) (E), C3-PBI/SiO_2_ (100 equiv. TEOS) (F) and pure SiO_2_ (G). Photographs of the materials (H).

Next, we investigated all C3-PBI/SiO_2_, as well as reference C3-PBI and SiO_2_, by scanning electron microscopy (SEM) to check for the morphological consequences of the concomitant synthesis. Hydrothermally synthesized C3-PBI forms needle-like particles of *ca.* 5 μm in length (Fig. 4B). In contrast, SiO_2_ is obtained as near-monodisperse spherical particles of approximately 150 nm in diameter (Fig. 4G). When simultaneously generated by HTS, the samples' morphologies are somewhat a mixture of the individual components' morphologies, *i.e.*, needle-like particles coexist with spherical particles. Yet, the aspects of the needle-like and spherical particles are altered, evincing that the simultaneously occurring formation reactions influence each other also morphologically: At 1 equiv. of TEOS (Fig. 4C), the product is mainly composed of needle-like particles of 5–10 μm in length, thus slightly bigger than for pure C3-PBI. The needles' surfaces are rougher than those of pure C3-PBI. Moreover, no spherical particles are visible. This morphology suggests that SiO_2_ is covering the surfaces of C3-PBI needles. At 5 equiv. TEOS (Fig. 4D), spherical near-monodisperse particles of 2 μm in diameter (*i.e.*, significantly bigger than the SiO_2_ reference) coexist with *ca.* 5 μm long needles, which we believe to be C3-PBI. Increasing the amount of TEOS to 10 equiv. (Fig. 4E) generates: (i) a higher amount of spherical SiO_2_ particles over C3-PBI needles; (ii) a larger size distribution of the spherical particles (∼0.3–3 μm); (iii) two types of needles: very thin short needles (∼3 μm in length), and thicker longer needles (∼5–10 μm). Interestingly, at 100 equiv. of TEOS (Fig. 4F), the spherical particles are much smaller (∼50 nm) and coexist with ribbon shaped particles (∼5 μm in length). SEM analysis corroborates the PXRD results: (i) the addition of TEOS leads to an increased amount of silica spheres in the final material, which are seen in the powder diffractograms as amorphous halos; (ii) SEM analyses evince the presence of two distinct morphologies, typical for a mixture. The aspect of the C3-PBI/SiO_2_ HMs is shown Fig. 4H as photographs of the powders. While SiO_2_ and PBA are white and red powders, respectively, C3-PBI is of dark reddish to purple hue. All C3-PBI/SiO_2_ samples show purple colour that becomes the lighter the higher the SiO_2_ content, indicating the ‘dilution’ of the dye with silica.

**Fig. 5:**
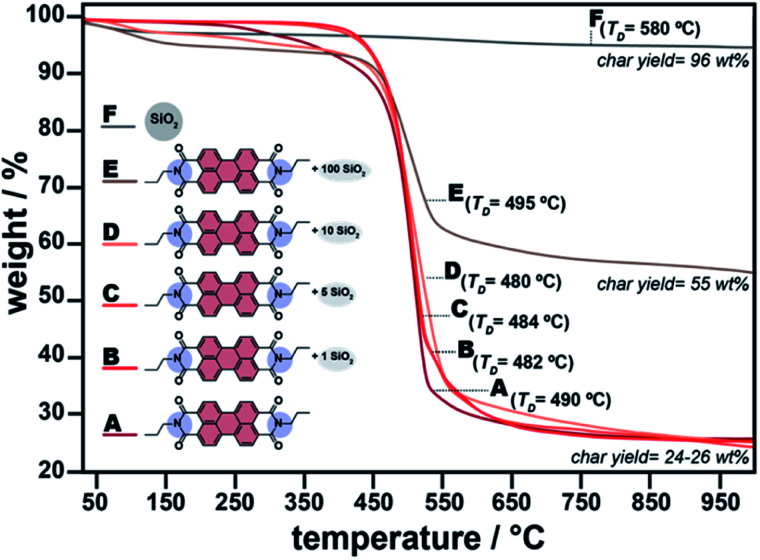
TGA of C3-PBI, SiO_2_ and their hybrids. Shown are the pure C3-PBI dye (A), C3-PBI/SiO_2_ (1 equiv. TEOS) (B), C3-PBI/SiO_2_ (5 equiv. TEOS) (C), C3-PBI/SiO_2_ (10 equiv. TEOS) (D), C3-PBI/SiO_2_ (100 equiv. TEOS) (E) and pure SiO_2_ (F).

Both chemical and structural analysis (ATR-FTIR spectroscopy, PXRD) as well as morphological analyses (SEM) and appearances of the materials allow for concluding that simultaneously generating C3-PBI and SiO_2_ is possible by HTS, and that the one-pot HTS affects the products. All analyses point at mixtures of the two components that likely do not, or only weakly, interact with each other. To finally ascertain that PBI/SiO_2_ are mixtures, we performed thermogravimetric analysis (TGA) in N_2_ atmosphere (Fig. 5). First, nearly all TGA traces show a first weight loss of approx. 4–7 wt% between r.t. and 150 °C, which we assign to physiosorbed water remaining from the HTS or stemming from adsorbed humidity from ambient atmosphere. Second, the TGA traces of C3-PBI and all PBI/SiO_2_ resemble each other with respect to showing a second weight loss at a decomposition temperature (*T*_D_) ∼480–490 °C (determined by the derivative method; ESI, Fig. S1†). This *T*_D_ corresponds well to the thermal stability limit of pure C3-PBI. Note that while the presence of SiO_2_ does not affect *T*_D_, the char yield (*i.e.* the remaining sample mass after decomposition), increases with the increase of TEOS. This indeed reflects the increasing amount of SiO_2_ in the mixture. The fact that *T*_D_ does not change with the presence of SiO_2_ once more points at a mixture of SiO_2_ and C3-PBI rather than a HM. Pure SiO_2_ prepared by HTS for comparison is thermally stable – as expected – until the highest measured temperature (1000 °C), with still 96 wt% of remaining sample mass. The loss of approx. 4 wt% corresponds to the desorption of physiosorbed H_2_O (below 150 °C) and dehydration of surface silanol (Si–OH) groups (occurring at ∼580 °C, also evinced through the derivative method – see ESI†).

In summary, it is possible to simultaneously form C3-PBI and SiO_2_ in nothing but hot water. The higher the amount of TEOS, the smaller the conversion of PBA to C3-PBI. The obtained C3-PBI/SiO_2_ materials are a mixture at the microscale, and while the concomitant synthesis affects the two constituents' morphologies, no synergistic materials properties are observed. Analogous results are obtained for all other tested systems (C5-PBI/SiO_2_, C8-PBI/SiO_2_, and C14-PBI/SiO_2_, see ESI Fig. S2–S7†).

### 2.2 HTS of class II *n*-alkyl-PBI@SiO_2_ hybrid materials

**Fig. 6:**
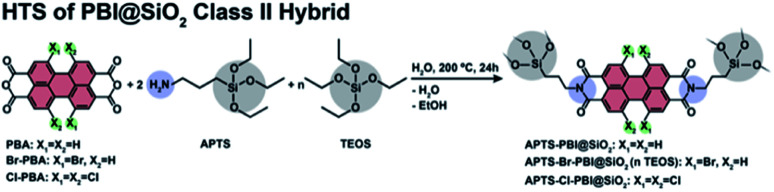
Synthesis of APTS-PBI@SiO_2_ (X_1_ = X_2_ = H), APTS-Br-PBI@SiO_2_ (X_1_ = Br, X_2_ = H) and APTS-Cl-PBI@SiO_2_ (X_1_ = X_2_ = Cl). *n*_TEOS_ was varied from 0, 2, 20 to 100 equivalents.

For generating synergistic properties between the organic dye and the inorganic SiO_2_ components, we performed a second set of experiments, using a bifunctional linker molecule. Specifically, we used the linker aminopropyltriethoxysilane (APTS), which comprises an NH_2_ function able to react with PBA as well as Si(OEt)_3_ functions able to co-condense to SiO_2_ (Fig. 6). Thus, covalent linking is guaranteed per design, if both the NH_2_ and the OEt functions condense. Hence, both obtaining class II HMs and truly synergistic properties is expected. Note that neither R–NH_2_ nor TEOS were employed, *i.e.*, APTS was both the only SiO_2_ and the only amine source. We refrained from using C_3_–NH_2_ and TEOS here in addition to APTS, as we expected this to lead to a coexistence of the desired class II HM and a PBI/SiO_2_ mixture. Moreover, we did not study a series of related linker molecules, as only APTS is commercially available, while the linkers corresponding to the R–NH_2_ employed in the previous section (aminopentyl-, aminooctyl-, and aminotetradecyltriethoxysilane) are not. The HTS conditions were kept the same as for the synthesis of the C3-PBI/SiO_2_ mixtures, *i.e.*, *t*_R_ = 24 h and *T*_R_ = 200 °C, and the concentrations of reactants were also kept. As we will show in the following, C3-PBI is found well dispersed inside the SiO_2_ matrix and indeed covalently linked to it.

**Fig. 7:**
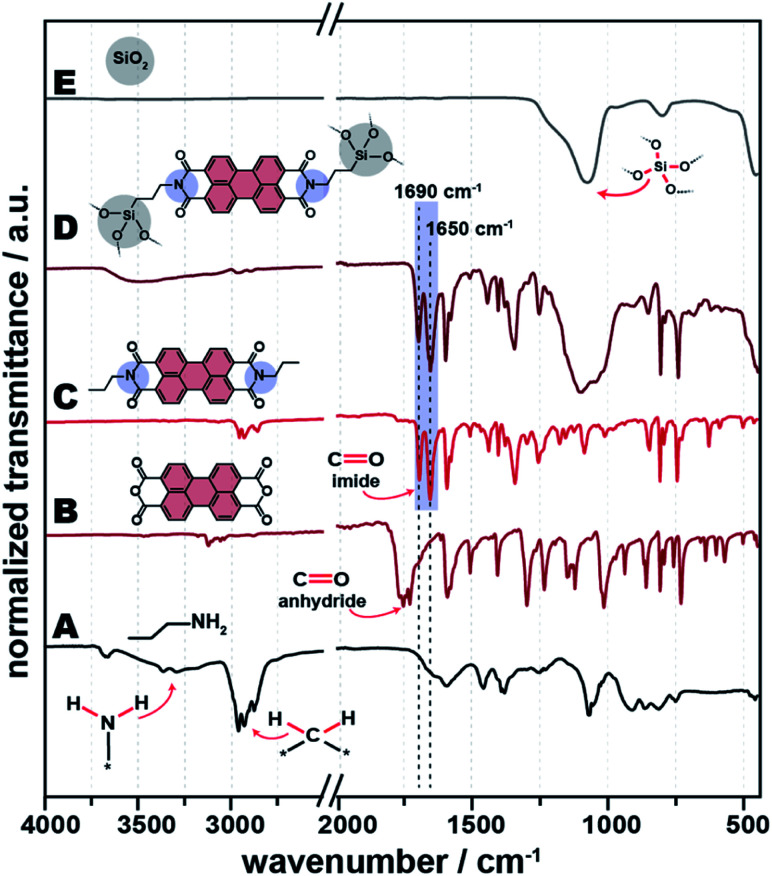
ATR-FTIR spectra of APTS-PBI@SiO_2_ and its precursors. Shown are the spectra of APTS (A), PBA (B), pure C3-PBI (C), APTS-PBI@SiO_2_ HM (D) and pure SiO_2_ (E).

For differentiating these and all subsequently described covalently linked HMs from the C3-PBI/SiO_2_ mixtures discussed in the previous section, they are subsequently designated as “APTS-PBI@SiO_2_”. ATR-FTIR analysis of the product reveals the presence of the characteristic imide C=O modes at ∼1690 and ∼1650 cm^−1^ (Fig. 7D), and the absence of the PBA's C=O anhydride modes (Fig. 7B). APTS-PBI@SiO_2_ (Fig. 7D) exhibits modes at ∼2950–2850 cm^−1^, corresponding to C–H stretching modes of the propyl spacers' methylene units. As C3-PBI also bears *n*-propyl functions, the reference PBI (Fig. 7C) features these modes, too. Furthermore, APTS-PBI@SiO_2_'s spectrum features a broad a Si–O–Si mode at 1100 cm^−1^, as well as Si–OH vibration modes (∼800 cm^−1^). Overall, ATR-FTIR analysis is already indicative for a covalent binding of the C3-PBI and SiO_2_ constituents to each other, for the presence of imide modes, which can only arise from APTS condensing with PBA and the presence of SiO_2_ modes, which can only stem from the hydrolysis and subsequent self-condensation of APTS.

**Fig. 8:**
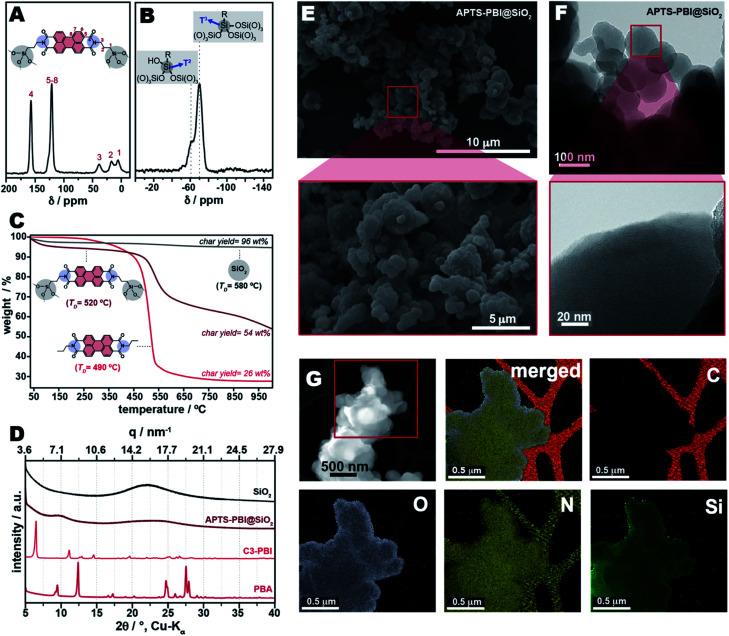
Characterizations of APTS-PBI@SiO_2_. Shown are the ^13^C CP/MAS NMR spectrum (A) as well as ^29^Si CP/MAS spectrum (B) of APTS-PBI@SiO_2_. Panel (C) shows the TGA curves of APTS-PBI@SiO_2_, C3-PBI and SiO_2_. PXRD patterns of PBA, C3-PBI, APTS-PBI@SiO_2_ and SiO_2_ are shown in (D). The SEM images of APTS-PBI@SiO_2_ are depicted in (E), and HR-TEM images are shown in (F). Panel (G) shows the EDX spectroscopy mapping images of APTS-PBI@SiO_2_ (C, N, O, and Si were selected for imaging. The merged figure is the sum of all signals).

To investigate the binding and structure of APTS-PBI@SiO_2_, we performed solid-state NMR analysis, specifically ^13^C and ^29^Si cross-polarized magic-angle spinning (CP/MAS) NMR, shown in Fig. 8. In the ^13^C NMR spectrum, the signals at 38 and 16 ppm (3 and 2 in Fig. 8A) are attributed to the methylene carbons in α- and β-positions to the imide nitrogen moiety, while the peak at 7 ppm (1 in Fig. 8A) is assigned to CH_2_ adjacent to Si. The relatively large peak width indicates the presence of alkyl moieties with multiple bonding angles and distances. The peak between 100–130 ppm confirms the presence of the perylene core within the structure of APTS-PBI@SiO_2_, while the peak at 155 ppm confirms the presence of the imide C=O moieties.

The ^29^Si CP-MAS NMR spectrum of APTS-PBI@SiO_2_ (Fig. 8B) shows two peaks at −70 ppm and −60 ppm, which are overlapping, and ascribed to T^3^ [RSi(OSi)_3_] and T^2^ [RSi(OSi)_2_(OH)] sites, respectively. The fact that T^2^ sites are present points at a relatively high amount of silanol groups, potentially stemming from (i) an incomplete conversion of APTS to SiO_2_ and/or (ii) a relatively high number of Si–OH prompted by the synthesis in water. The latter is conceivable through some degree of microphase separation during the synthesis, where the more apolar PBI components (and also *n*-propyl spacers) are rather found towards the inside of APTS-PBI@SiO_2_ particles, while SiO_2_ is rather found at the interface with water, thus towards the outer part of the particles. Further, ^29^Si NMR high-power decoupling with magic angle spinning (HPDEC-MAS) was performed (see ESI, Fig. S8†), revealing that less than 4% of the ^29^Si sites are constituted of Q^n^ groups (*i.e.*, Si atoms linked to four oxygen atoms). This confirms that the HTS did not affect the integrity of APTS, *i.e.* the aminopropyl group was not eliminated from the Si-centres.

Furthermore, APTS-PBI@SiO_2_ was investigated by TGA (Fig. 8C). The *T*_D_ of APTS-PBI@SiO_2_ is found at 520 °C, thus 30 °C higher than that of C3-PBI alone and in the C3-PBI/SiO_2_ mixtures (*T*_D_ ∼490 °C). This increase of *T*_D_ is a further strong indication for the successful obtainment of a class II HM, and a first synergistic property. Moreover, with 54 wt%, the char yield is much higher than for C3-PBI but similar to that of C3-PBI/SiO_2_ from 100 equiv. TEOS (char yield: 55 wt%), *cf.*Fig. 5E. Also, there is a second mass loss occurring between 650 °C and 1000 °C which corresponds to the condensation of remaining Si–OH (presence previously evinced through ^29^Si CPMAS NMR).

Next, we investigated APTS-PBI@SiO_2_'s morphology by SEM (Fig. 8E). The typical needle-like morphology of the starting compound PBA (*cf.*Fig. 4A) as well as pure C3-PBI (*cf.*Fig. 4B) is entirely absent. Instead, spherical particles of approx. 1.5 μm in diameter are found. These particles appear framboid and feature rough surfaces. The observed morphologies are fundamentally different from all C3-PBI/SiO_2_ mixtures, pure C3-PBI (needle-like particles) and SiO_2_ (spherical particles), and clearly represent a homogeneous morphology as opposed to the mixtures of two morphologies found for all C3-PBI/SiO_2_. Thus, also SEM analysis strongly corroborates the successful formation of a class II HM. The roundish morphologies also suggest the absence of crystallinity. High-resolution transmission electron microscopy (HR-TEM, Fig. 8F) supports this indication, through the absence of lattice planes. From HR-TEM it becomes clear that APTS-PBI@SiO_2_ contains near-spherical particles down to ∼100 nm in diameter. Furthermore, mostly aggregates of these nanoparticles are found. Hence, together with the roundish framboid morphologies observed in SEM, we conclude that APTS-PBI@SiO_2_ is composed of microscopic particles that are (intergrown/co-condensed) aggregates of nanoscopic particles. Energy dispersive X-ray (EDX) spectroscopy, performed during TEM, Fig. 8G, evinces the presence of Si, O, and N in all parts of the particles, indicating compositional homogeneity. Note that carbon is strongest found in the carbon grid used for TEM sample preparation (red in Fig. 8G). Furthermore, PXRD was performed and confirms the absence of Bragg reflections (Fig. 8D), which is in agreement with HR-TEM.

So far, we have shown for APTS-PBI@SiO_2_ HMs that (i) they can be synthesized hydrothermally in one-pot; (ii) they are true class II HMs with covalent linking between the components; and (iii) they exhibit synergistic materials features – so far shown with respect to thermal stability and morphology. While PXRD and TEM analysis evince amorphicity, one cannot draw conclusions from these analyses regarding molecular homogeneity *vs.* aggregation into, *e.g.*, a domain structure, of APTS-PBI@SiO_2_.

**Fig. 9:**
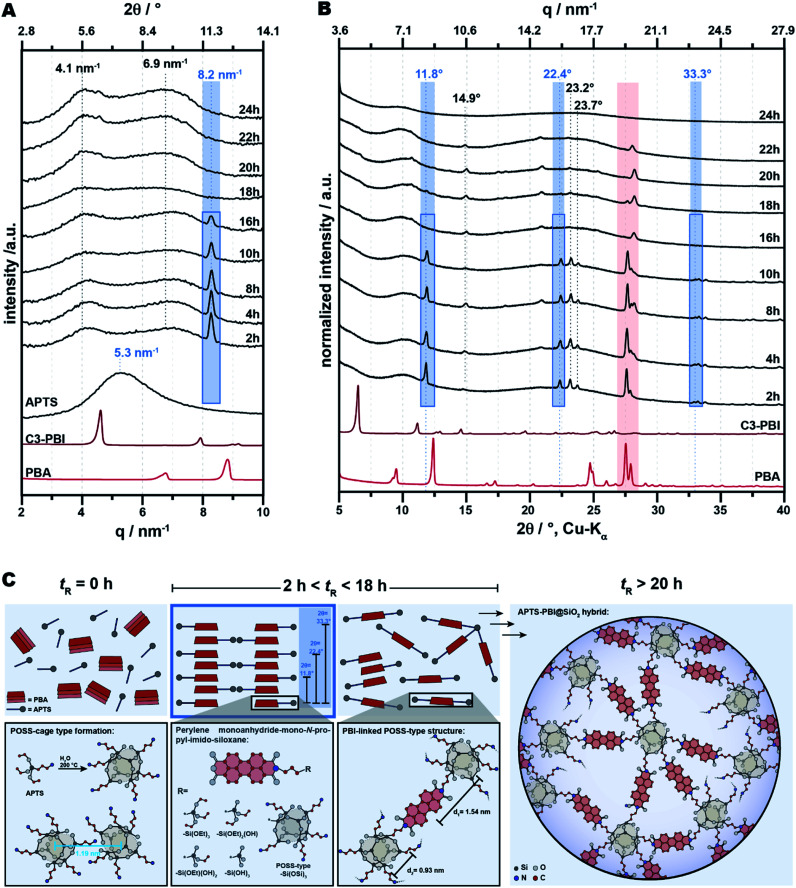
The APTS-PBI@SiO_2_ structure. (A) SAXS and (B) PXRD of the APTS-PBI@SiO_2_ at different times of reaction. The compound C3-PBI is shown in red for reference. APTS subjected to HTS (200 °C, 2 h) (blue curve). (C) Proposed arrangement of the APTS-PBI@SiO_2_ HM. PBA and APTS are combined under HTS, yielding a POSS cage-like structures containing PBI. Hydrogen atoms are omitted for clarity.

For investigating the molecular homogeneity, we performed small angle X-ray scattering (SAXS) analyses. We were here interested in the evolution of the molecular homogeneity with *t*_R_. Therefore, we prepared APTS-PBI@SiO_2_ from PBA and APTS at further *t*_R_ (in addition to *t*_R_ = 24 h), namely at *t*_R_ = 2, 4, 8, 10, 16, 18, 20, and 22 h. The ATR-FTIR spectra of all these samples are displayed in the ESI. SAXS curves of the products obtained at different *t*_R_ are shown in Fig. 9A (black curves). The curves of all APTS-PBI@SiO_2_ HMs present two main broad peaks centered at 4.1 nm^−1^ and 6.9 nm^−1^, respectively. Furthermore, a sharp Bragg peak at 8.2 nm^−1^ is present from the first experiment (*t*_R_ = 2 h) until *t*_R_ = 16 h and its intensity decreases with *t*_R_. In contrast, the two broad peaks at 4.1 and 6.9 nm^−1^ are present in all samples, with the second peak ∼6.9 nm^−1^ seemingly increasing in intensity with *t*_R_ and also slightly shifting to the left (bigger distances in real space). We initially hypothesized that the sharp reflection present until 16 h would correspond to yet unreacted PBA, conceivable through potentially slower reaction in a silica-type gel (see Discussion in Section 2.1 when increasing the amount of TEOS). However, when comparing the PXRD curves of the APTS-PBI@SiO_2_ hybrids synthesized at different *t*_R_ with the PXRD pattern of PBA (Fig. 9B), it becomes clear that the reflections differ significantly. Interestingly two reflections corresponding to unreacted PBA are in fact present (∼26.6° 2*ϴ*, Cu-Kα) until *t*_R_ = 22 h. Hence, PBA is indeed still present for several hours, however, the Bragg reflection at 8.2 nm^−1^ in SAXS corresponds to the reflection at 11.8°(2*ϴ*, Cu-Kα) in PXRD, and does not correspond to PBA. Even more interestingly, there are further sharp reflections in the PXRD patterns, namely at 14.9°, 22.4°, 23.2°, and 23.7° (2*ϴ*, Cu-Kα), plus several low intensity yet relatively sharp reflections at 2*ϴ* > 30° (Fig. 9B), which are all not corresponding to PBA. We hypothesize that in the early stages of the reaction, those PBA molecules that already react, do so first only on one side, *i.e.*, *via* one anhydride, with the NH_2_ function of APTS. Hence, these dimers are perylene monoanhydride-mono-*N*-propyl-imido-siloxane species (Fig. 9C). Since the rylene core of these dimers is only sterically hindered one side, the rylene cores of the dimers can pi-stack, and hence, in PXRD we find reflections in the range typical for pi-stack *d*-spacings 22.3°, 23.6°, and 23.9° (2*ϴ*, Cu-Kα), highlighted in blue in Fig. 9B). The first reflection (8.2 nm^−1^ in SAXS, 11.8° in PXRD) corresponds to twice a normal pi-stack distance (which would be in the range of 0.33–0.38 nm (plane–plane separation^31^). This seems reasonable given that the *N*-propyl-imido-siloxane would most likely point to opposite sides of the rylene in a stack (Fig. 9C). Fitting with this hypothesis is that the reflections at 11.8°, 22.4°, and 33.3° display a diffraction angle ratio of 1 : 2 : 3, with the reflections at 22.4° and 33.3° corresponding to the second and third order reflection of the 11.8° reflection. Furthermore, we speculate that these monoimide dimers are in fact distinct molecules, otherwise such sharp reflections are hardly conceivable. The siloxane moiety could be, *e.g.*, Si(OEt)_3_, or Si(OH)_3_, or a defined polyhedral oligomeric silsesquioxanes (POSS) moiety (Fig. 9C illustrates these possibilities). We believe that the siloxane moiety is most likely a polycyclic siloxane moiety, as Si(OR) functions are known to immediately hydrolyze to Si(OH) functions in an excess of water, and Si(OH) functions would also rapidly condense to siloxane oligomers.

The two broad reflections that at 4.1 nm^−1^ and 6.9 nm^−1^ are present in SAXS at any *t*_R_. We initially thought that they would correspond to the self-condensation product of APTS. One could expect the Si–O–Si formation to be faster than the imide condensation, and hence, we found it conceivable that these peaks already present at *t*_R_ = 2 h would correspond to the self-condensation product of APTS. It has been reported, that the hydrolysis and subsequent condensation of RSi(OR)_3_ such as APTS leads to oligomeric silsesquioxanes (RSiO_1.5_), such as POSS.^32^ POSS nanostructures have diameters in the range of ∼1–3 nm.^33^ To verify our hypothesis, we subjected pure APTS to HTS at 200 °C for 2 h. The corresponding SAXS pattern (Fig. 9A, blue curve) reveals one single broad peak centred at 5.3 nm^−1^ (*d* = 1.19 nm in real space). This is in agreement with the literature: Mortensen & Annaka have found one pronounced correlation peak corresponding to 1.9 nm in real space in SAXS experiments of concentrated aqueous solutions of ammonium hydrochloride-propyl terminated POSS,^34^ and Zhang *et al.* have reported one strong peak corresponding to 1.0–1.2 nm for a POSS-network in the solid-state.^35^ Hence, we conclude that the here performed hydrothermal condensation of APTS alone leads to POSS-type structures that in SAXS result in one broad and strong peak at *q* = 5.3 nm^−1^. This corresponds to 1.19 nm in real space and allows for concluding that these POSS-type structures are in the size range of ∼1 nm, which is in the lower typical range of such structures. Yet and strikingly, the two peaks at 4.1 nm^−1^ and 6.9 nm^−1^ (*d*_1_ = 1.54 nm and *d*_2_ = 0.93 nm, respectively, in real space) are not corresponding. Hence, we conclude that in the APTS-PBI@SiO_2_ hybrids, there is no significant presence of domains composed of the POSS-type product of APTS self-condensation, but instead, that monomimide species are already present at the shortest *t*_R_ (which is indeed confirmed through imide modes at *t*_R_ = 2 h in ATR-FTIR spectra, *cf.* ESI†). We hypothesize that the bimodality in SAXS arises, because the presence of the PBI units disturbs the POSS–POSS interaction. Hence, there is no strong peak (as in the APTS self-condensation product) visible, but instead two weak peaks from correlation within the units. As we believe, *d*_1_ = 1.54 nm corresponds to the distance between the centre of the PBI moiety and the centre of a connected POSS-type cage, which we calculated to be 1.57 nm. The coupling of the two segments, the PBI moiety and the POSS cage, leads to a weak peak in the scattering intensity from the correlation hole effect, with a maximum at about *q* = 4 nm^−1^, if the radii of gyration of both segments are inserted into the correlation function.^36,37^ Furthermore, we hypothesize that *d*_2_ = 0.93 nm corresponds to the lateral distance between propyl-spacers decorating the POSS-cages. Their calculated distances lie in the range of 0.9–0.95 nm, which is in good agreement with the observed 0.93 nm.

From SAXS and PXRD of APTS-PBI@SiO_2_ prepared at different *t*_R_, we conclude that (i) the hybrids are indeed homogeneous systems composed of PBI-linked POSS-type structures. (ii) Furthermore, in the early stages of their HTS, monoimide dimers are formed, which are forming crystalline domains. (iii) These domains must redissolve with time, in the sense that the pi-stacks are pushed apart by reaction of the remaining free anhydride, and hence, the Bragg refection in SAXS associated with the dimeric species disappears after 16 h. Fascinatingly, this shows that the system is to some extent still structurally dynamic during HTS. After long *t*_R_, features in SAXS and PXRD corresponding to PBI units interacting are gone in APTS-PBI@SiO_2_. Hence, we conclude that the PBI moities do not interact with each other. Additionally, these structural findings shed some light on a long-standing question in the field of polycondensation of trifunctional organosilanes of the type RSi(OR′)_3_. Despite the fact that the microstructure of polysiloxane network materials obtained by polycondensation of alkoxysilanes in the presence of H_2_O (as most prominently relevant in hydrolytic sol–gel processes) is predetermined at the early stages of polycondensation, little is known about early structures.^38^ In 1965, Brown and Brown *et al.*, respectively, studied the species that would form upon treatment of RSiCl_3_ with aqueous acetone.^39,40^ They observed that RSi(OH)_3_ would form rapidly, as to be expected. They also found, surprisingly, that these silanols would further condense to controlled and distinct polycyclic siloxane oligomers in a selective manner. These findings opposed the long-standing paradigm that polymerization of trifunctional silicones RSi(OH)_3_ would rapidly give complex mixtures of polycondensates. In an extensive chromatographic study of the products of the reactions of organosilanes of the type RSi(OMe)_3_ with H_2_O, Piana & Schubert also found low molecular weight oligomers at the early stages of condensation.^38^ Brown furthermore hypothesizes: “[…] *further condensation occurs to give “cured” silicone resins. These high polymers have usually been viewed as randomly connected three-dimensional network structures. From the present work it would appear more likely that their structures actually resemble irregularly branched strings of beads, in which the “beads” are the polycyclic blocks […] formed in the initial polycondensation*”.^39^ From the here discussed SAXS and PXRD results, we concur with Brown's hypothesis, and accordingly believe that the final PBI@SiO_2_ materials we here obtain correspond to networks of PBI-linked POSS-type cages, or similar polycyclic siloxane cages, as illustrated in Fig. 9C (right side).

**Fig. 10:**
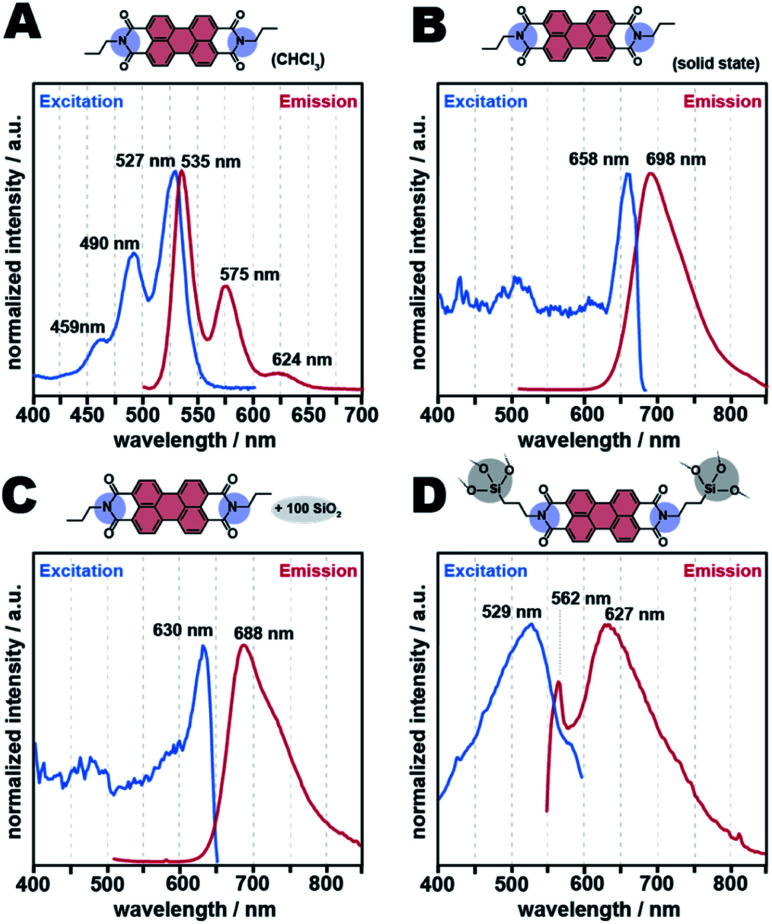
Excitation and emission spectra of C3-PBI, C3-PBI/SiO_2_ mixture and its class II hybrids APTS-PBI@SiO_2_. C3-PBI in CHCl_3_ (1 μmol L^−1^) spectrum (A), solid-state spectra of C3-PBI (B), C3-PBI/100 SiO_2_ (C) and APTS-PBI@SiO_2_ hybrid material (D).

The optoelectronic properties of PBIs in the solid-state are dominated by their interactions with each other. For instance, solid-state fluorescence of PBIs is often quenched due to the strong π-stacking interactions between neighboring molecules.^41^ The distance of a typical π–π interaction is of approx. 3.2 Å. This corresponds to approx. 26° (2*ϴ*, Cu-Kα). Reflections in this range are absent in both PXRD and SAXS of APTS-PBI@SiO_2_ synthesized at *t*_R_ > 20 h (Fig. 9A and B). Thus, we expected the HMs to show altered and potentially interesting optoelectronic properties. To investigate this aspect, we measured the solid-state emission and excitation of APTS-PBI@SiO_2_ by fluorescence spectroscopy, as well as, for comparison, those of the C3-PBI dye and the C3-PBI/SiO_2_ mixtures prepared in the previous section. The spectra are displayed in Fig. 10 and the therefrom extracted *λ*_abs_ and *λ*_ems_ are summarized in Table 1. We also performed solution absorption and emission spectroscopy of C3-PBI dissolved in CHCl_3_, displayed in Fig. 10A, which shows the for PBIs characteristics well-resolved vibronic fine-structure of three maxima (absorption: S_0_,_*ν*=0_ → S_1_,_*ν*=0_; S_0_,_*ν*=0_ → S_1_,_*ν*=1;_ S_0_,_*ν*=0_ → S_1_,_*ν*=2_ and emission: S_1_,_*ν*=0_ → S_0_,_*ν*=2_; S_1_,_*ν*=0_ → S_0_,_*ν*=1_; S_1_,_*ν*=0_ → S_0_,_*ν*=0_).^42,43^ The most intense absorption band appears at *λ*_abs_ = 527 nm, and the emission maximum at appears at *λ*_ems_ = 535 nm. Note that the spectra were recorded at very low concentrations in CH_3_Cl (*c* (C_3_-PBI) = 1 μmol L^−1^), so that π-stacking in solution can be neglected as becomes clear from the well resolved vibronic fine structure. In comparison, C3-PBI's absorption spectrum in the solid state (Fig. 10B and Table 1, entry 2) shows a significant red shift along with considerable band broadening due to π–π interactions. Both absorption and emission spectra feature a single broad peak, respectively, corresponding to *λ*_abs_ = 658 nm and *λ*_emi_ = 698 nm (Fig. 10B). The C3-PBI/SiO_2_ mixture (100 equiv. SiO_2_) shows basically the same absorption and emission spectra as C3-PBI alone in the solid state, with *λ*_abs_ = 630 nm and *λ*_emi_ = 688 nm, *i.e.*, the absorption is blue shifted by 28 nm and the emission is shifted by 10 nm with respect to C3-PBI without SiO_2_ (*λ*_abs_ = 658 nm, *λ*_emi_ = 698 nm), *cf.*Fig. 10C and Table 1, entry 3. For the breadth of the emission and absorption peaks, we consider *λ*_abs_ and *λ*_emi_ as similar in C3-PBI and C3-PBI/SiO_2_ and conclude that the presence of SiO_2_ in the mixture does not significantly affect C3-PBI's absorption and fluorescence spectra. However, for APTS-PBI@SiO_2_, we find that the absorption and emission behavior are different. While there is still no vibronic fine structure, the emission maximum is significantly blue-shifted (by 71 nm) with respect to C3-PBI in the solid state. Intrigued by these altered fluorescence features, we were subsequently aiming at improving/tuning them. Towards this goal, we investigated two strategies: (i) changing the electronic properties of the rylene core by employing bay-substitution, together with (ii) diluting the PBI moieties even further by increasing the SiO_2_ contents in the HMs. The results are discussed in the following section.

**Table 1:** Estimated direct band gap energies, values of *λ*_e*x*citation_ and *λ*_emission_ of the solid materials synthesized by HTS

Entry	Material		Band GAP^a^ (eV)	*λ*_abs_ (nm)	*λ*_ems_ (nm)
1	Pure SiO_2_ made by HTS		4.45	—	—
2	C3-PBI	Solid	1.68	658	698
		In CHCl_3_	2.27	459; 490; **527**	**535**; 575; 624
3	Mixture of C3-PBI/SiO_2_		1.77	630	688
4	APTS-PBI@SiO_2_		2.08	529	562; **627**
5	C3-Br-PBI	Solid	n.d.	638	692
		In CHCl_3_	n.d.	461; 490; **527**	**543**; 579
6	APTS-Br-PBI@SiO_2_		1.70	491; **552**	**538**; 582
7	APTS-Br-PBI@SiO_2_ (2 equiv. TEOS)		1.40	485; **531**	**547**; 574; 618
8	APTS-Br-PBI@SiO_2_ (20 equiv. TEOS)		1.38	481; **528**	**548**; 575; 618
9	APTS-Br-PBI@SiO_2_ (100 equiv. TEOS)		1.28	496; **530**	**550**; 589; 636

a *E*_g_ were calculated from Tauc's plot, and can be found in the ESI. The strongest *λ*_abs_ and *λ*_ems_ observed (global maxima) are highlighted in bold.

### 2.3 HTS of bay-substituted PBI@SiO_2_ class II hybrid materials

PBIs can be structurally modified either at the peri positions (*i.e.*, in position 1 and 8 of the naphthalene cores), for instance when generating PBIs from PBA and RNH_2_ by incorporating different *N*-substituents through the used amines, or in the so-called “bay” positions (highlighted in green in Fig. 6), *e.g.*, by the attachment of electron-withdrawing groups (EWGs). Bay-substitution typically generates non-planar rylene cores. Moreover, through the bulkiness of the substituents at these sterically encumbered positions intermolecular π–π interactions are reduced. However, on the downside the non-planarity of the rylene cores of bay-substituted PBIs in solution generates (i) broadening of the lowest energy absorption band, and (ii) a reduction of the definition of vibronic fine structure.^41^ When strong EWGs, such as Br or Cl, are used, the corresponding bay-substituted PBAs are more reactive than unfunctionalized PBA, and also, the obtained PBI derivatives are more electron deficient, leading to lower reduction potentials.

**Fig. 11:**
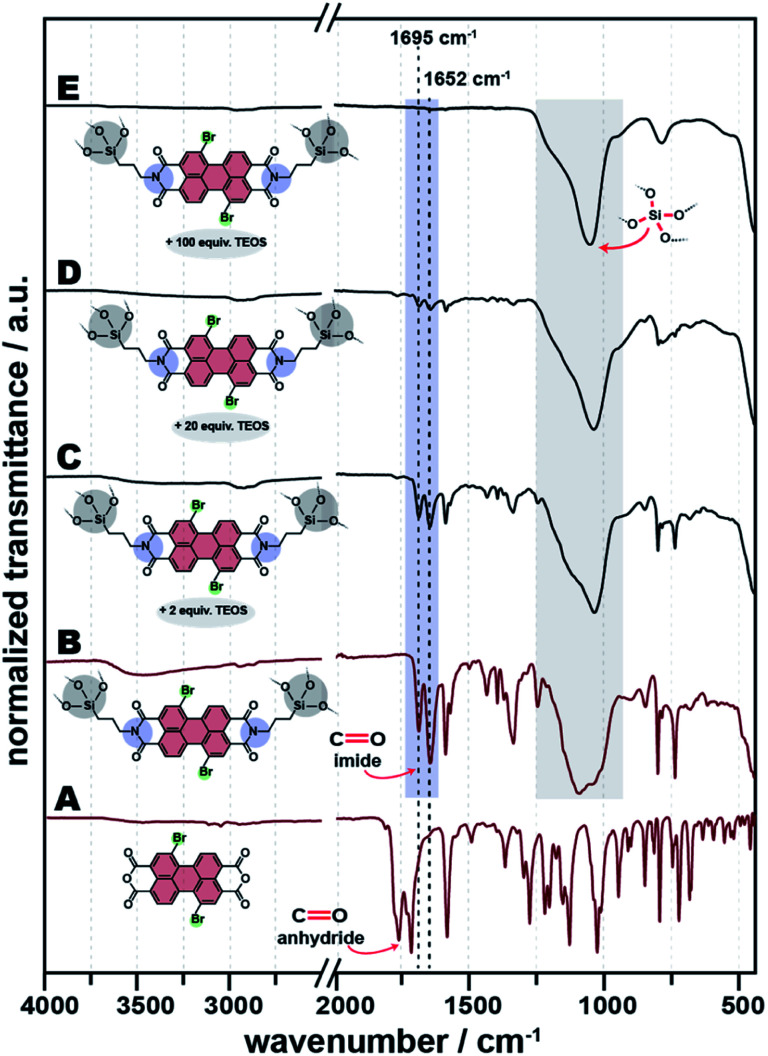
ATR-FTIR of starting compound Br-PBA (A), APTS-Br-PBI@SiO_2_ (2 APTS: 1 Br-PBA) (B), TEOS-APTS-Br-PBI@SiO_2_ (2 APTS: 2 TEOS: 1 Br-PBA) (C), TEOS-APTS-Br-PBI@SiO_2_ (2 APTS: 20 TEOS: 1 Br-PBA) (D) and TEOS-APTS-Br-PBI@SiO_2_ (2 APTS: 100 TEOS: 1 Br-PBA) (E).

Here, we employed 1,7-dibromo-PBA (Br-PBA), and 1,6,7,12-tetrachloro-PBA (Cl-PBA), with APTS (Fig. 6). First, we performed the HTS of APTS-Br-PBI@SiO_2_ and APTS-Cl-PBI@SiO_2_ as previously at *T*_R_ = 200 °C and *t*_R_ = 24 h, and using 1 equiv. of Br-PBA or Cl-PBA, respectively, and 2 equiv. of APTS as the only amine and SiO_2_ source. The products from using Cl-PBA (which is itself of orange color) became black after HTS, and ATR-FTIR spectroscopy shows that the starting Cl-PBA is still unreacted and the products are composed of a mixture of unreacted Cl-PBA and SiO_2_ (ESI, Fig. S10†). Hence, we conclude that Cl-PBA is not amenable to the herein employed HTS conditions. However, the APTS-Br-PBI@SiO_2_ HM was successfully obtained. The ATR-FTIR spectrum of the product is shown in Fig. 11B and features the characteristic imide modes at 1695 and 1652 cm^−1^. Structural analysis by PXRD reveals an amorphous material with two main halos centred at 7° and 22° (2*ϴ*, Cu-Kα), while SEM images reveal homogeneous roundish particles, similar to the non-bay substituted HMs (ESI, Fig. S11 and S12†).

Next, we adjusted the HTS of APTS-Br-PBI@SiO_2_ by adding TEOS to the precursors. Specifically, we added 2, 20 and 100 equiv. of TEOS to the mixture containing 1 equiv. of Br-PBA and 2 equiv. of APTS. ATR-FTIR spectra of the products are depicted in Fig. 11C–E. The PBIs' imide modes are clearly visible in the APTS-Br-PBI@SiO_2_ HMs made with 2 and 20 equiv. of TEOS (Fig. 11C and D, respectively), yet are barely visible when 100 equiv. TEOS are used (Fig. 11E). The SiO_2_ modes are clearly visible in all spectra and in fact dominate the spectra. Further characterizations (PXRD, SEM, and TGA) were performed and gave very similar results to the APTS-PBI@SiO_2_ HMs discussed previously (see ESI, Fig. S11–S13†).

**Fig. 12:**
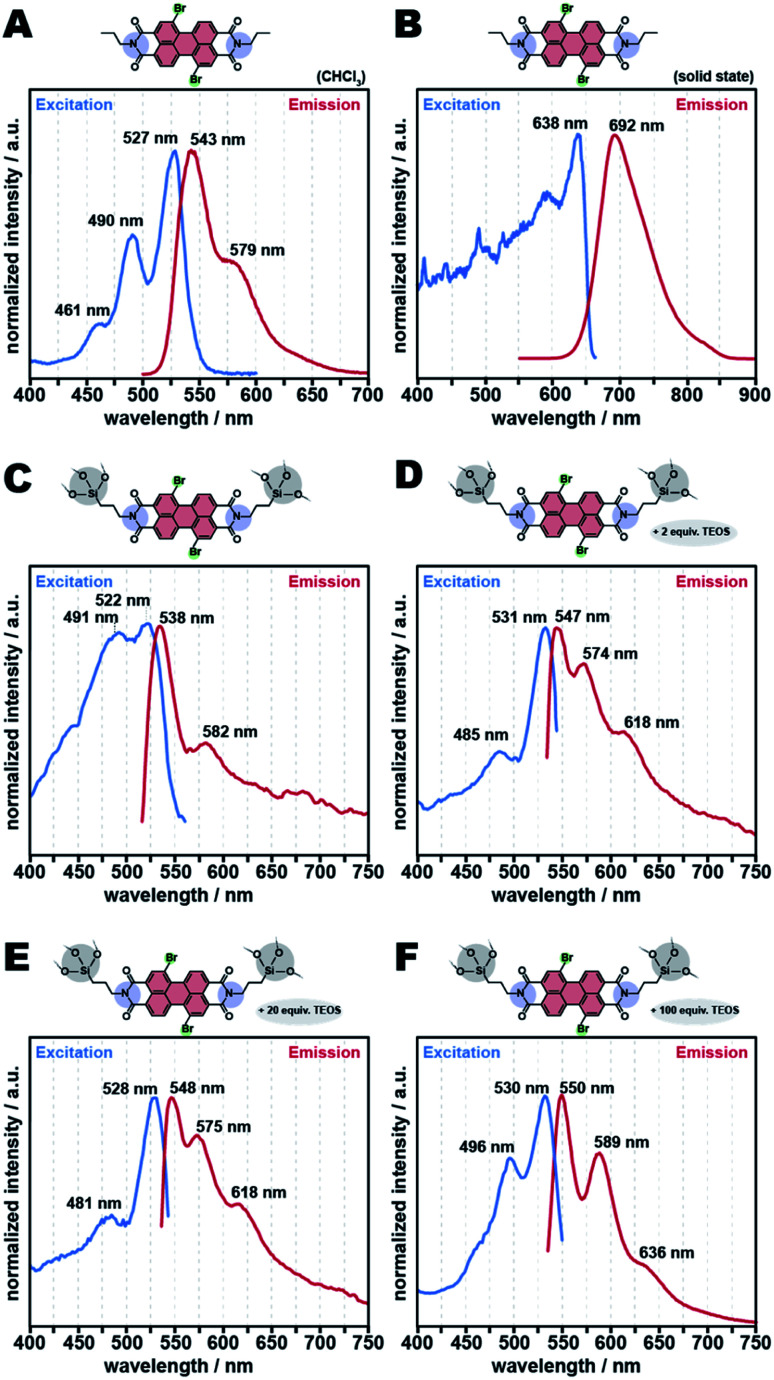
Excitation and emission spectra of class II hybrids APTS-Br-PBI@SiO_2_ and TEOS-APTS-Br-PBI@SiO_2_. C3-Br-PBI in CHCl_3_ (1 μmol L^−1^) spectrum (A), solid-state spectrum of C3-Br-PBI (B), APTS-Br-PBI@SiO_2_ (2 APTS: 1 Br-PBA) (C), TEOS-APTS-Br-PBI@SiO_2_ (2 APTS: 2 TEOS: 1 Br-PBA) (D), TEOS-APTS-Br-PBI@SiO_2_ (2 APTS: 20 TEOS: 1 Br-PBA) (E), and TEOS-APTS-Br-PBI@SiO_2_ (2 APTS: 100 TEOS: 1 Br-PBA) (F).

The absorption and fluorescence emission properties of all APTS-Br-PBI@SiO_2_ class II HMs (Fig. 12) were collected at the same parameters used for APTS-PBI@SiO_2_ (Fig. 10). All *λ*_abs_ and *λ*_ems_ extracted from these spectra are summarized in Table 1 (entries 5–9). APTS-Br-PBI@SiO_2_ (no TEOS employed) shows *λ*_abs_ = 522 nm and *λ*_ems_ = 538 nm (Fig. 12C). Interestingly, the emission spectrum now features two maxima – as does the Br-PBI alone in solution in CHCl_3_, yet the curve is not well resolved and somewhat noisy. Furthermore interesting is, that the emission maxima are neither strongly red nor blue shifted with respect to the position of the maxima of the dye in solution in CHCl_3_. In fact, they are blue shifted by as little as 5 nm. However, there is a significant red shift of Br-PBI's emission maximum in the solid state compared to the dye in solution in CHCl_3_, because in the solid state the Br-PBIs still pi-stack with each other. The fact that APTS-Br-PBI@SiO_2_ already without the addition of TEOS does not feature shifts compared to the solution in CHCl_3_ (*i.e.*, Fig. 12C compared to Fig. 12A) points towards an already better dissolution of dye inside the matrix than in the absence of the Br substituents (*i.e.*, in APTS-PBI@SiO_2_; Fig. 10D compared to Fig. 10A). In their respective HTS, both HMs were prepared at identical concentrations of precursors. To investigate the seemingly better dissolution of the dye in the brominated case, we calculated the dyes' concentrations in SiO_2_ from TGA analyses. Interestingly, the final concentrations of dyes inside SiO_2_ vary significantly between the brominated and the non-brominated HM (ESI, Table S2;†*c*(APTS-PBI@SiO_2_) in SiO_2_: 36 wt% = 7.2 mol%; *c*(APTS-Br-PBI@SiO_2_) in SiO_2_: 27 wt% = 4.2 mol%). Consequently, at the same reaction parameters, APTS-Br-PBI@SiO_2_ is less incorporated in SiO_2_ than APTS-PBI@SiO_2_. This seems to lead to less dye aggregation in APTS-Br-PBI@SiO_2_ compared to APTS-PBI@SiO_2_. We believe this to be the main reason for the differences in shifts of the fluorescence maxima.

Most intriguingly, the absorption and fluorescence properties of the APTS-Br-PBI@SiO_2_ HMs containing higher amounts of SiO_2_ (through TEOS addition, Fig. 12D–F) are significantly improved compared to APTS-Br-PBI@SiO_2_ synthesized without TEOS. All present two well-defined excitation maxima at ∼490 and 530 nm and three well-defined emission maxima at ∼548, 585 nm, and ∼620 nm (see also Table 1, entries 6–9). Consequently, the solid-state fluorescence spectra of these further diluted HMs are even better – with respect to well-resolved vibronic fine structures – than Br-PBI in solution in CHCl_3_. We hypothesize that these improvements are caused by increasing “dilution” of the C3-PBI units with increasing SiO_2_ content. Furthermore, the higher the amount of TEOS used, the more intense is the solid-state fluorescence (*cf.* ESI, non-normalized spectra, Fig. S14†). We calculated the quantum yield (QY) of the APTS-Br-PBI@SiO_2_(100 equiv. TEOS) to be 0.7% (ESI, Fig. S15†), which is surprisingly low. First, this could be a consequence of the high viscosity and polarity of SiO_2_ (and hence altered relaxation of the dye in the matrix). Second, and more importantly, the QY determination suffers strongly from scattering of both incident and emitted light by the materials, as they are microscopic/nanoscopic whiteish powders. In our opinion, QYs can therefore at this point not be commented on satisfactorily.

Next, we calculated the band gaps (*E*_g_) of the hybrids and their individual components – all made by HTS – by Tauc's relationship from UV-Vis reflectance (*cf.* ESI, Fig. S16† and Table 1). The band gap of SiO_2_ generated by HTS was found to be 4.45 eV (entry 1), which is in agreement with values found in the literature for hydrothermally synthesized silica nanoparticles.^44^ C3-PBI showed *E*_g_ of 1.68 and 2.27 eV in the solid state and in solution, respectively (entry 2). These values are also in the typical range for PBI dyes.^45^ The bandgaps of both the PBI/SiO_2_ mixture and the class II HMs in Table 1 were found to be in the range of PBI dyes. Thus, one can conclude that the electronic transitions of the PBI moieties were not affected by the presence or covalent bonding to SiO_2_. However, *E*_g_ of class II APTS-Br-PBI@SiO_2_ synthesized in the presence of TEOS decreases with increasing amount of TEOS used (and thus of SiO_2_ in their composition). For instance, APTS-Br-PBI@SiO_2_ (entry 6) shows *E*_g_ = 1.70 eV, whilst APTS-Br-PBI@SiO_2_ made with 100 equiv. TEOS shows *E*_g_ = 1.28 (entry 9). We hypothesize that the higher the relative amount of SiO_2_, the higher the polarity of the medium in which the PBI units are “dissolved”, and thus, the changes in bandgap are reflecting solvatochromism in the solid-state. In summary, it is, besides APTS-PBI@SiO_2_ HMs, also possible to obtain class II HMs using Br-PBA. Through implementing bay substitution, it is further possible to fine-tune the HMs' optoelectronic properties. Moreover, APTS-Br-PBI@SiO_2_ HMs of different compositions can be obtained, without significant shifts of *λ*_abs_ and *λ*_ems_, which is likely related to relatively good dissolution of the Br-PBI in SiO_2_. At the same time the emission's vibronic fine structures are well resolved, which is not the case for APTS-PBI@SiO_2_. Furthermore, the band gaps are decreasing with increasing amount of SiO_2_, as calculated from UV-Vis reflectance measurements, which we hypothesize to be a consequence of increasing polarity through higher amounts of SiO_2_.

Given that the PBIs electronic transitions are retained in the class II hybrids (*e.g.*, the vibronic fine-structures) and that the class II HMs feature solution-like properties, we were intrigued to explore if these class II hybrids could be employed in an application known for dissolved PBIs, that would profit from their solid-state nature. We explored the HMs application as photoredox catalysts in the reduction of aryl halides, which for PBIs has to our best knowledge to date exclusively been performed using dissolved PBIs, as described in the following and final section.

### 2.4 Applications of APTS-Br-PBI@SiO_2_ as solid photoredox catalysts

**Fig. 13:**
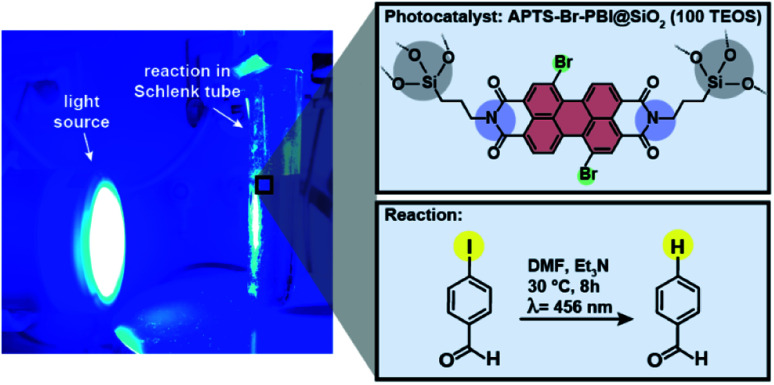
Photoreduction of 3-iodobenzaldehyde in the presence of the hybrid material APTS-Br-PBI@SiO_2_ (100 TEOS). The reaction was performed with 1 equiv. of iodobenzaldehyde (0.1 mmol); 8 equiv. of Et_3_N; 0.1 equiv. of C3-Br-PBI moiety (*ca.* 80 mg of APTS-Br-PBI@SiO_2_ (100 TEOS)), in 10 mL of DMF. A Kessil® lamp (*λ* = 456 nm) was placed at 6 cm distance, and temperature kept at 30 °C for 8 h.

PBIs are an attractive class of electron-deficient fluorescent organic photoredox catalysts. They have been shown to be strong photoreductants for difficult-to-reduce substrates, such as aryl halides.^46–48^ Yet, PBI photoreduction catalysts are typically used in solution, which naturally makes their recovery and reuse difficult. We performed the photoreduction of an aryl halide using the PBI@SiO_2_ hybrid material with the strongest solution-like fluorescence: APTS-Br-PBI@SiO_2_ at 100 equiv. TEOS. The reaction was performed by irradiating a mixture of 3-iodobenzaldehyde, APTS-Br-PBI@SiO_2_ (100 equiv. TEOS) as catalyst, and Et_3_N with blue light (456 nm) in DMF as solvent (Fig. 13). The reduction product benzaldehyde was obtained after 8 h hours with 89% of conversion (entry 5, Table 2), while the same PBI in its free form reaches 92% of conversion (entry 2, Table 2). Control experiments (entries 1, 3 and 4, Table 2) confirmed that the HM as photocatalyst, and light irradiation are necessary for the photoreduction reaction to occur. To test the stability and reusability of our photoredox catalyst, we recovered it from our initial reaction, and reused it in two more cycles of the same reaction (entries 6 and 7, Table 2). The results indicate that the conversion activity decreased somewhat, but is still above 70%, indicating that the catalyst keeps its features after three cycles. The catalyst structure was retained as confirmed by PXRD, ATR-FTIR, TGA and SEM after each reaction cycle (ESI, Fig. S17†).

**Table 2:** Photoreduction of 3-iodobenzaldehyde to benzaldehyde

Entry	Catalyst & conditions^a^	Conversion^b^ (mol%)
1	Pure SiO_2_ made by HTS	—
2	Pure C3-Br-PBI made by HTS	91.7 ± 1.2
3	Without any catalyst	—
4	APTS-Br-PBI@SiO_2_ (100 TEOS) without light	—
5	TEOS-APTS-Br-PBI@SiO_2_ (100 TEOS) 1^st^ cycle	88.6 ± 0.8
6	TEOS-APTS-Br-PBI@SiO_2_ (100 TEOS) 2^nd^ cycle	83.9 ± 0.6
7	TEOS-APTS-Br-PBI@SiO_2_ (100 TEOS) 3^rd^ cycle	70.4 ± 1.1

a Experiments were performed under LED Lighting (*λ* = 456 nm) from Kessil®, except entry 4.

b Conversions were calculated from GC measurements. *cf.* ESI for Experimental details.

## 3. Conclusions

We have herein explored the truly concomitant one-pot hydrothermal synthesis of PBIs and SiO_2_, obtained as product mixtures, as well as of covalently linked PBI@SiO_2_ HMs. At appropriate reaction conditions, full conversion of the precursors to products is obtained. The approach is green compared to date reported syntheses of comparable materials, as the products are achieved in one-pot reactions using nothing but the precursors and water as medium and releasing nothing but H_2_O and EtOH as byproducts. We prepared *n*-alkyl-PBI/SiO_2_ materials with *n*-alkyl = *n*-propyl, *n*-pentyl, *n*-octyl, and *n*-tetradecyl, which were identified as a mixture of the components at the microscale through ATR-FTIR spectroscopy, SEM, and TGA. The concomitant synthesis affects both constituents' morphology, and as the materials are mere mixtures, no synergistic materials properties are observed. For generating synergistic properties between the organic dye and the inorganic SiO_2_ network, we employed APTS, which comprises both an amine function able to react with the perylene anhydride precursors as well as three Si(OEt) functions able to hydrolyze and self-condense towards SiO_2_. Through employing APTS and PBA as the only precursors, class II HMs, with the PBI dye covalently bonded to the inorganic SiO_2_ matrix were successfully obtained. These materials were characterized by ATR-FT-IR spectroscopy, TGA, PXRD, SEM, HR-TEM, ss-NMR, SAXS and UV-Vis absorption and fluorescence emission spectroscopy. Synergistic materials properties were found with respect to thermal stability and optoelectronic properties. The PBI@SiO_2_ HMs show a homogenous organization down to the molecular level, and their formation entails crystalline monoimide intermediates. Based on PXRD and SAXS data, we hypothesize that these are composed of one POSS-type cage connected *via* a propylimide to a one perylene moiety, and furthermore that these dimers connect to the final HMS, which correspond to an amorphous network of POSS-type cages connected by perylene bisimides. Furthermore, we expanded the synthesis of these class II HMs to bay-substituted Br-PBIs@SiO_2_, and successfully explored the increase of the SiO_2_ portion. For PBI@SiO_2_, the optoelectronic properties in the solid state are not solution-like, but the fluorescence maxima are shifted with respect to both the dye alone in solution and the dye alone in the solid state. For Br-PBIs@SiO_2_ class II HMs, the optoelectronic characteristics are retained and solution-like. Interestingly, with increasing amount of SiO_2_, their band gaps decrease, which we hypothesize to be corresponding to solvatochromism in the solid-state. We have explored the synthesized hybrids for a PBI application that has to date exclusively employed dissolved dyes, *i.e.*, their use as photoredox catalysts in the photoreduction of aryl halides to aldehydes. The HMs yield conversions in the range of dissolved PBI catalysts, and can be separated from the reaction mixture by simple centrifugation, and reused at only minor decrease in activity.

Comparing our HTS of PBIs/SiO_2_ (mixtures) and especially PBIs@SiO_2_ (class II HMs), it becomes clear that to date several PBI dyes@M_*x*_O_*y*_ have been reported, but exclusively achieved through multistep synthesis and purification (see ESI,† comparison table). To the best of knowledge, none of the reported hybrids are made by one-pot methods. Moreover, the HMs made by conventional routes mostly suffer from aggregation of the individual components as major drawback. In contrast, we could here generate nanoscopically intertwined phases. We hope that our here reported simple and green approach, together with the observed intriguing materials properties, will set the basis for HTS of PBI dyes@M_*x*_O_*y*_ hybrid materials a promising strategy towards new optoelectronic materials.

## Author contributions

HMM performed all materials synthesis and characterization by ATR-FT-IR spectroscopy, SEM, TGA, PXRD, absorption and emission spectroscopy, UV-Vis reflectance measurements, as well as the photoredox experiments and GC-MS analysis. MMU designed and supervised this project. HP performed SAXS measurements and interpretation. HMM and MMU analyzed all data and wrote the manuscript, with input from HP.

## Conflicts of interest

There are no conflicts to declare.

## Supplementary Material

TA-010-D1TA03214C-s001
