# Supplementary material for: Green hydrothermal synthesis yields perylenebisimide–SiO2 hybrid materials with solution-like fluorescence and photoredox activity
Source: J Mater Chem A Mater. 2022 Jun 13;10(24):12817–31. doi: 10.1039/d1ta03214c (PMC9211763; doi:10.1039/d1ta03214c)
Supplement: TA-010-D1TA03214C-s001 [file TA-010-D1TA03214C-s001.pdf]

Electronic Supplementary Material (ESI) for Journal of Materials Chemistry A.
This journal is © The Royal Society of Chemistry 2022

Green Hydrothermal Synthesis Yields Perylenebisimide-SiO_2 Hybrid Materials with Solution-Like Fluorescence and Photoredox Activity

Hipassia M. Moura[a,b], Herwig Peterlik[c], and Miriam M. Unterlass[a,b*]

[a] Universität Konstanz, Department of Chemistry, Solid state chemistry, Universitätsstrasse 10, D-78464 Konstanz, Germany.

[b] CeMM Research Center for Molecular Medicine of the Austrian Academy of Sciences, Lazarettgasse 14, 1090 Vienna, Austria.

[c] Faculty of Physics, University of Vienna, Boltzmanngasse 5, 1090, Vienna, Austria.

ELECTRONIC SUPPORT INFORMATION

INDEX

1. State-of-the-art on PBI@M_xO_y hybrid materials 2
2. Experimental Details 7
   2.1 Chemicals 7
   2.2 Synthesis 7
   2.3 Characterizations 9
3. Results 10
   3.1. The simultaneous synthesis of n-alkyl-PBI and SiO_2 10
   3.2. HTS of Class II n-alkyl-PBI@SiO_2 hybrid materials 14
   3.3. Quantum yield 21
   3.4. Band gap calculation 22
   3.5. Photocatalysis 23
4. References 38

1. State-of-the-art on PBI@SiO_2 hybrid materials

In Table S1 we reviewed perylene bisimides@SiO_2-based hybrid materials found in literature, as well as the current state of their synthesis methods, and their features regarding solid-state fluorescence and possible applications. The different synthetic strategies towards organic-inorganic hybrid materials consist of:

(**A**) Strategies in which both organic and inorganic components are pre-made (e.g. dye loading into the matrix by gas/liquid sorption; mechanical mixing; ion exchange).

(**B**) Strategies in which one of the components is pre-made and the second component is formed subsequently (1: the dye is synthesized inside the matrix or 2: the matrix is made around the pre-made dye).

(**C**) Strategies in which the organic component needs to be modified to be incorporated in the pre-made inorganic component (1: the pre-made dye is modified with an alkoxy-group; then 2: sol-gel hybrid from hydrolysis of the modified-dye; 3: grafting of the modified-dye into the pre-made matrix).

Table S1. (This table has been updated) Comparison of PBI dyes@SiO_2 hybrid materials (HMs) found in literature.

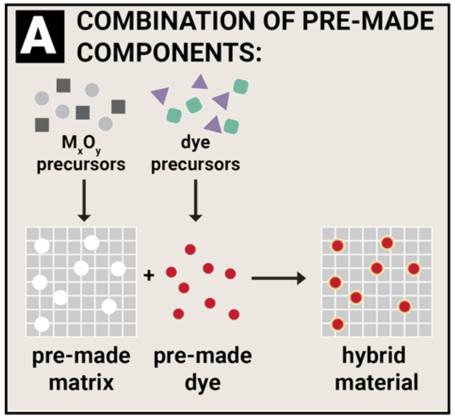

METHOD OF PREPARATION OF HMs	Entry	TYPE OF HMs	BONDING BETWEEN PBI AND SiO_2	SOLID STATE FLUORESCENCE (FL) BEHAVIOR	OTHER PHOTOLUMINESCENCE (PL) INFORMATION	APPLICATION [1]	REFERENCES
	1	PBI@SiO_2 films	non-covalent	not reported	-	-	S1
	2	PBI@chiral SiO_2	covalent	not reported	PL measured in dispersion in CHCl3, and shows solution-like FL	-	S2
	3	PBI@zeolite	non-covalent	solution-like FL	-	-	S3
	4	PBI@zeolite	non-covalent	not reported	PL measured in dispersion in toluene, and shows broad peaks	-	S4
	5	PBI+organic cation@zeolite	non-covalent	solution-like FL high QY *(measured as thin films)*	-	-	S5
	6	PBI@zeolite	non-covalent	solution-like FL high QY *(measured as thin films)*	-	-	S6
	7	PBI@MPS	non-covalent	not reported	-	-	S7
	8	PBI@MPS	non-covalent	solution-like FL	-	-	S8
	9	PBI@MPS	non-covalent	solution-like FL	-	-	S9
	10	PBI@zeolite	non-covalent	solution-like FL *(measured as thin films)*	-	-	S10
	11	PBI-doped gel glasses	non-covalent	solution-like FL	-	-	S11
	12	PBI@PS-SiO_2	non-covalent	not reported	PL measured in physiological solution, and shows solution-like FL	cell imaging	S12

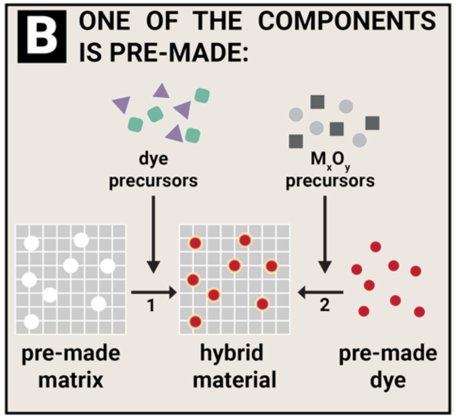

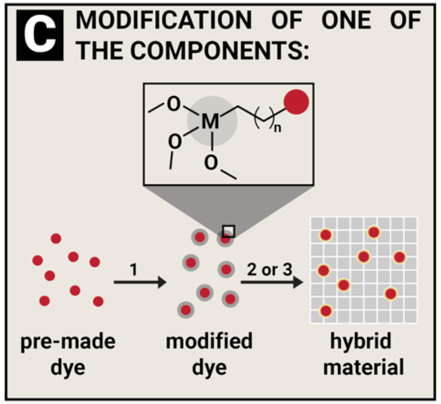

13	Core-shell PBI@SNP	non-covalent	solution-like FL *(measured as thin films)*	-	luminescent solar concentrators	S13
14	PBI-doped sol gel	non-covalent	not reported	-	pencil ink	S14
15	PBI@POSS	covalent	not reported	PL measured in solution in CHCl3	-	S15
16	PBI@POSS	covalent	broad peaks	-	-	S16
17	PBI@POSS	covalent	broad peaks	-	-	S17
18	PBI@SNP	covalent	solution-like FL	-	-	S18
19	PBI@MPS	covalent	broad peak	-	-	S19
20	Core-shell PBI@SNP	non-covalent	not reported	PL measured in dispersion in water or THF	cell imaging	S20
21	PBI@MPS	covalent	broad peak	-	-	S21
22	PBI@SiO_2	non-covalent	not reported	-	-	S22
23	PBI@g-C_3N_4@SiO_2	covalent	not reported	PL measured in suspension with acetonitrile	photocatalysis	S23
24	Core-shell PBI@SNP	covalent	not reported	PL measured in dispersion in ethanol	LED	S24
25	Core-shell PBI@SNP	covalent	not reported	PL measured in dispersion in ethanol or water	-	S25
26	PBI@SNP	covalent	not reported	PL measured in dispersion in ethanol	cell imaging	S26
27	PBI@SNP	covalent	not reported	PL measured in dispersion in THF	-	S27
28	PBI@SiO_2 films	covalent	reported for films, broad peaks	-	sensing aniline vapors	S28
29	PBI@SNP	covalent	not reported	PL measured in dispersion in THF or water	amine sensing	S29

**30**	PBI@SNP	covalent	not reported	PL measured in dispersion in DMF	photocatalysis	S30
**31**	PBI@SNP	covalent	not reported	PL measured in dispersion in $CHCl_3$ or water	-	S31
**32**	PBI@SiO_2 films	covalent	not reported	-	-	S32
**33**	PBI@SiO_2@LLDPE	covalent	Reported for polymeric films, show broad peak	-	-	S33
**34**	PBI@SNP	covalent	not reported	PL measured in dispersion in $CHCl_3$	-	S34
**35**	PBI@SiO_2	covalent	not reported	-	-	S35
**36**	PBI@SNP	covalent	not reported	PL measured in dispersion in water and/or methanol	cell imaging	S36
**37**	Magnetic core-shell PBI@SNP	covalent	not reported	PL measured in dispersion in water	cell imaging	S37
**38**	PBI@SNP	covalent	not reported	PL measured in dispersion in water, PE, or acetone	-	S38
**39**	PBI@SiO_2 xerogel	covalent	reported for thin films in quartz, broad peaks with presence of dye aggregates	-	-	S39
**40**	PBI@SNP	covalent	not reported	PL measured in dispersion in ethanol	-	S40
**41**	PBI@SiO_2	covalent	not reported	-	pigment	S41
**42**	PBI@super microporous SiO_2	non-covalent	not reported	-	-	S42
**43**	PBI@MPS	covalent	reported for thin film on glass subtract, broad peak		-	S43

**44**	PBI@MPS	covalent	not reported	PL reported in dispersion in ethanol	-	S44
**45**	PBI@PEO-MSNP	covalent	not reported	PL reported in dispersion	-	S45
**46**	PBI@MPS	non-covalent	broad peak	-	-	S46
**47**	PBI@PMO	covalent	not reported	-	-	S47
**48**	PBI@PEO-MPS	covalent	not reported	PL reported in dispersion in 1,4-dioxane	cell imaging	S48
**49**	PBI@MPS	covalent	broad peak	-	photocatalysis	S49
**50**	PBI@PEO-MPS	covalent	FL spectra not shown, but intensity is reported as high	-	imaging-guided chemotherapy	S50
**51**	PBI@Siloxane	covalent	solution-like FL, measured in ureasil probes	-	-	S51
**52**	PBI@SiO_2	covalent	measured as thin film, broad peak	-	-	S52

[1] application performed by the authors. Abbreviations: (PBI= perylene bisimide; PS= polystyrene; PEG= polyethylene glycol; PMO= periodic mesoporous organosilica; SNP= silica nanoparticle; MPS= mesoporous silica; MSNP= mesoporous silica nanoparticle). The different synthetic strategies towards organic-inorganic hybrid materials are illustrated in the frames A-C: (A) Strategies in which both organic and inorganic components are pre-made (e.g. dye loading into the matrix by gas/liquid sorption; mechanical mixing; ion exchange). (B) Strategies in which one of the components is pre-made and the second component is formed subsequently (1: the dye is synthesized inside the matrix ('ship-in-a-bottle') or 2: the matrix is made around the pre-made dye). (C) Strategies in which the organic component needs to be modified to be incorporated in the pre-made inorganic component (1: the pre-made dye is modified with an alkoxy-group; 2: sol-gel hybrid from hydrolysis of the modified-dye; 3: grafting of the modified-dye into the pre-made matrix).

2. Experimental Details

2.1 Chemicals

Perylene-3,4,9,10-tetracarboxylic dianhydride (PBA, 97 %, Sigma Aldrich), 1,7-Dibromoperylene-3,4,9,10-tetracarboxylic dianhydride (Br-PBA, 97%, ABCR), 1,6,7,12-Tetrachloro-perylene-3,4,9,10-tetracarboxylic acid dianhydride (Cl-PBA, 97%, ABCR), *n*-Propylamine (C3, 95%, Sigma Aldrich), *n*-Pentylamine (C5, 95%, Sigma Aldrich), Octylamine (C8, 98%, ABCR), Tetradecylamine (C14, 95%, Sigma Aldrich), Tetraethylorthosilicate (TEOS, 99%, Sigma Aldrich) and 3-Triethoxysilylpropylamine (APTS, 98%, Fluka) were used as received.

2.2 Synthesis

HT experiments were performed in Teflon-lined Parr Instruments general purpose acid digestion vessels with V= 45 mL. The experimental conditions are summarized in Table S2.

2.2.1 Synthesis of *n*-alkyl-PBI in the presence of TEOS. 1 equivalent of perylene-3,4,9,10-tetracarboxylic dianhydride (PBA, 0.4 mmol), 2 equivalents of the *n*-alkyl amine R-NH_2 (with R= C_3H_5 (C3), C_5H_9 (C5), C_8H_{15} (C8) and $C_{14}H_{27}$ (C14)) (0.8 mmol) and 15 mL distilled water were stirred in a PTFE vessel at RT for 20 min. To this dispersion, n equivalents (n= 1, 5, 10 and 100) of tetraethylorthosilicate (TEOS) were added dropwise and the mixture was stirred for another 5 min. The reaction mixture was then placed in a steel autoclave, and brought to an oven preheated at a reaction temperature (T_R) of 200 °C for a reaction time (t_R) of 24 hours. After t_R, the reaction was quenched by cooling the autoclave with cold tap water. The crude product was isolated via filtration and dried at 80 °C *in vacuo* overnight. The samples were named C*R*-PBI/SiO_2 where R is the number of carbons in the *n*-alkyl-chain of the employed amine. For comparison, pure dyes were prepared by the same procedure without the addition of TEOS and were named CR-PBI.

2.2.2 Synthesis of PBI@SiO_2 class II hybrid materials. 1 equivalent (0.4 mmol) of perylene-3,4,9,10-tetracarboxylic dianhydride (PBA) or 1,7-dibromoperylene-3,4,9,10-tetracarboxylic dianhydride (Br-PBA) or 1,6,7,12-tetrachloro-perylene-3,4,9,10-tetracarboxylic acid dianhydride (Cl-PBA), 2 equivalents (0.8 mmol) of aminopropyltriethoxysilane (APTS) and 15 mL distilled water were stirred at RT for 20 min inside of a PTFE vessel. The reaction mixture was placed in a steel autoclave and it was placed in an oven preheated to T_R = 200 °C, and kept there for t_R= 24h. After t_R, the reaction was quenched by cooling the autoclave with cold tap water. The crude product was isolated *via* filtration

and dried at 80 °C *in vacuo* overnight. The samples were named APTS-PBI@SiO_2, APTS-Br-PBI@SiO_2 and APTS-Cl-PBI@SiO_2.

2.2.3 Synthesis of APTS-Br-PBI@SiO_2 with different amounts of TEOS. 1 equivalent (0.4 mmol) of 1,7-dibromoperylene-3,4,9,10-tetracarboxylic dianhydride (Br-PBA), 2 equivalents (0.8 mmol) of aminopropyltriethoxysilane (APTS), *n* equivalents of tetraethylorthosilicate (TEOS) (*n*= 2, 20 and 100), and 15 mL distilled water were stirred in a PTFE vessel, at RT for 20 min. The PTFE vessel containing the reaction mixture was transferred to a steel autoclave and placed in an oven preheated to T_R = 200 °C, and kept there for t_R= 24h. After t_R, the reaction was quenched by cooling the autoclave with cold tap water. The crude product was isolated *via* filtration and dried at 80 °C *in vacuo* overnight. The samples were named APTS-Br-PBI@SiO_2 (*n* TEOS), where *n* is the equivalents of TEOS employed in the preparation.

Table S2. Summary of the materials prepared and their compositions.

ENTRY	NAME	PERYLENE	AMINE	APTS	TEOS	COMPOSITION[a]
1	C3-PBI	1 eq. PBA	2 eq. C3-NH_2	-	-	-
2	C3-PBI/SiO_2 (1 eq. TEOS)	1 eq. PBA	2 eq. C3-NH_2	-	1 eq.	75.93 wt% dye
3	C3-PBI/SiO_2 (5 eq. TEOS)	1 eq. PBA	2 eq. C3-NH_2	-	5 eq.	74.36 wt% dye
4	C3-PBI/SiO_2 (10 eq. TEOS)	1 eq. PBA	2 eq. C3-NH_2	-	10 eq.	75.16 wt% dye
5	C3-PBI/SiO_2 (100 eq. TEOS)	1 eq. PBA	2 eq. C3-NH_2	-	100 eq.	45.30 wt% dye
6	C3-Br-PBI	1 eq. Br-PBA	2 eq. C3-NH_2	.	-	-
7	pure SiO_2	-	-	-	1 eq.	-
8	APTS-PBI@SiO_2	1 eq. PBA	-	2 eq.	-	35.88 wt% dye (0.763 mmol dye/g HM)
9	APTS-Br-PBI@SiO_2	1 eq. Br-PBA	-	2 eq.	-	26.63 wt% dye (0.423 mmol dye/g HM)
10	APTS-Br-PBI@SiO_2 (2 TEOS)	1 eq. Br-PBA	-	2 eq.	2 eq.	25.40 wt% dye (0.403 mmol dye/g HM)
11	APTS-Br-PBI@SiO_2 (20 TEOS)	1 eq. Br-PBA	-	2 eq.	20 eq.	20.49 wt% dye (0.325 mmol dye/g HM)
12	APTS-Br-PBI@SiO_2 (100 TEOS)	1 eq. Br-PBA	-	2 eq.	100 eq.	8.11 wt% dye (0.129 mmol dye/g HM)
13	APTS-Cl-PBI@SiO_2	1 eq. Cl-PBA	-	2 eq.	-	-

[a] composition of the hybrid materials was determined by TGA.
The materials C5-PBI/SiO_2, C8-PBI/SiO_2 and C14-PBI/SiO_2 consist of a mere mixture of dye and silica particles, and no further analysis was performed.

2.3 Characterizations

Powder X-ray diffraction (PXRD) data was collected with a PANalytical X'Pert Pro multipurpose diffractometer (MPD) in Bragg Brentano geometry operating with a Cu anode at 40 kV, 40 mA. An X-Celerator multichannel detector was used. The diffraction patterns were recorded between 5 ° and 60 ° (2θ) with 69.215 s/step and a step size of 0.0050134 °. Sample holders were rotated during the measurement with 4 s/turn. Samples were ground and mounted as loose powders on silicon single crystal sample holders. Small-angle X-ray (SAXS) measurements were performed with Cu-K_α radiation (λ =0.1542 nm) from a Bruker Nanostar, equipped with a pinhole camera and a 2D detector (VÅNTEC 2000). Samples were placed between two polymeric foils and measured in vacuum. All patterns were azimuthally integrated and background corrected to obtain the scattering intensity in dependence on the scattering vector q, being defined as $q=(4\pi/\lambda)\sin\theta$, with 2θ being the scattering angle. The sample to detector distance was chosen at 28 cm, which gives an accessible q-range from 0.3 to 10.6 nm^{-1}, corresponding to a size from 0.6 to nearly 21 nm in real space.

Attenuated total reflectance Fourier transform infrared (ATR-FTIR) spectra were recorded on a PerkinElmer UATR Two with a diamond crystal. Resolution was set to 4 cm^{-1} and spectra were recorded from 4000 to 450 cm^{-1}. ^{13}C and ^{29}Si solid state NMR spectra were recorded on a Bruker AVANCE 300 (^{13}C at 75.4 MHz and ^{29}Si at 59.57 MHz) equipped with a 4 mm broadband MAS probe head. Spectra were recorded with ramped CP-MAS (cross polarization magic angle spinning) experiments. The sample holders were spun at 10 kHz for both ^{29}Si and ^{13}C.

Thermogravimetric analysis (TGA) experiments were performed with a PerkinElmer TGA8000 and TA Instruments TGA Q500, under nitrogen flow using a heating rate of 10 °C/min, from rt to 1000 °C.

Scanning electron microscopy (SEM) was carried out with a Quanta 200F FEI microscope. Typically, the samples were measured at 5 kV with a working distance of *ca.* 9 mm and spot size 2.5. Prior to imaging, samples were coated by sputtering a 17 nm thick layer of Au/Pd 60/40 alloy with a Quarum Q105T S sample preparation system.

The APTS-PBI sample was deposited on carbon film copper grids for transmission electron microscope (TEM) characterization. A FEI TECNAI F20 operated at 200 kV and equipped with EDX (EDAX Apollo XLTW SDD) detector was used.

UV/VIS absorption spectra were recorded in solid state or $CHCl_3$ solutions (1 μM) with a Perkin Elmer Lambda 750 spectrometer. Fluorescence emission and excitation of the powders were measured on a PicoQuant FluoTime300 spectrometer, equipped with Xe lamp and single monochromator. Spectra were collected with step size of 2 nm, integration time of 1 s, and a 525 nm filter was employed to reduce the stray light effect.

The GC-MS spectra were collected in a ThermoScientific equipment (model Trace 1300 GC) with BGB5 column, and coupled to a single quadrupole mass spectrometer (ThermoScientific ISQ LT).

3. Results

3.1. The simultaneous synthesis of *n*-alkyl-PBI and SiO_2

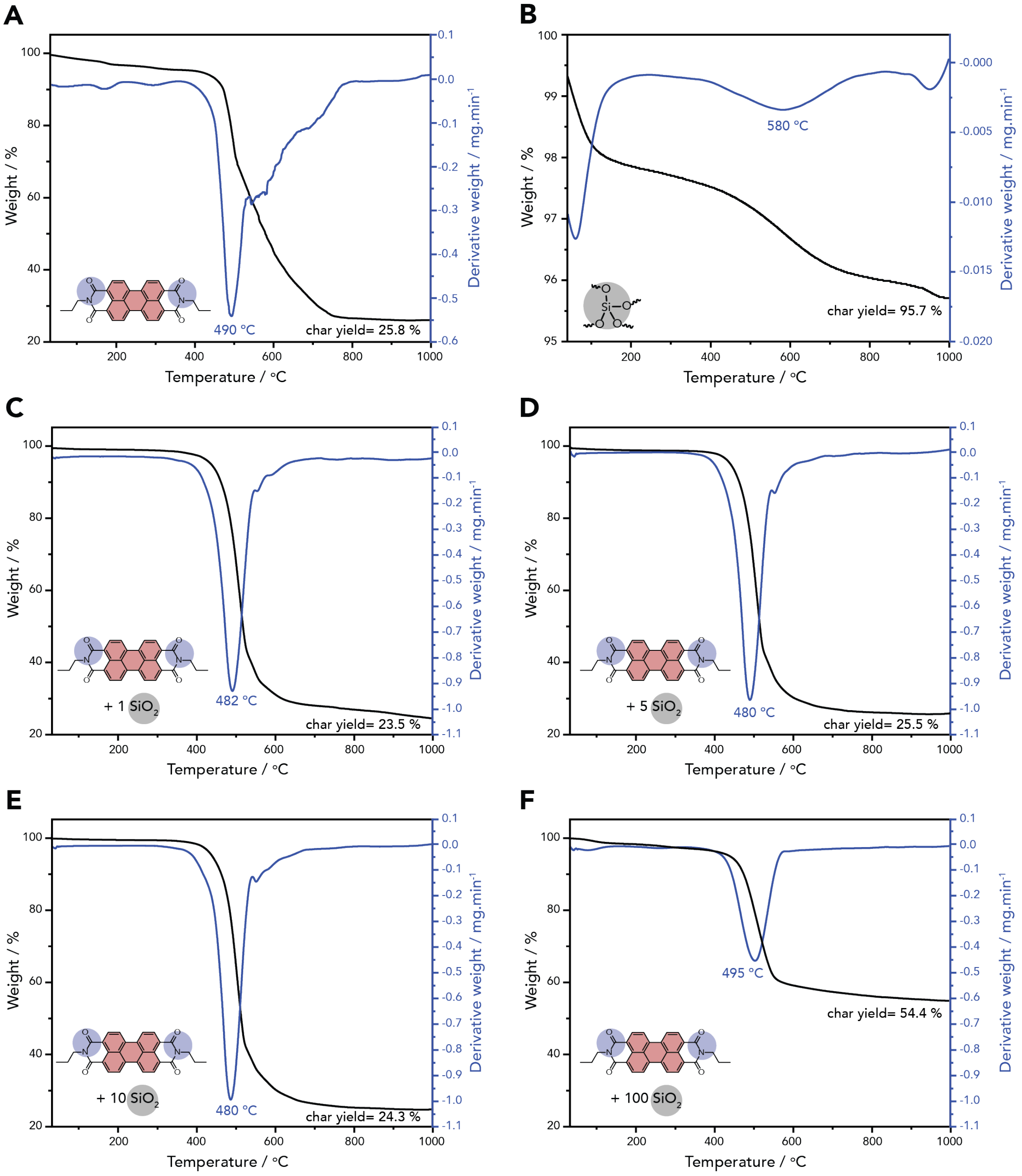

Figure S1. TGA of C3-PBI, SiO_2 and PBI-SiO_2 synthesized without a linker. Shown are the pure C3-PBI dye (**A**), pure SiO_2 (**B**), C3-PBI/SiO_2 (1 eq. TEOS) (**C**), C3-PBI/SiO_2 (5 eq. TEOS) (**D**), C3-PBI/SiO_2 (10 eq. TEOS) (**E**) and C3-PBI/SiO_2 (100 eq. TEOS) (**F**).

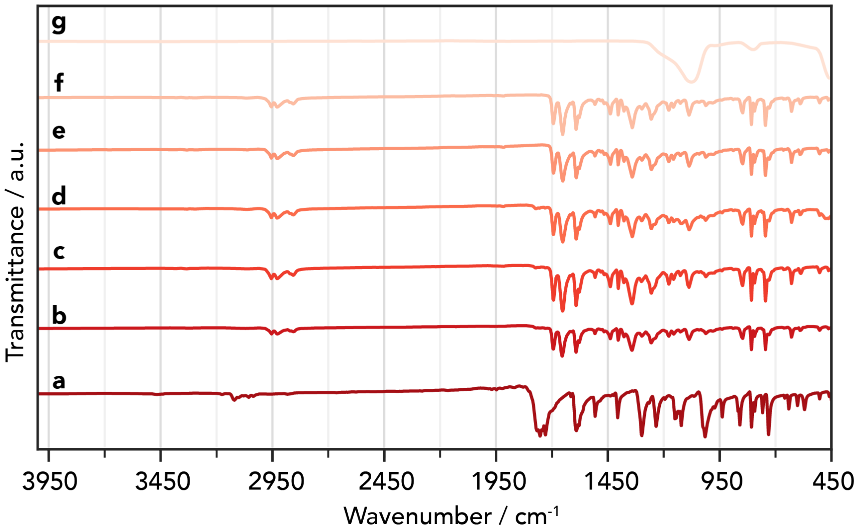

Figure S2. FTIR spectra of a) starting compound PBA, b) pure C5-PBI dye, c) C5-PBI@SiO_2 (1 eq. TEOS), d) C5-PBI@SiO_2 (5 eq. TEOS), e) C5-PBI@SiO_2 (10 eq. TEOS), f) C5-PBI@SiO_2 (100 eq. TEOS) and g) pure SiO_2.

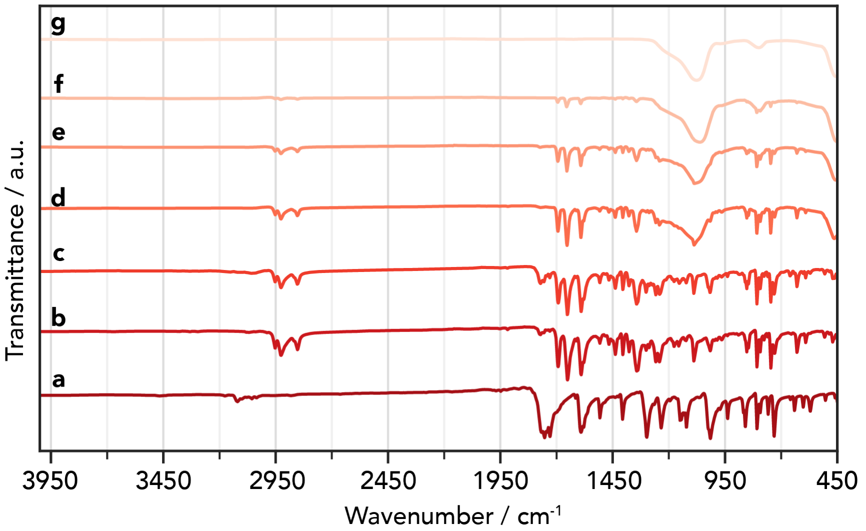

Figure S3. FTIR spectra of a) starting compound PBA, b) pure C8-PBI dye, c) C8-PBI@SiO_2 (1 eq. TEOS), d) C8-PBI@SiO_2 (5 eq. TEOS), e) C8-PBI@SiO_2 (10 eq. TEOS), f) C8-PBI@SiO_2 (100 eq. TEOS) and g) pure SiO_2.

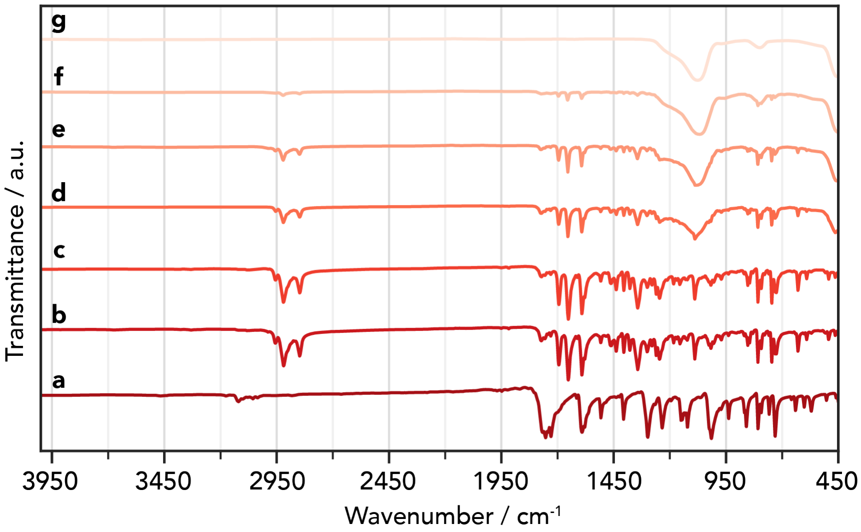

Figure S4. FTIR spectra of a) starting compound PBA, b) pure C14-PBI dye, c) C14-PBI@SiO_2 (1 eq. TEOS), d) C14-PBI@SiO_2 (5 eq. TEOS), e) C14-PBI@SiO_2 (10 eq. TEOS), f) C14-PBI@SiO_2 (100 eq. TEOS) and g) pure SiO_2.

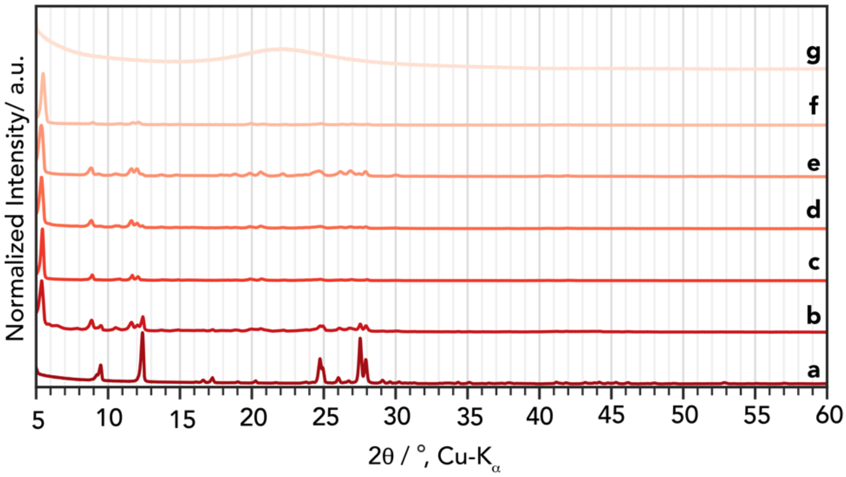

Figure S5. PXRD patterns of a) starting compound PBA, b) pure C5-PBI dye, c) C5-PBI@SiO_2 (1 eq. TEOS), d) C5-PBI@SiO_2 (5 eq. TEOS), e) C5-PBI@SiO_2 (10 eq. TEOS), f) C5-PBI@SiO_2 (100 eq. TEOS) and g) pure SiO_2.

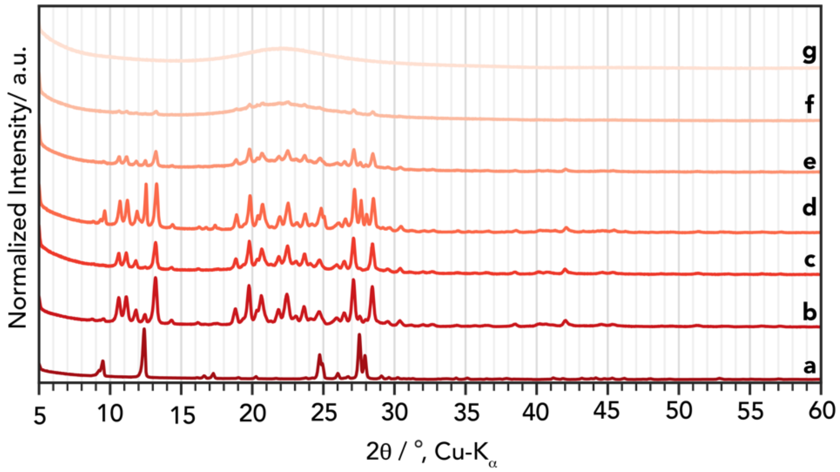

Figure S6. PXRD patterns of a) starting compound PBA, b) pure C8-PBI dye, c) C8-PBI@SiO_2 (1 eq. TEOS), d) C8-PBI@SiO_2 (5 eq. TEOS), e) C8-PBI@SiO_2 (10 eq. TEOS), f) C8-PBI@SiO_2 (100 eq. TEOS) and g) pure SiO_2.

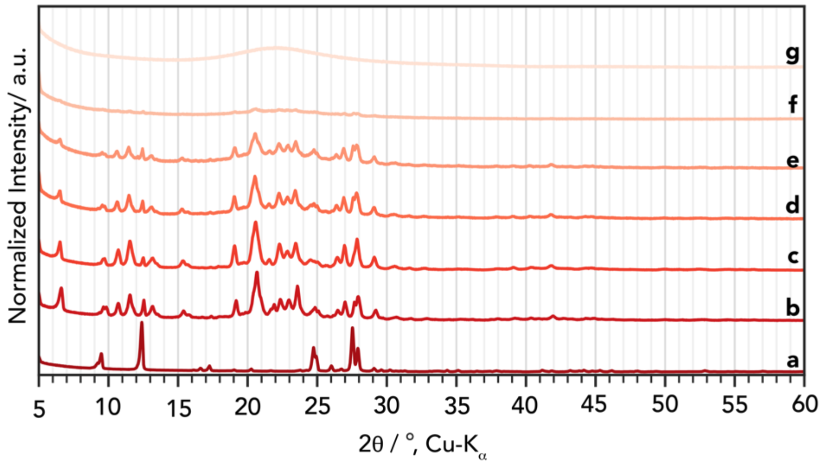

Figure S7. PXRD patterns of a) starting compound PBA, b) pure C14-PBI dye, c) C14-PBI@SiO_2 (1 eq. TEOS), d) C14-PBI@SiO_2 (5 eq. TEOS), e) C14-PBI@SiO_2 (10 eq. TEOS), f) C14-PBI@SiO_2 (100 eq. TEOS) and g) pure SiO_2.

3.2. HTS of Class II *n*-alkyl-PBI@SiO_2 hybrid materials

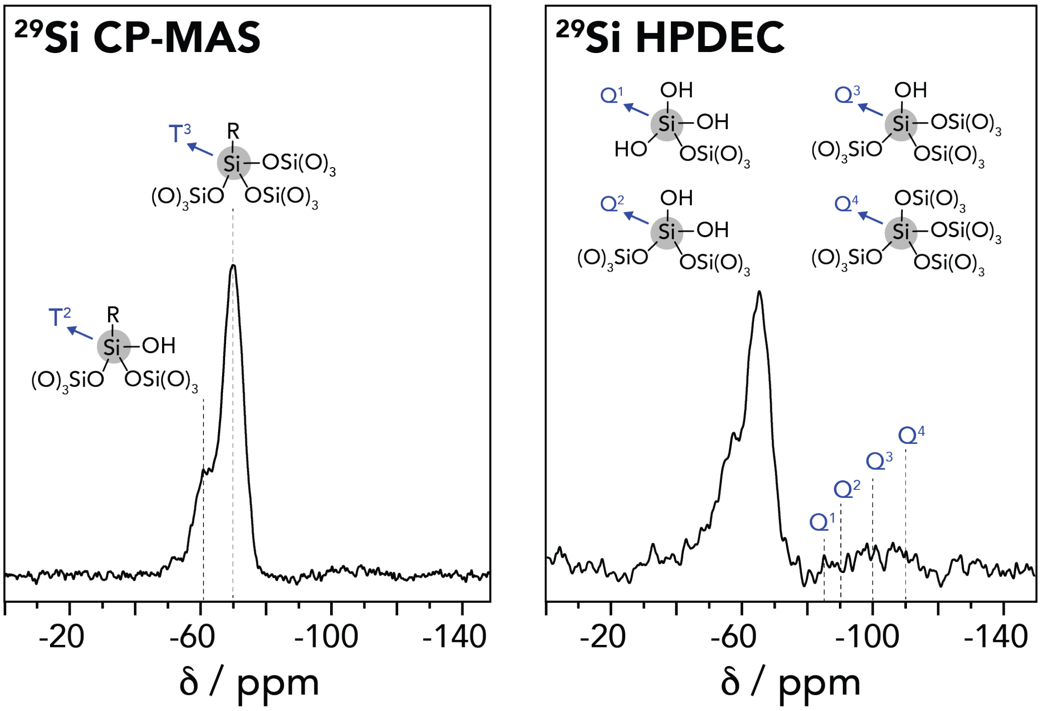

Figure S8. Solid state CPMAS and HPDEC ^{29}Si NMR spectra of APTS-PBI@SiO_2.

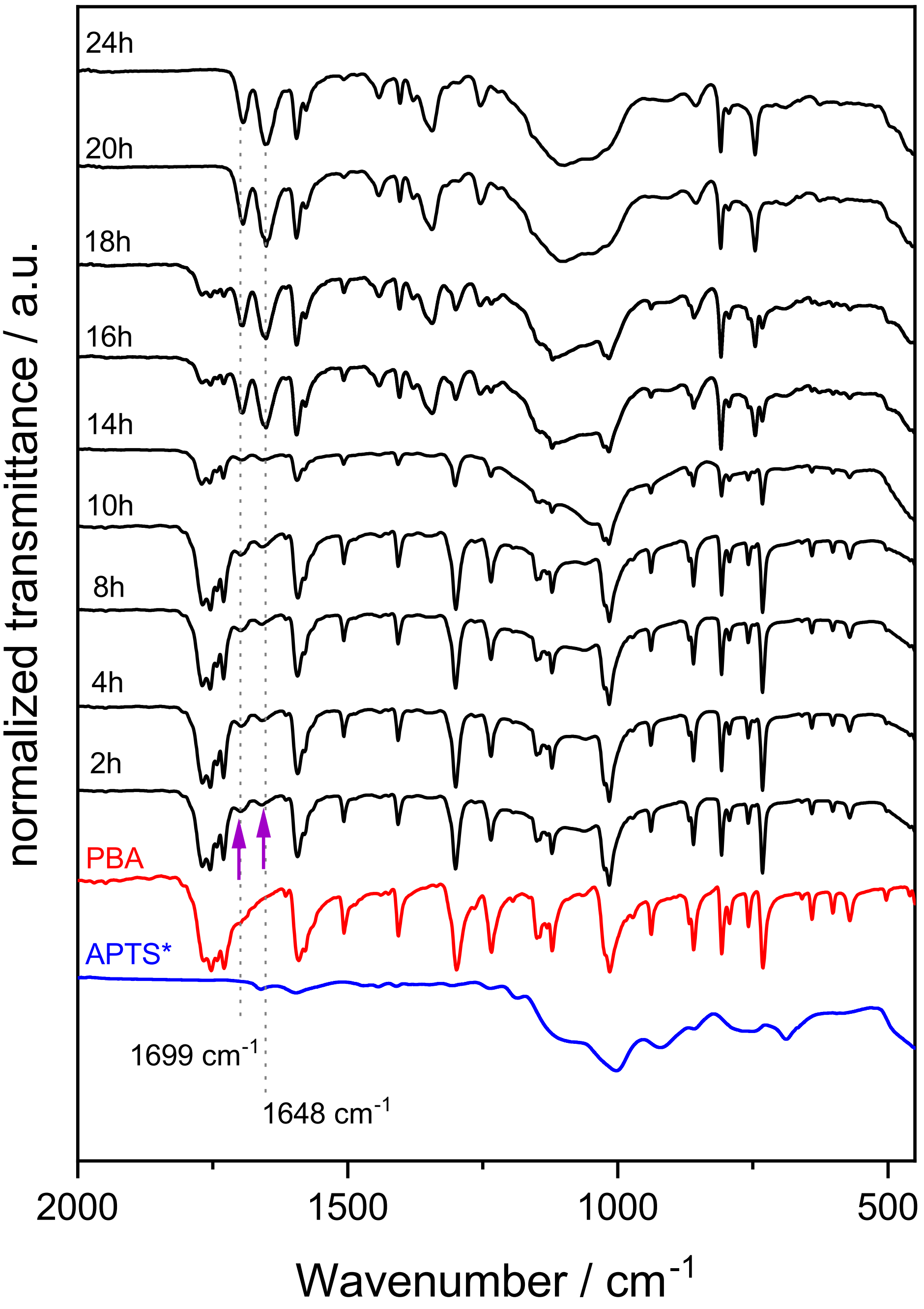

Figure S9. ATR-FTIR of the APTS-PBI@SiO_2 at different times of reaction. The starting compound PBA is shown in red and its reaction with APTS after 2h to 24h of reaction under HTS in black. In 2h of reaction, the CO modes of bisimides are observed at 1699 and 1648 cm^{-1}. APTS* was heated at 200 °C for 2h under HTS for comparison (in blue).

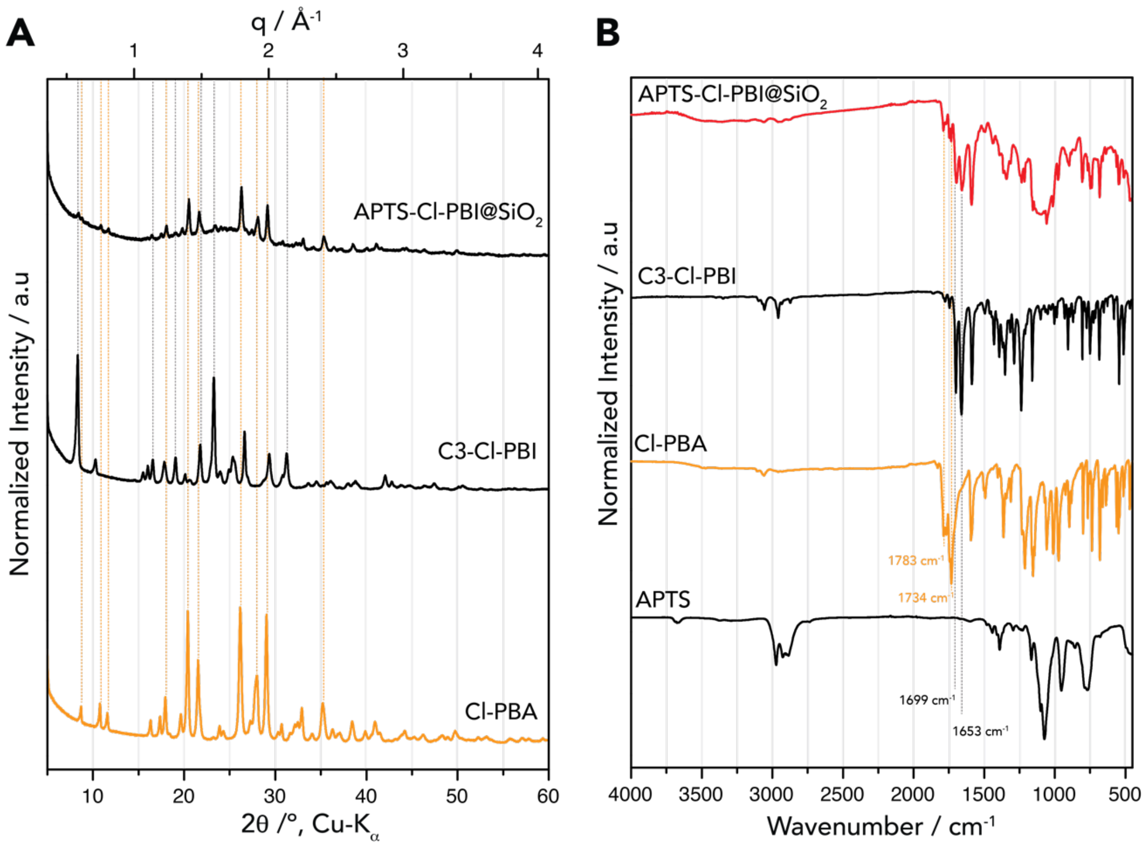

Figure S10. PXRD (A) and ATR-FTIR (B) of APTS-Cl-PBI@SiO_2.

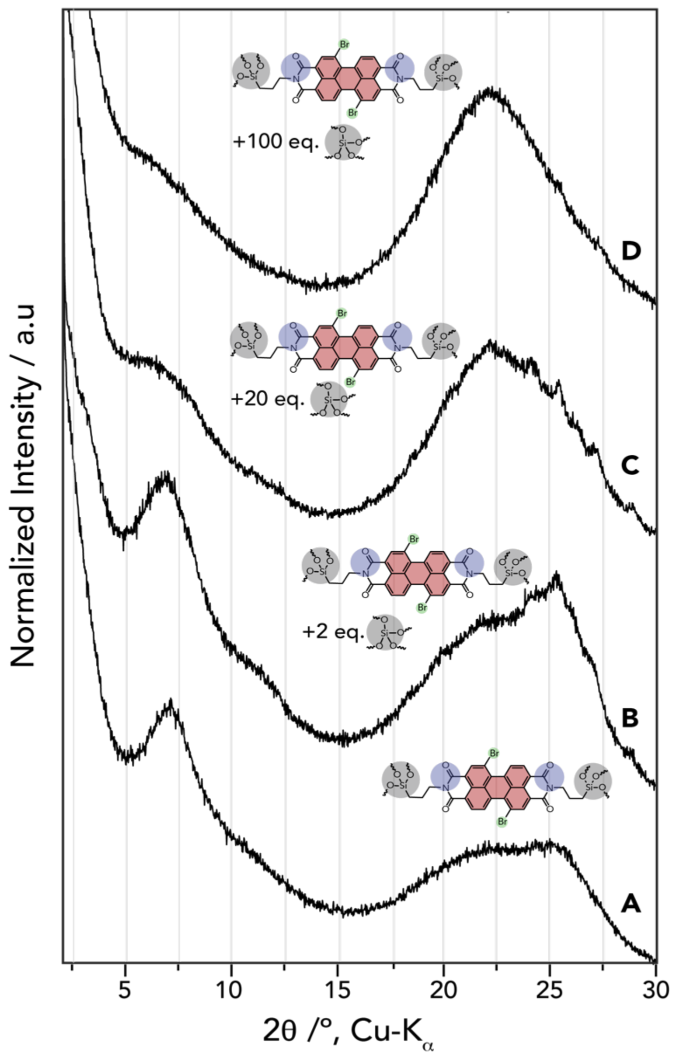

Figure S11. PXRD of APTS-Br-PBI@SiO_2 (2 APTS: 1 Br-PBA) (**A**), APTS-Br-PBI@SiO_2 (2 APTS: 2 TEOS: 1 Br-PBA) (**B**), APTS-Br-PBI@SiO_2 (2 APTS: 20 TEOS: 1 Br-PBA) (**C**) and APTS-Br-PBI@SiO_2 (2 APTS: 100 TEOS: 1 Br-PBA) (**D**).

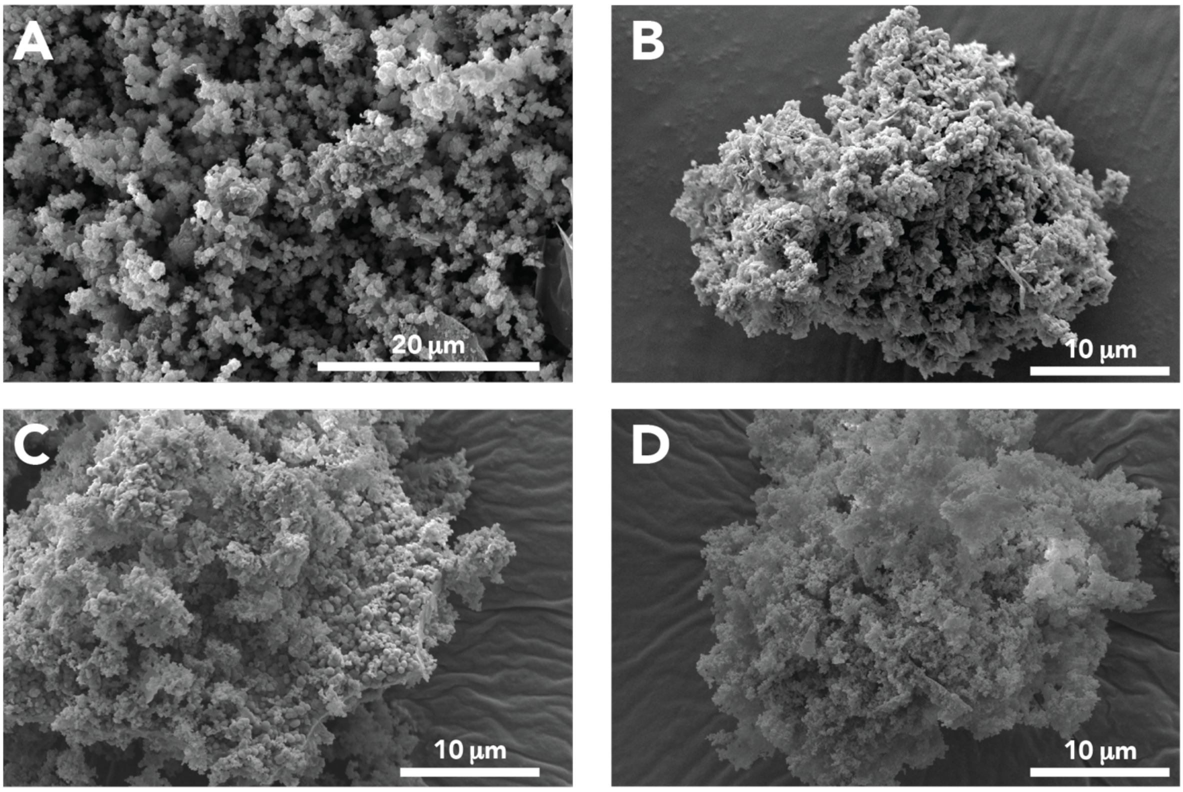

Figure S12. SEM of APTS-Br-PBI@SiO_2 (2 APTS: 1 Br-PBA) (**A**), APTS-Br-PBI@SiO_2 (2 APTS: 2 TEOS: 1 Br-PBA) (**B**), APTS-Br-PBI@SiO_2 (2 APTS: 20 TEOS: 1 Br-PBA) (**C**) and APTS-Br-PBI@SiO_2 (2 APTS: 100 TEOS: 1 Br-PBA) (**D**).

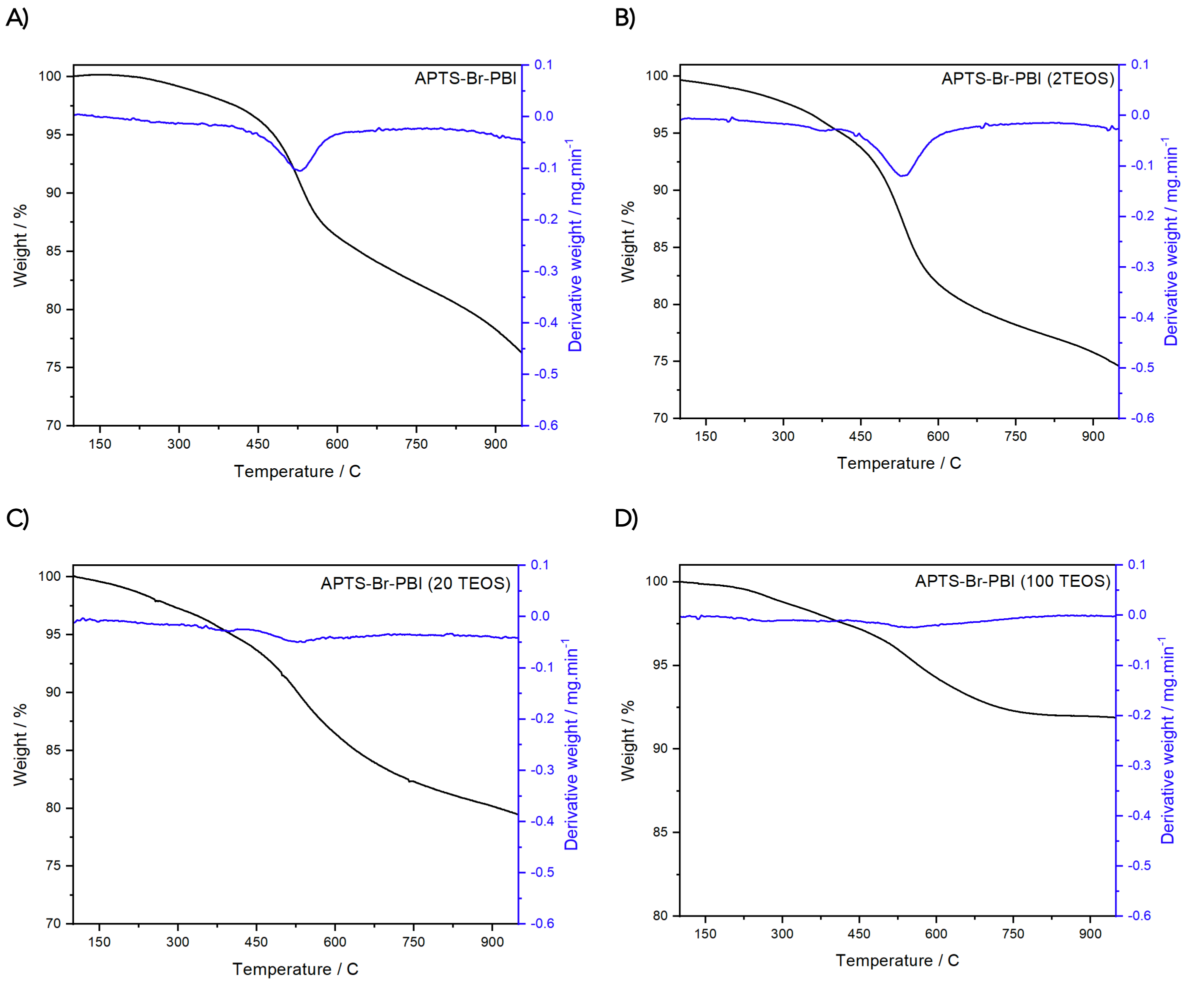

Figure S13. TGA of APTS-Br-PBI@SiO_2 (2 APTS: 1 Br-PBA) (**A**), APTS-Br-PBI@SiO_2 (2 APTS: 2 TEOS: 1 Br-PBA) (**B**), APTS-Br-PBI@SiO_2 (2 APTS: 20 TEOS: 1 Br-PBA) (**C**) and APTS-Br-PBI@SiO_2 (2 APTS: 100 TEOS: 1 Br-PBA) (**D**).

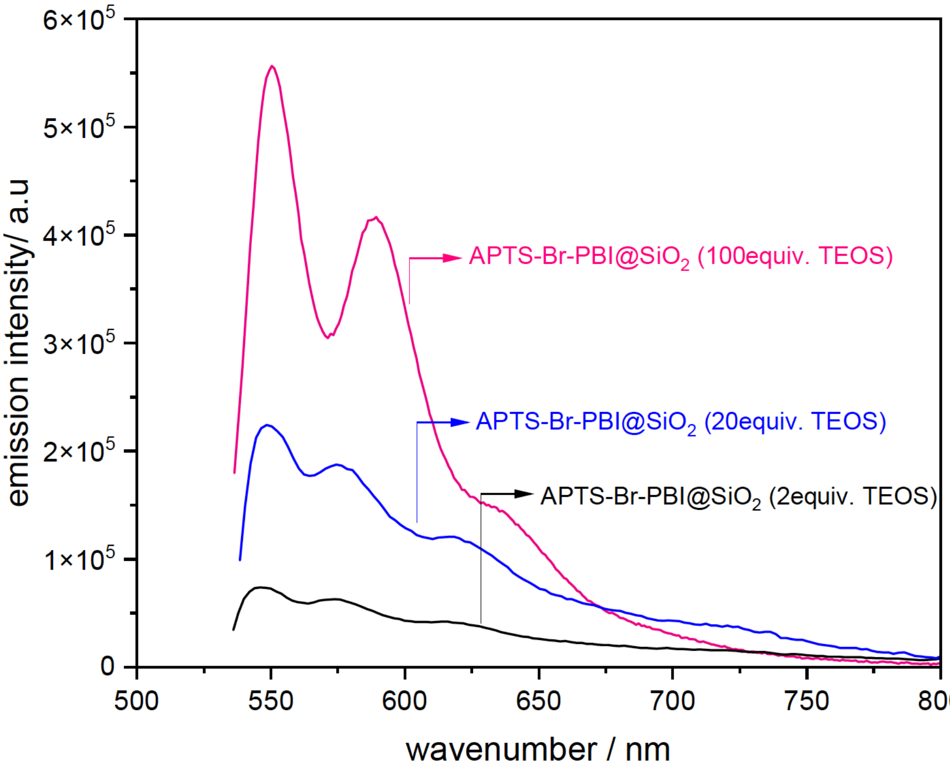

Figure S14. Non-normalized emission spectra of Class II hybrids APTS-Br-PBI@SiO_2 (2-100TEOS).

3.3. Quantum yield (ϕ)

The ϕ of the hybrid APTS-Br-PBI@SiO_2 (100TEOS) was determined by direct method, using an integrating sphere. First, to measure the scattering of the incident light, a spectrum of an empty quartz cuvette was collected (S_0, blue curve in Fig. S16). The sample was then placed into the cuvette in the integrating sphere, and a spectrum was collected (S_1, pink curve in Fig. S16). In both measurements, the excitation wavelength was set to 430 nm, and the spectra were collected from 420-440 nm with step size of 0.1 nm, and integration time of 0.5 s. The emission spectrum of the sample was then collected from 445 nm to 700 nm, with step size of 0.1 nm, and integration time of 0.5 s, and it is depicted in green in Fig. S16.
The QY is defined by the sum of all emitted photons (S_2), divided by the sum of all absorbed photos (S_0-S_1):

$$\Phi = \frac{S_2}{S_0 - S_1} x100$$

The corresponding areas of the peaks depicted in Fig S16 were calculated in Origin, by integrating the area below the curves, and were found as S_0= 5.99x10^7, S_1= 3.56x10^{7}and S_2= 1.72x10^5. Thus, the ϕ value was determined as 0.7%:

$$\Phi = \frac{1.72x10^5}{5.99x10^7 - 3.56x10^7} x100 = 0.7\%$$

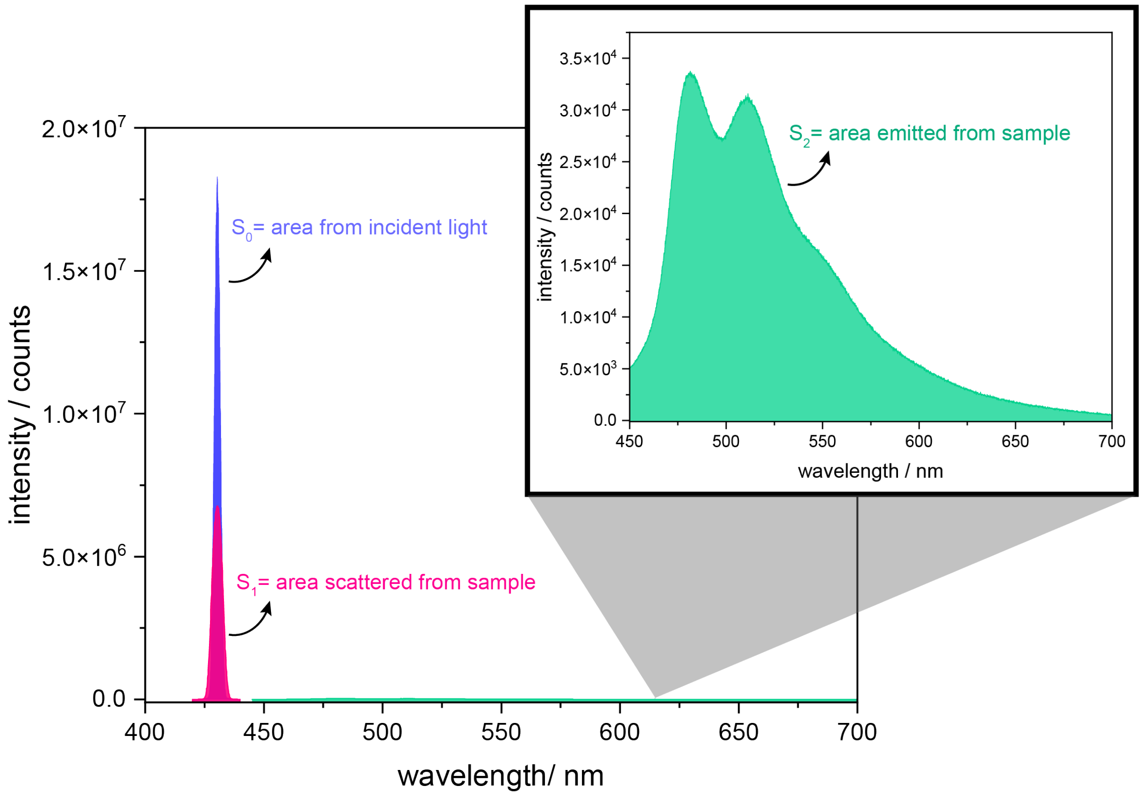

Figure S15. Quantum yield (QY) determination of APTS-Br-PBI@SiO_2 (100TEOS). Spectra of the excitation scatter region of the sample (S_1, highlighted in pink) and the blank (S_0, in blue), and spectrum of the emission region of the sample (S_2, in green).

3.4 Band gap calculation

The energy band gap of the hybrids and their individual components – all made by HTS – were estimated from the solid state UV-Vis spectroscopy, using Tauc's relationship $(\alpha h\nu)^{1/n} = A\ (h\nu - E_g)$ where A is a proportionality constant, 'hν' is the photon energy, 'α' the absorption coefficient and 'E_g' is the energy band gap of the material. 'n' is a constant whose value depends upon the type of transition, n = 1/2 for direct allowed transition and 2 for indirect allowed transition.[53] Figure S15 display the graphs of $(\alpha h\nu)^{1/n}$ versus hν for n = 1/2 and the corresponding value of direct energy band gap was calculated by drawing a tangent to the point of inflection on $(\alpha h\nu)^2$ versus hν curve. The extrapolation of the tangent line to the hν axis gives the value of energy band gap (E_g) in each case.

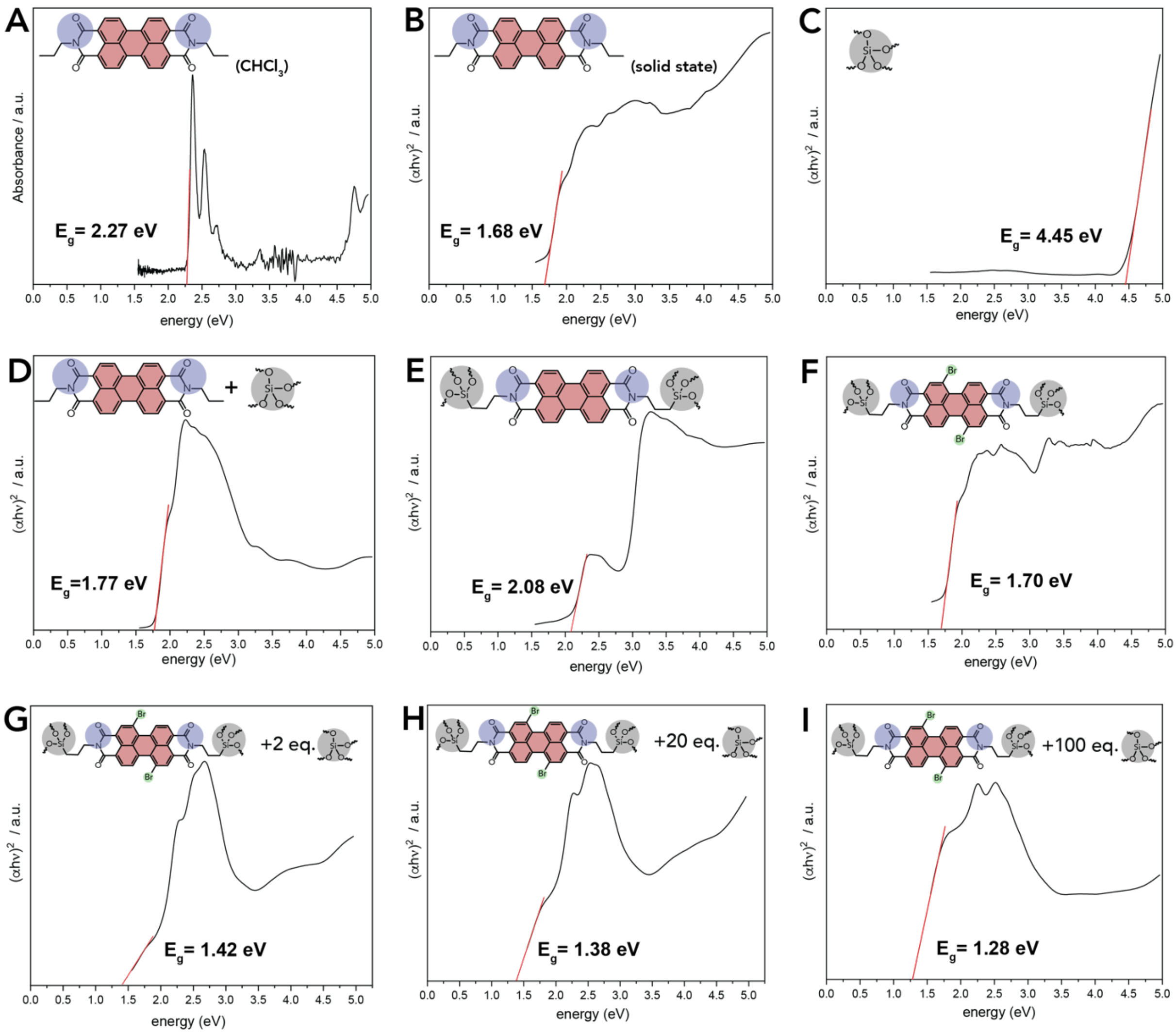

Figure S16. Determination of E_g of the hybrid materials made by HTS. (**A**) C3-PBI in $CHCl_3$; (**B**) C3-PBI in the solid state; (**C**) pure SiO_2; (**D**) mixture C3-PBI/SiO_2 (100 TEOS); (**E**) APTS-PBI@SiO_2; (**F**) APTS-Br-PBI@SiO_2; (**G**) APTS-Br-PBI@SiO_2 (2 TEOS); (**H**) APTS-Br-PBI@SiO_2 (20 TEOS); (**I**) APTS-Br-PBI@SiO_2 (100 TEOS).

3.5. Photocatalysis

In a typical procedure, reaction dispersions were prepared by adding 77.5 mg of the catalyst **APTS-Br-PBI@SiO_2 (100 TEOS)** (i.e. the equivalent of 0.010 mmol of the organic C3-Br-PBI moiety) to DMF (10 mL) in a Schlenk flask. Next, 0.1 mmol of iodo-benzaldehyde (IBZ) and 0.80 mmol of triethylamine (TEA) were added to the catalyst dispersion. The flask was purged with argon gas for 30 min to remove oxygen and protected under argon atmosphere during irradiation. During the photoreaction process, the reactor was kept at 30-32 °C with the aid of a ventilator.

The reaction mixture was irradiated through the side of the Schlenk flask using a 455 nm LED lighting (Kessil®, PR160L). After 8h of reaction, the photocatalyst particles was collected by centrifugation, dried under vacuum and further characterized. The remaining filtrate were then analyzed by GC-MS. The procedure was performed in triplicate. Photoreduction conversions were calculated from GC measurements using a calibration curve of the standard compounds.

For comparison purposes, the procedure was also performed using (i) the homogeneous catalyst C3-Br-PBI instead of the HM; (ii) pure SiO_2; (iii) with HM photocatalyst but without light; and (iv) without any catalyst.

In order to investigate the recyclability of the photocatalyst, the above-described procedure was repeated twice using the recovered catalyst. After 8h of reaction, the catalyst was recovered by centrifugation, dried under vacuum, and characterized by PXR, ATR-FTIR, TGA and SEM (Fig. S17).

3.5.1 Characterization of the HM after photocatalysis

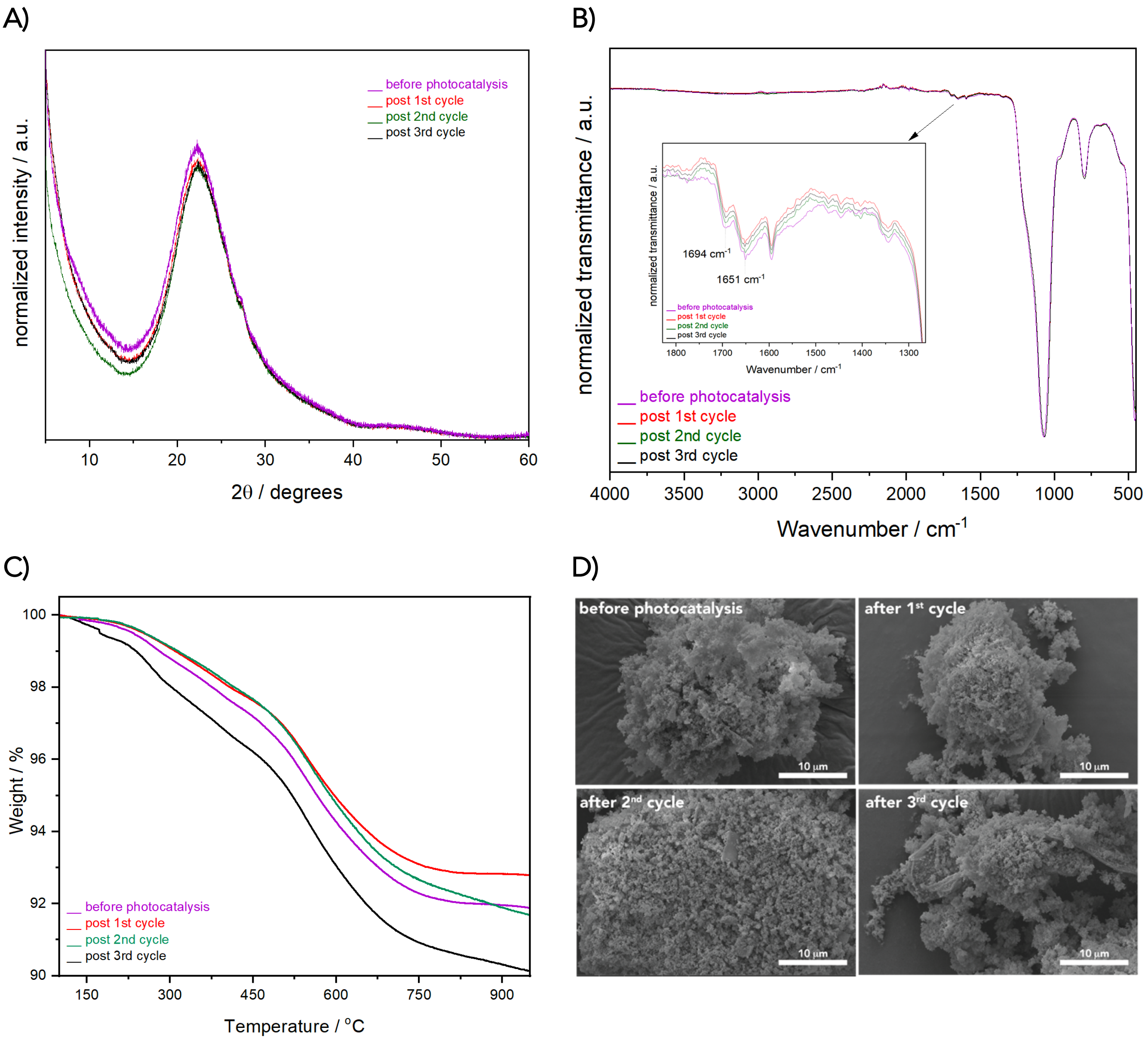

Figure S17. APTS-Br-PBI@SiO_2 (100 TEOS) before and after photoreduction of 3-iodobenzaldehyde under visible light (λ= 455 nm). (**A**) PXRD, (**B**) ATR-FTIR, (**C**) TGA, and (**D**) SEM microscopy.

3.5.2 GC-MS of the products of the photoreduction reactions

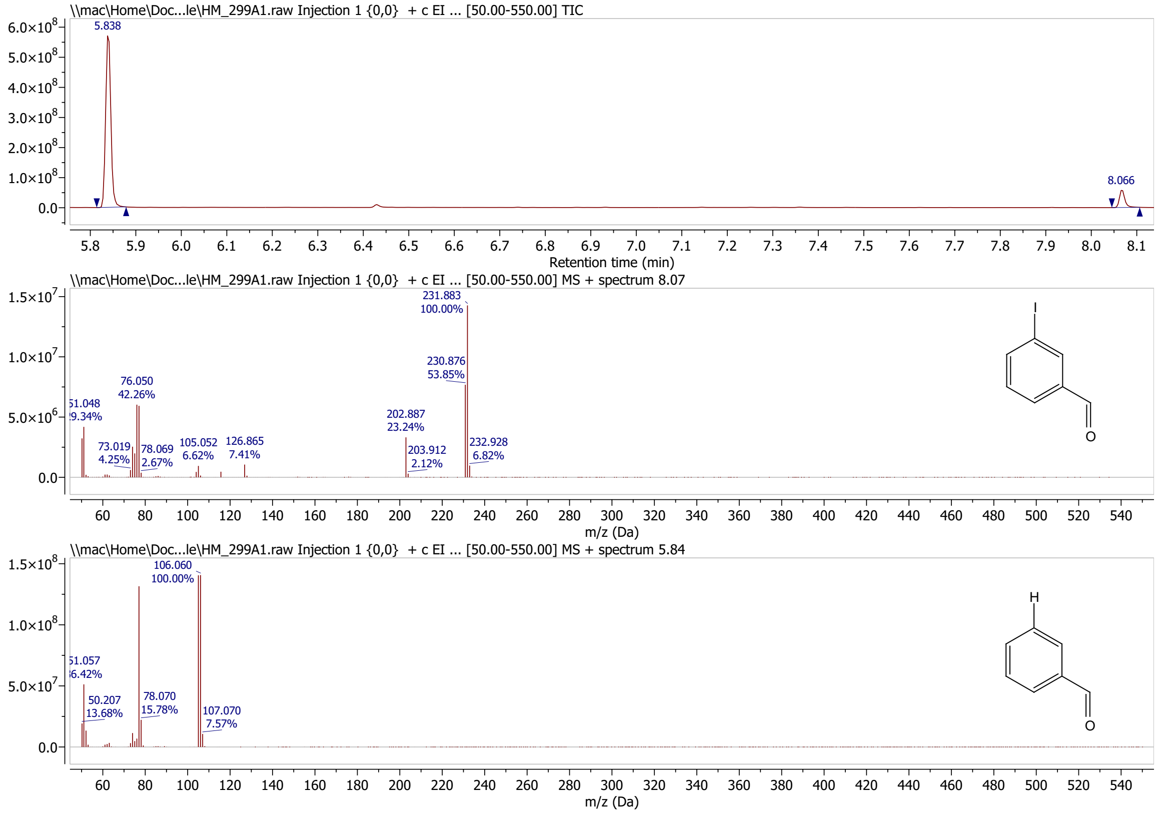

Figure S18. GC-MS analysis of the filtrate from the first cycle of photoreduction reaction with APTS-Br-PBI@SiO_2 (100 TEOS). On top, the gas chromatogram is shown and the main peaks highlighted: the retention time of iodobenzaldehyde was found at ~8.1 min and benzaldehyde at ~5.8 min. In the middle and bottom parts, are the MS spectra of benzaldehyde and iodobenzaldehyde, respectively.

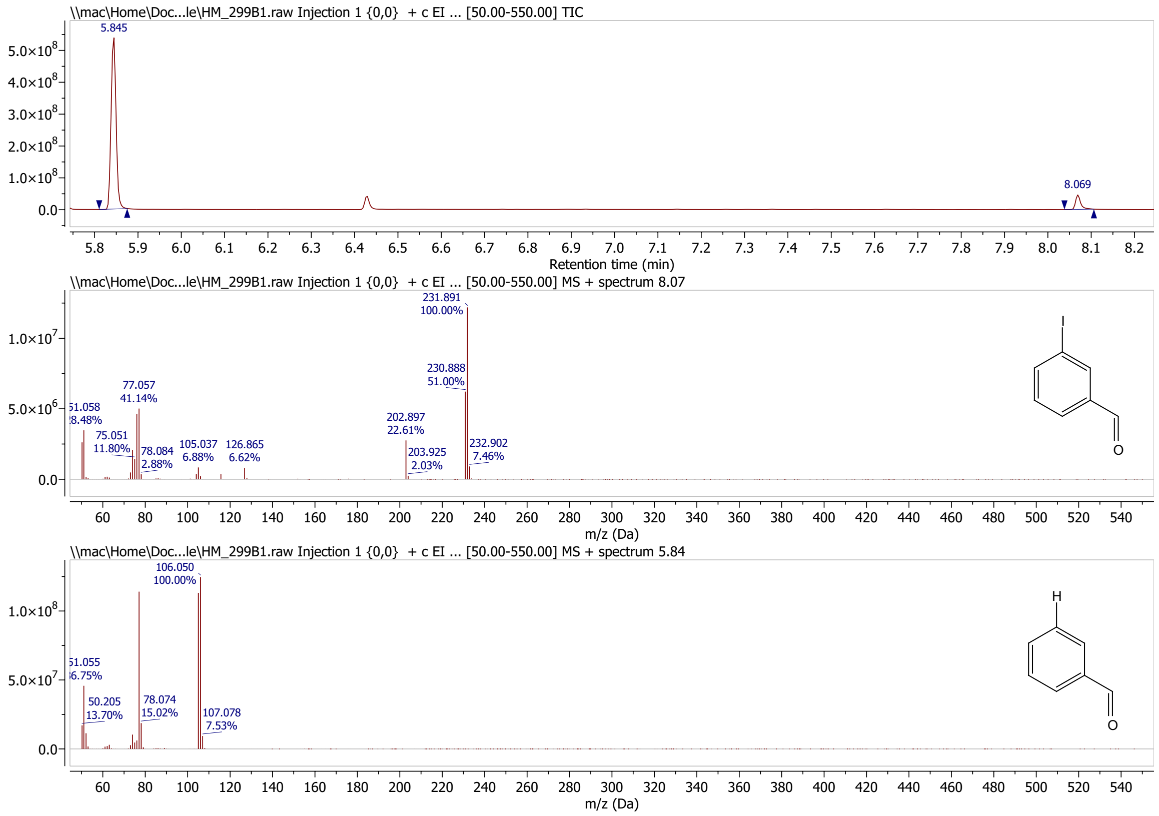

Figure S19. GC-MS analysis of the filtrate from the second cycle of photoreduction reaction with APTS-Br-PBI@SiO_2 (100 TEOS). On top, the gas chromatogram is shown and the main peaks highlighted: the retention time of iodobenzaldehyde was found at ~8.1 min and benzaldehyde at ~5.8 min. In the middle and bottom parts, are the MS spectra of iodobenzaldehyde and benzaldehyde, respectively.

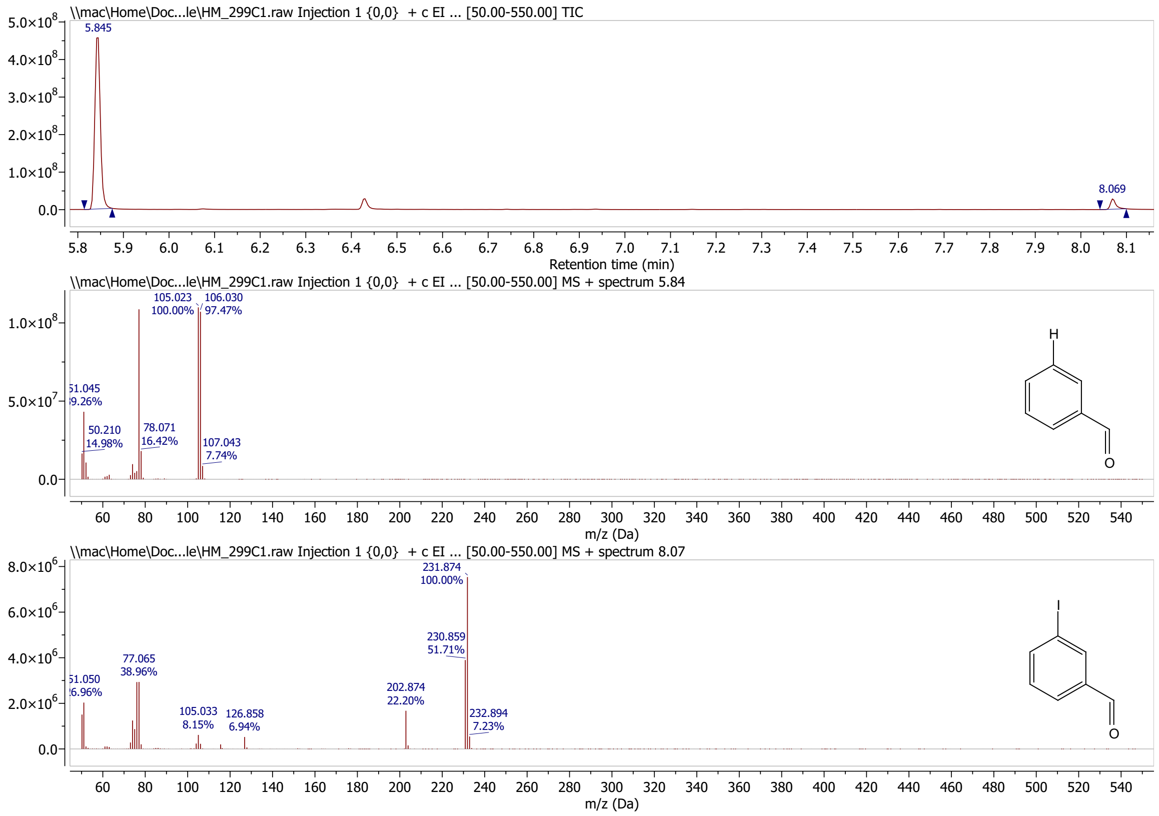

Figure S20. GC-MS analysis of the filtrate from the third cycle of photoreduction reaction with APTS-Br-PBI@SiO_2 (100 TEOS). On top, the gas chromatogram is shown and the main peaks highlighted: the retention time of iodobenzaldehyde was found at ~8.1 min and benzaldehyde at ~5.8 min. In the middle and bottom parts, are the MS spectra of iodobenzaldehyde and benzaldehyde, respectively.

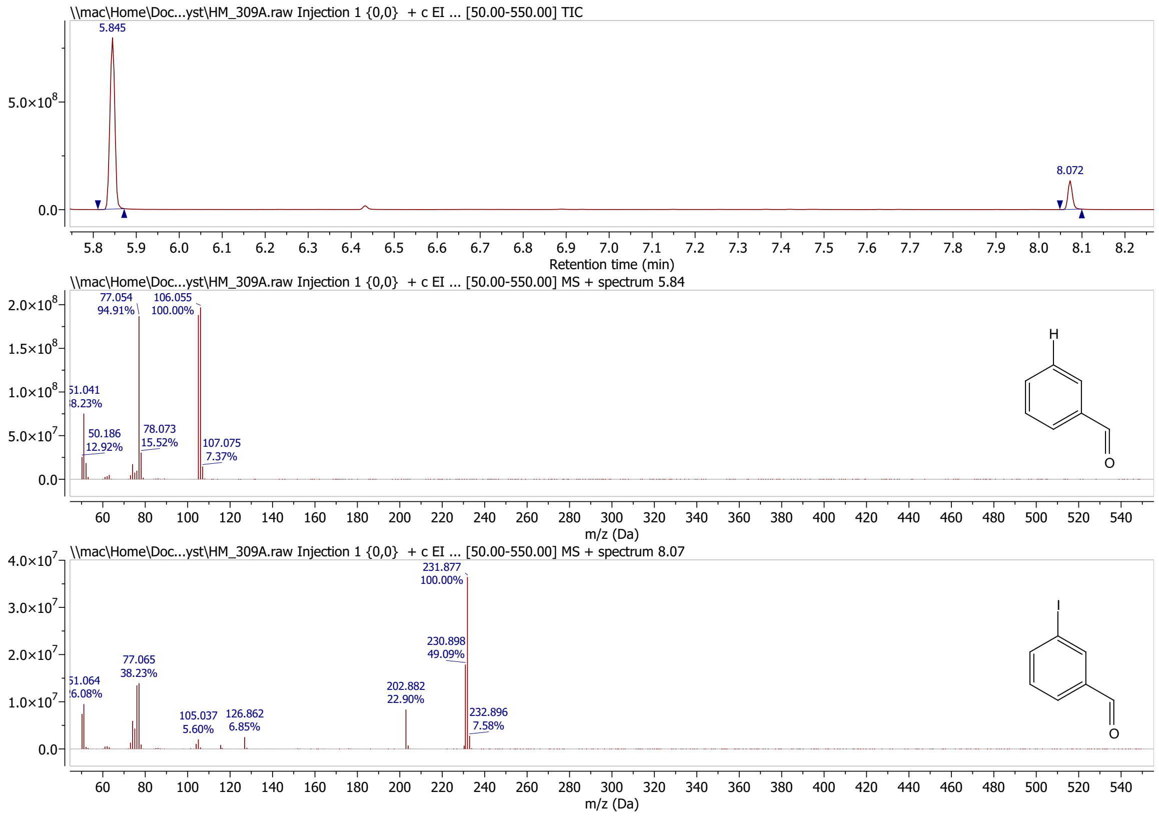

Figure S21. GC-MS analysis of the filtrate from the photoreduction reaction with C3-Br-PBI (homogeneous catalyst). On top, the gas chromatogram is shown and the main peaks highlighted: the retention time of iodobenzaldehyde was found at ~8.1 min and benzaldehyde at ~5.8 min. In the middle and bottom parts, are shown the MS spectra of benzaldehyde and iodobenzaldehyde, respectively.

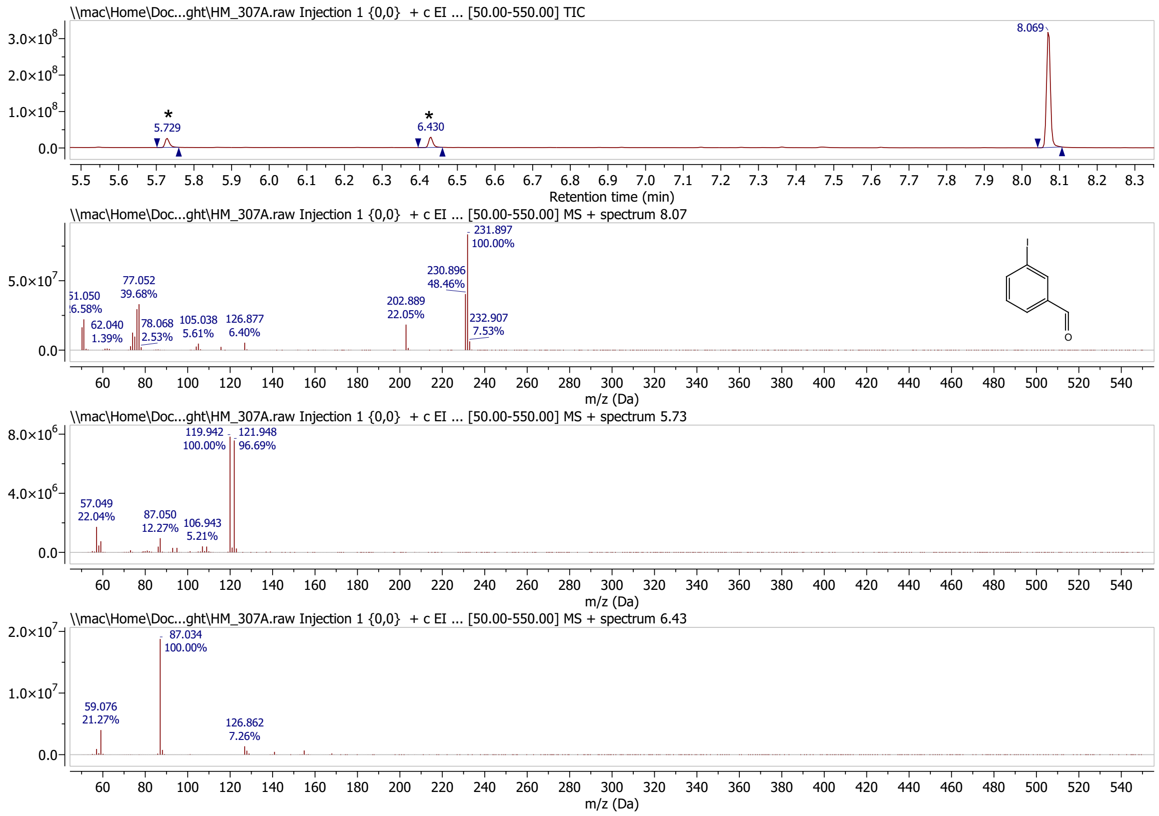

Figure S22. GC-MS analysis of the filtrate from the photoreduction reaction with APTS-Br-PBI@SiO_2 (100 TEOS) in the absence of light. On top, the gas chromatogram is shown and the main peak from iodobenzaldehyde is observed at 8.069 min. No peak assigned to benzaldehyde was found. The asterisks indicate impurities from solvent.

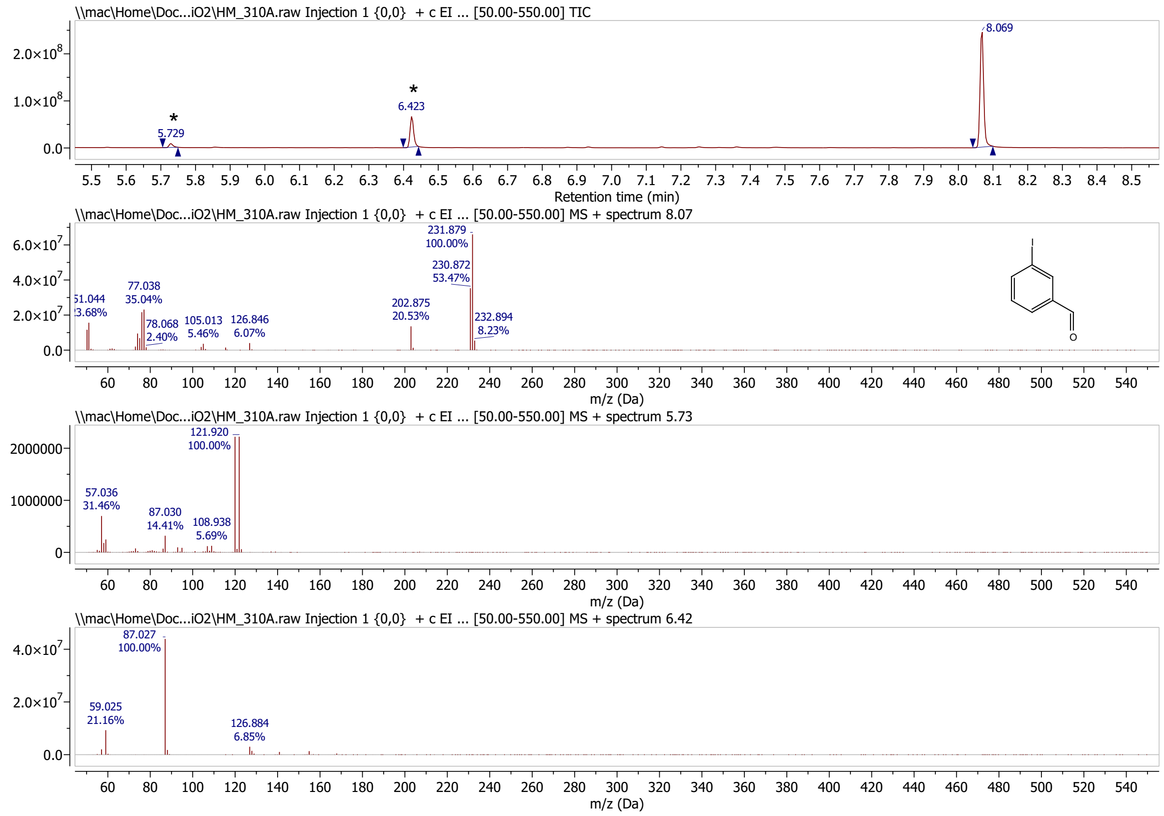

Figure S23. GC-MS analysis of the filtrate from the photoreduction reaction with SiO_2. On top, the gas chromatogram is shown and the main peak from iodobenzaldehyde is observed at 8.069 min. No peak assigned to benzaldehyde was found. The asterisks indicate impurities from solvent.

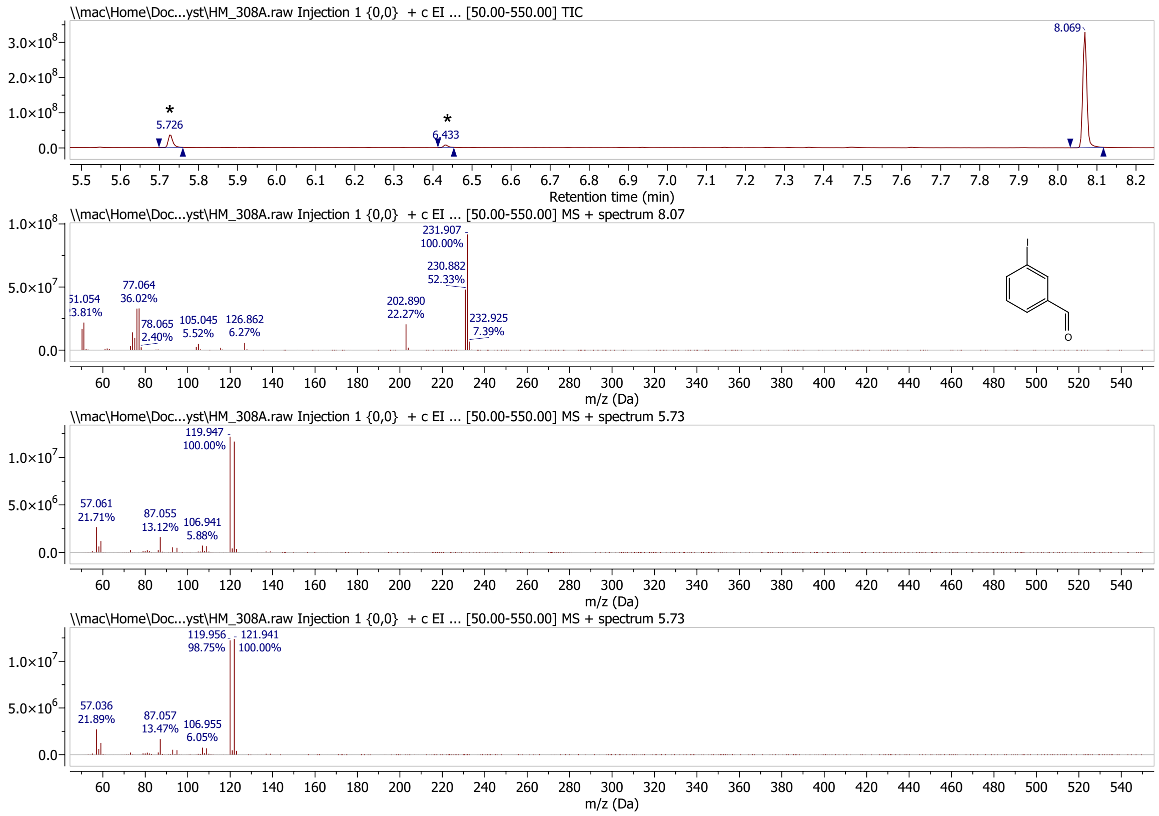

Figure S24. GC-MS analysis of the filtrate from the photoreduction reaction without catalyst. On top, the gas chromatogram is shown and the main peak from iodobenzaldehyde is observed at 8.069 min. No peak assigned to benzaldehyde was found. The asterisks indicate impurities from solvent.

3.5.3 Calibration curves of benzaldehyde and iodobenzaldehyde obtained from GC-MS

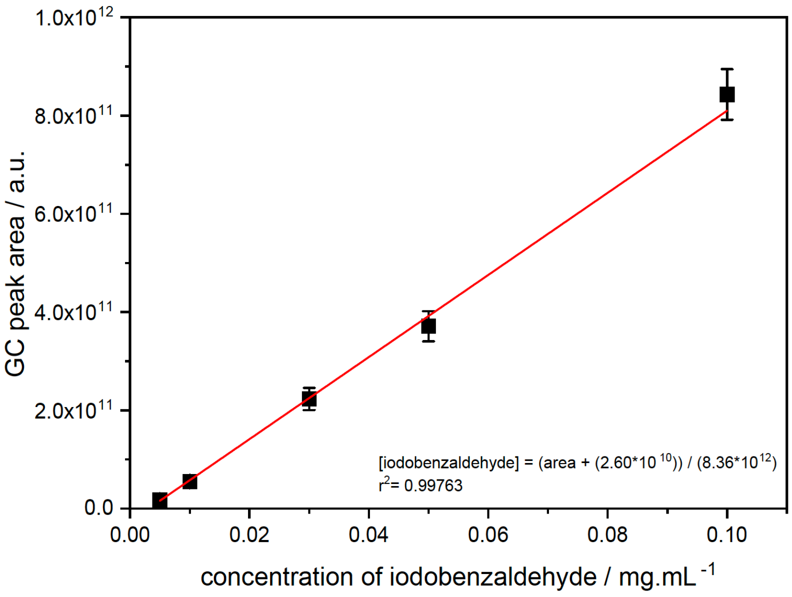

Figure S25. Calibration curve. Concentration of iodobenzaldehyde *versus* peak area from GC-MS.

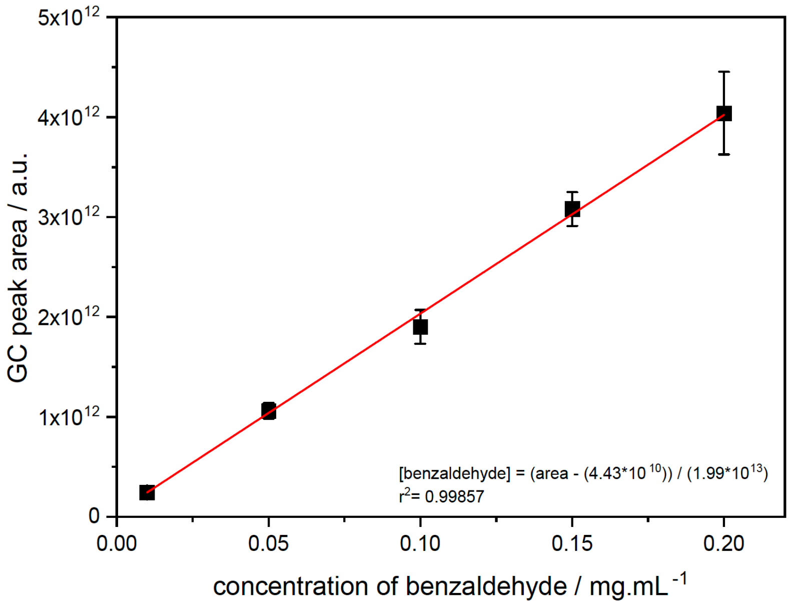

Figure S26. Calibration curve. Concentration of benzaldehyde *versus* peak area from GC-MS.

3.5.3.1 GC-MS spectra of the solutions used for the calibration curve

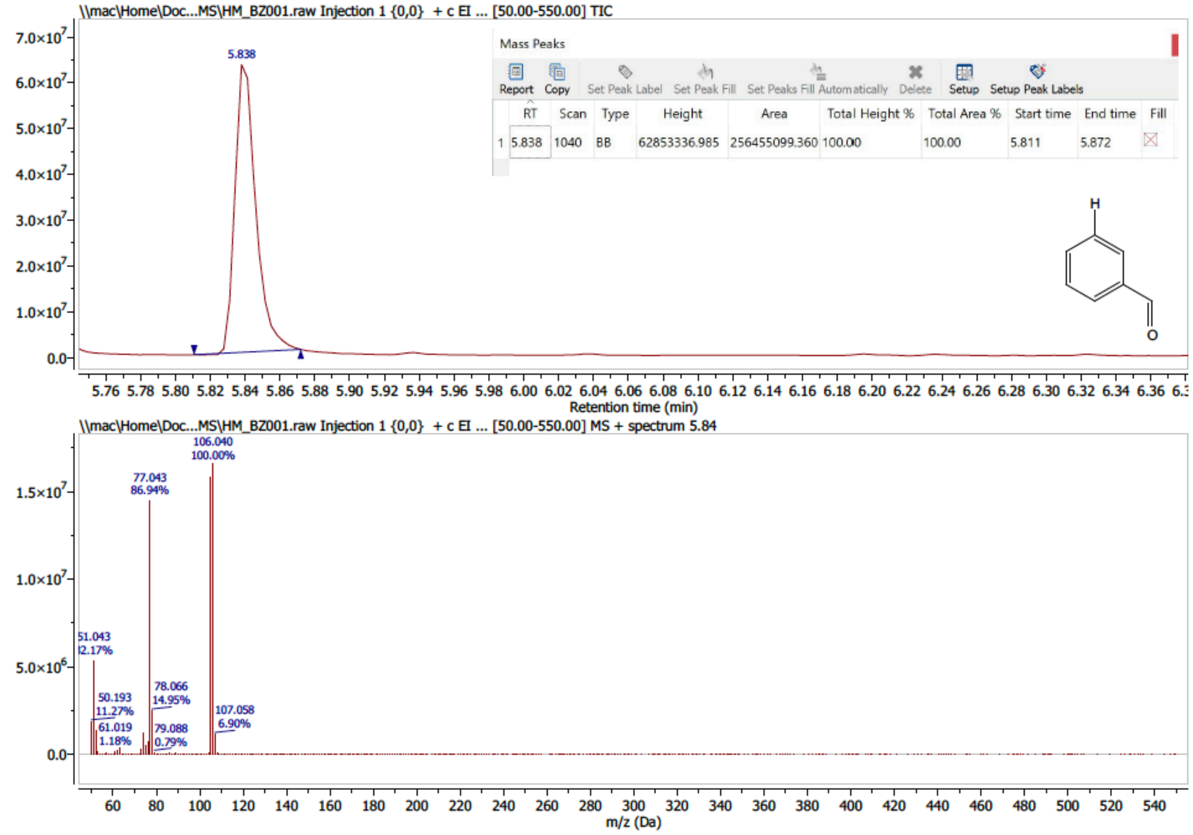

Figure S27. GC-MS spectra of benzaldehyde at 0.01 mg/mL.

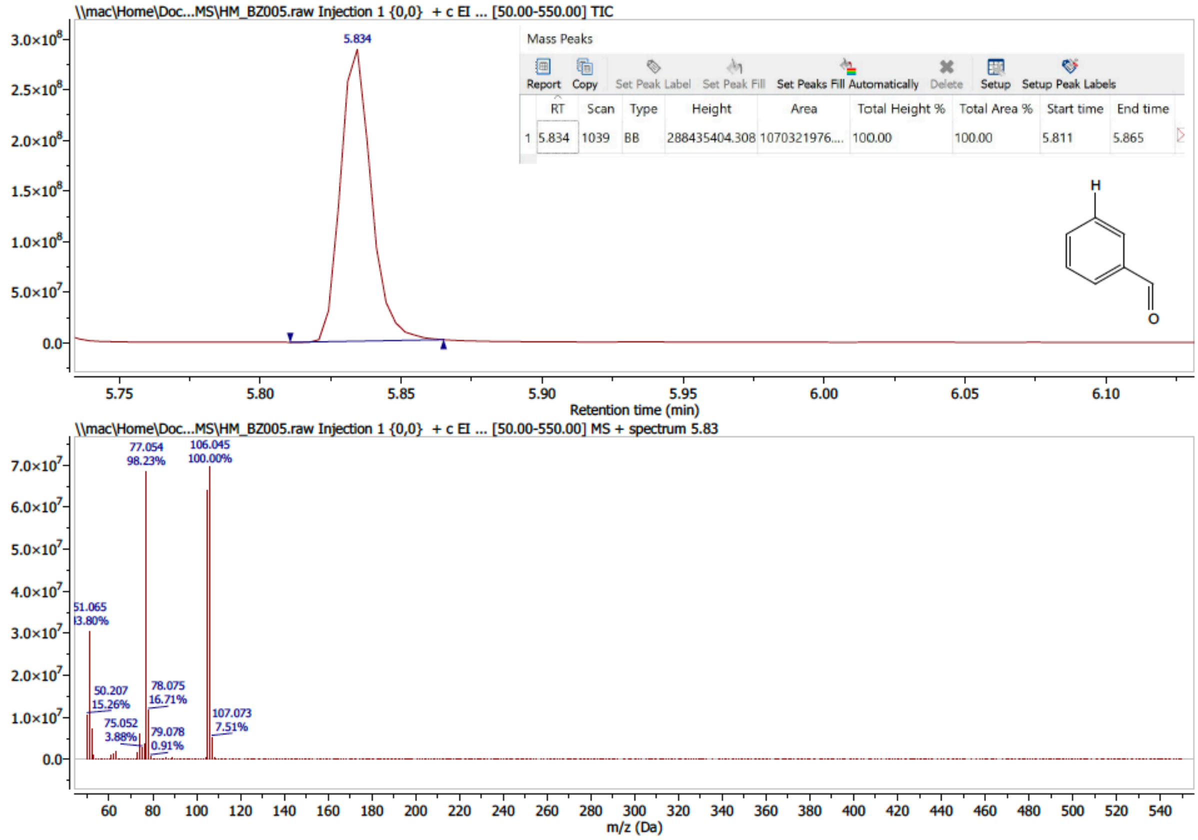

Figure S28. GC-MS spectra of benzaldehyde at 0.05 mg/mL.

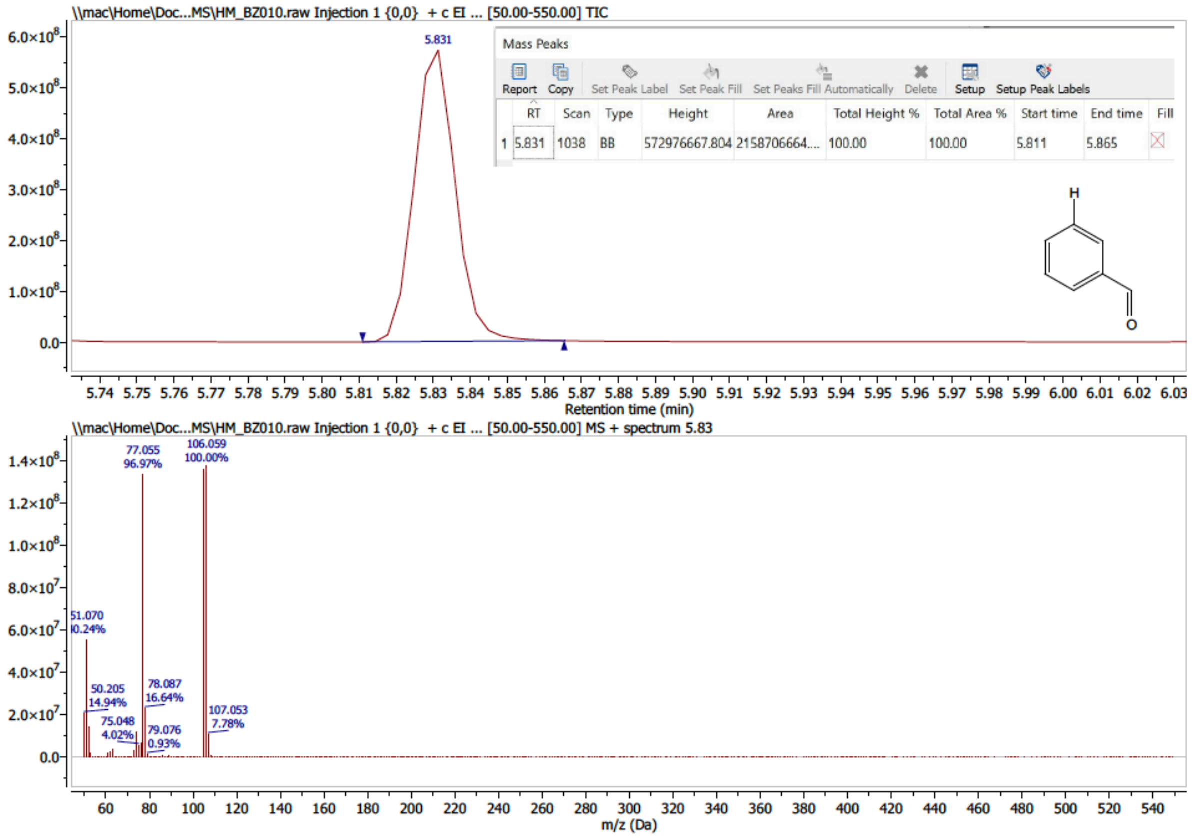

Figure S29. GC-MS spectra of benzaldehyde at 0.10 mg/mL.

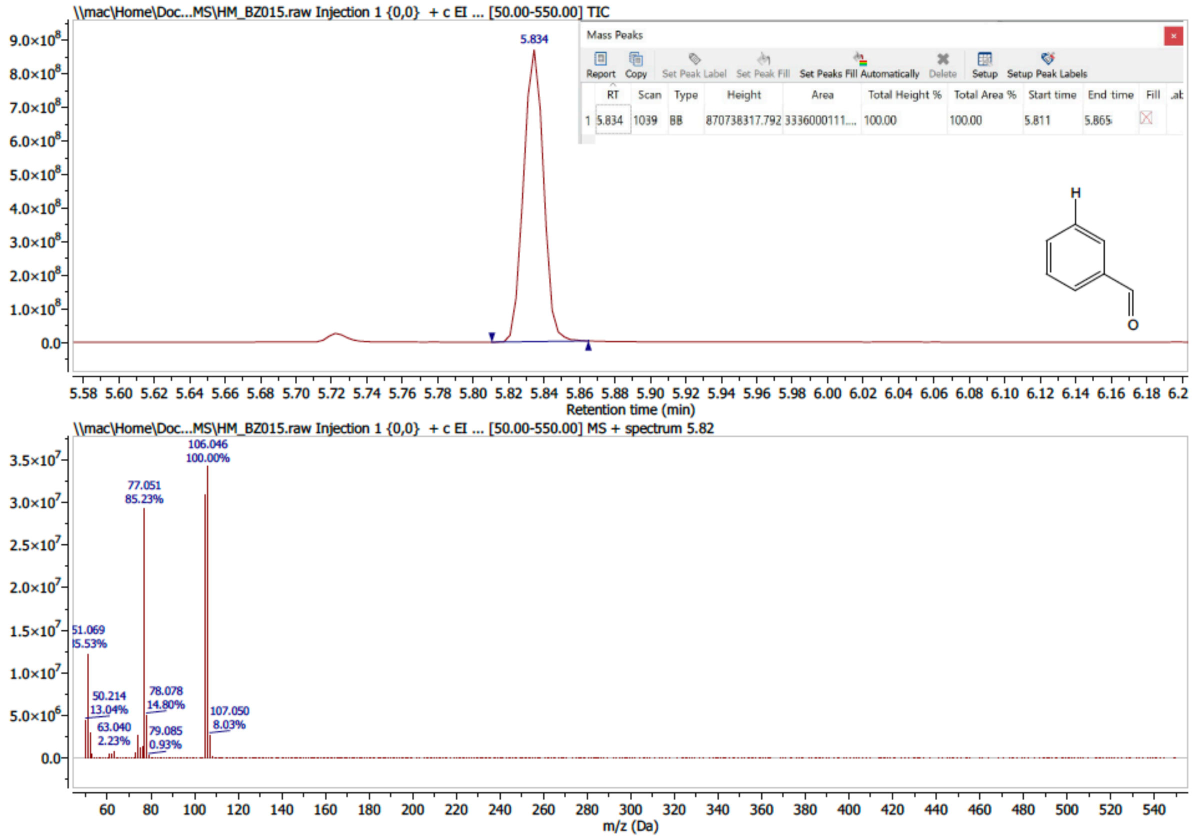

Figure S30. GC-MS spectra of benzaldehyde at 0.15 mg/mL.

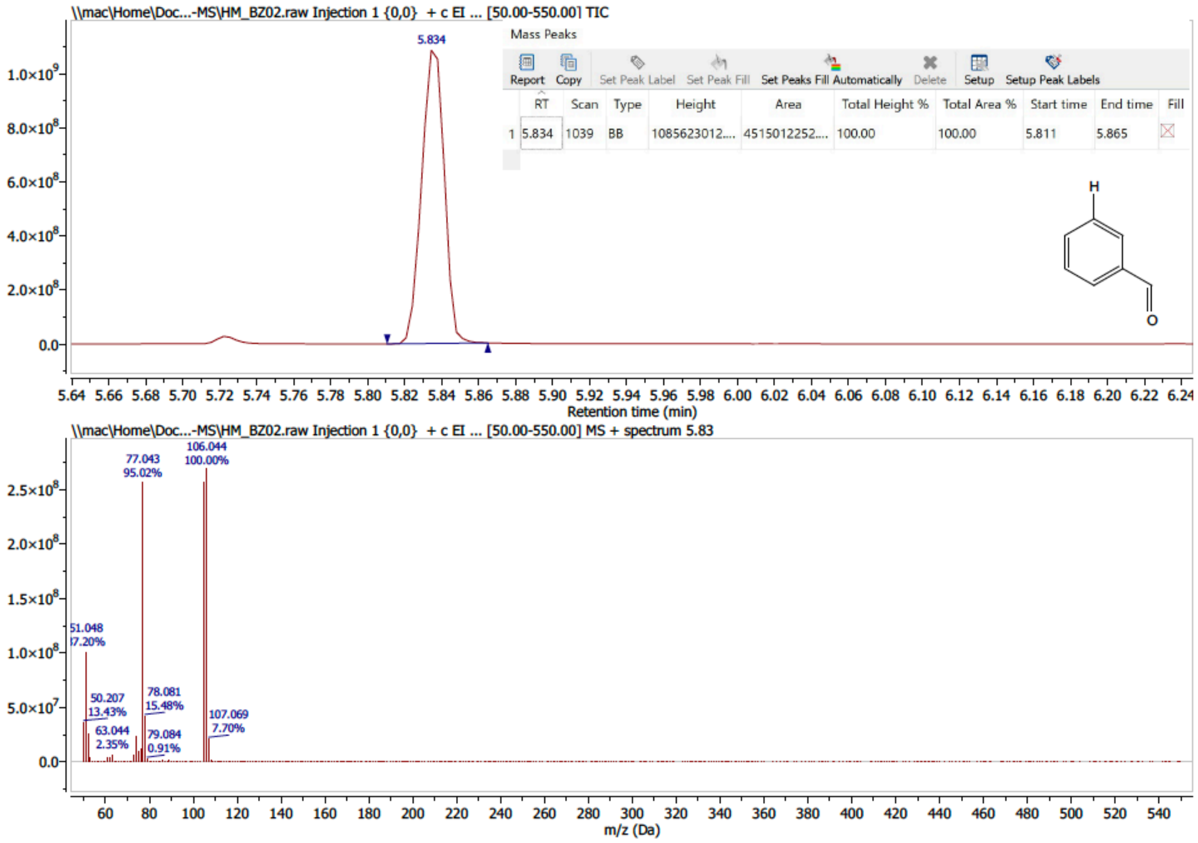

Figure S31. GC-MS spectra of benzaldehyde at 0.20 mg/mL.

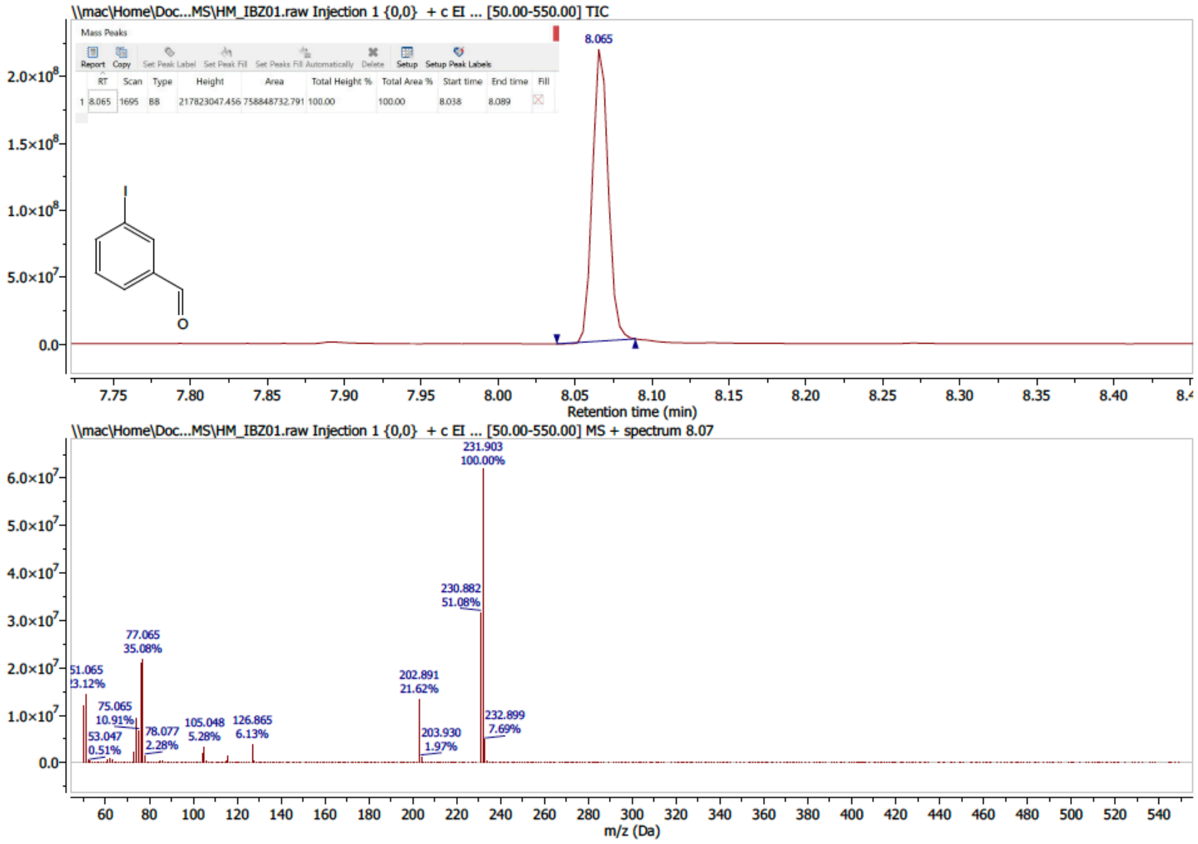

Figure S32. GC-MS spectra of iodobenzaldehyde at 0.10 mg/mL.

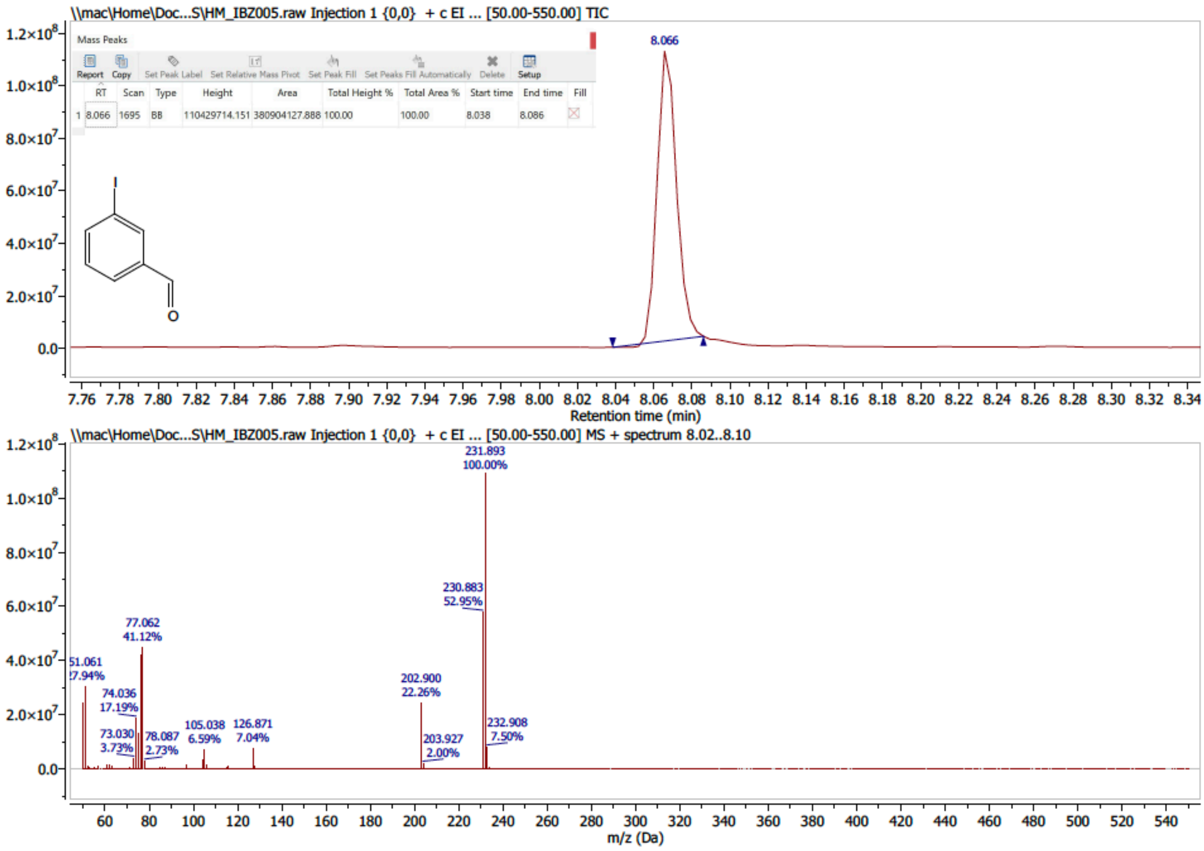

Figure S33. GC-MS spectra of iodobenzaldehyde at 0.05 mg/mL.

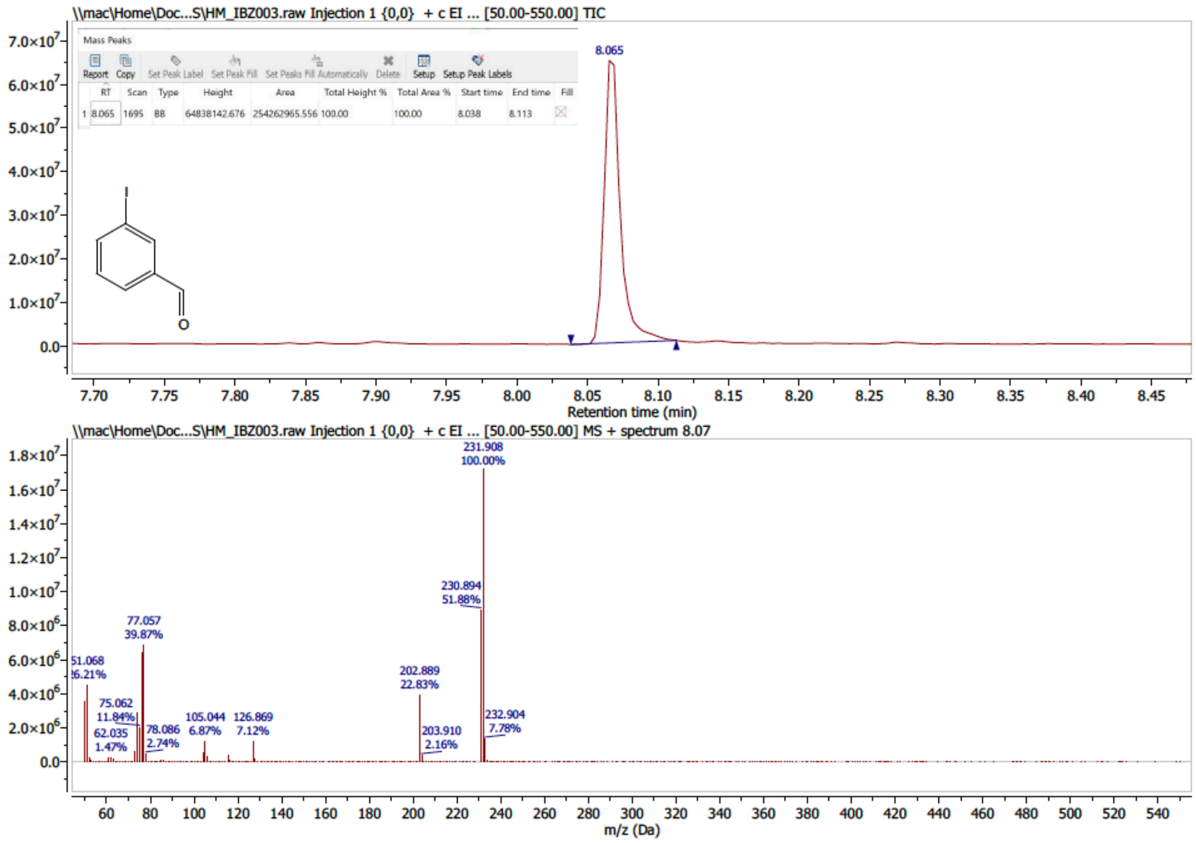

Figure S34. GC-MS spectra of iodobenzaldehyde at 0.03 mg/mL.

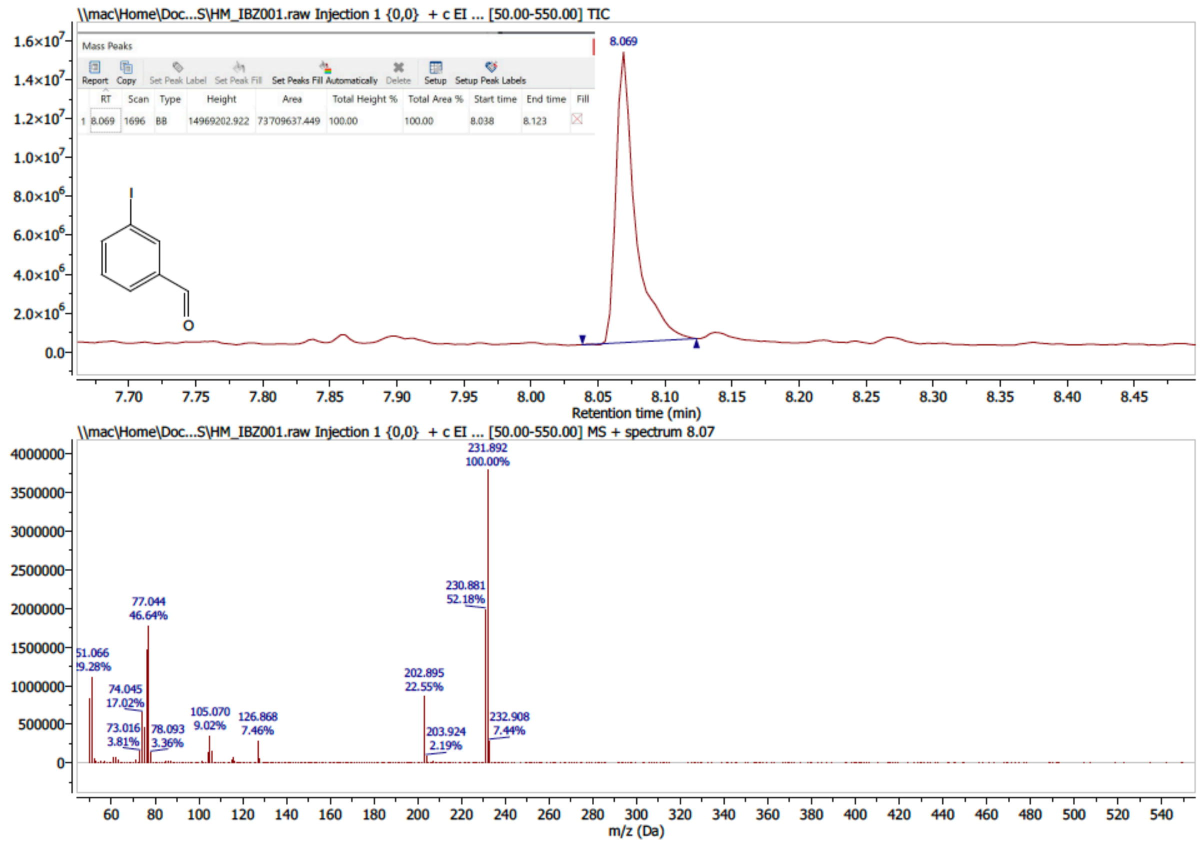

Figure S35. GC-MS spectra of iodobenzaldehyde at 0.01 mg/mL.

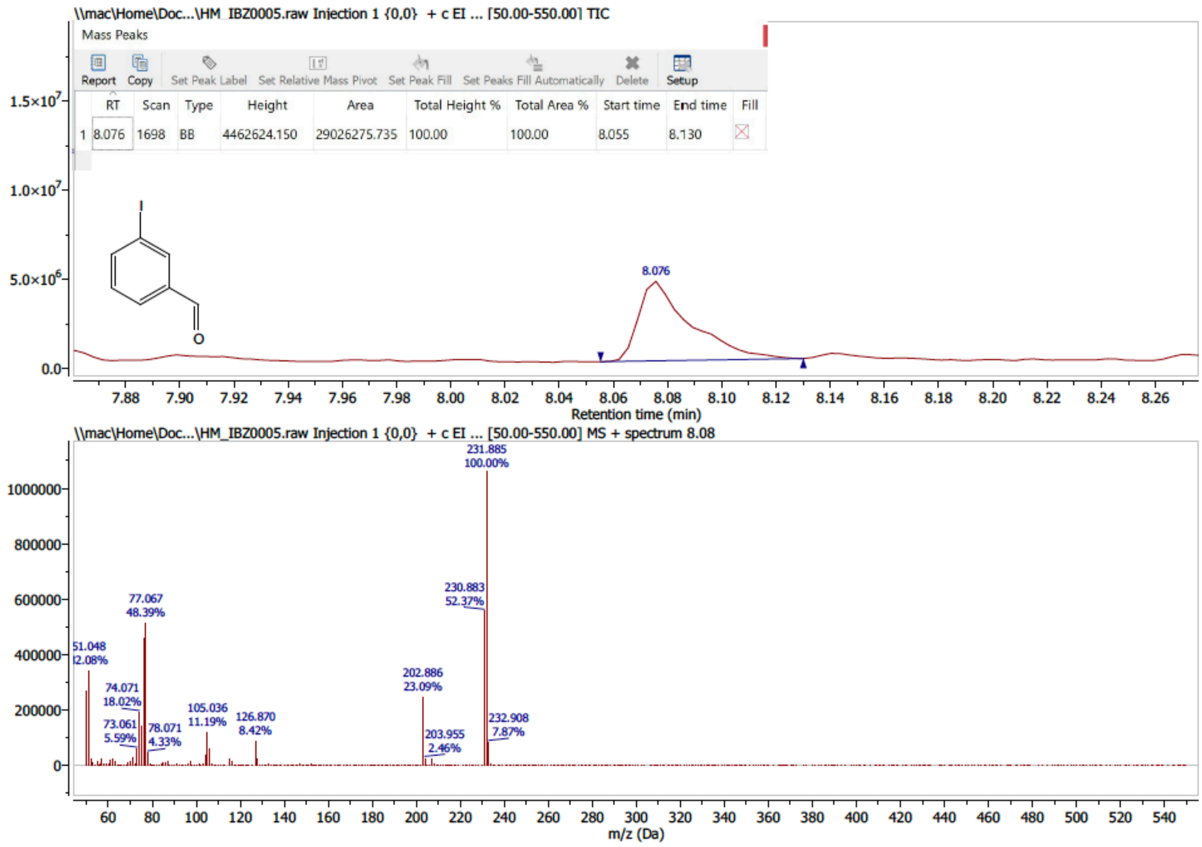

Figure S36. GC-MS spectra of iodobenzaldehyde at 0.005 mg/mL.

4. References

1 D. B. O'brien, T. C. Anglin and A. M. Massari, *Langmuir*, 2011, **27**, 13940–13949.
2 A. Scalabre, A. M. Gutiérrez-Vílchez, Á. Sastre-Santos, F. Fernández-Lázaro, D. M. Bassani and R. Oda, *J. Phys. Chem. C*, 2020, **124**, 23839–23843.
3 L. Gigli, R. Arletti, G. Tabacchi, M. Fabbiani, J. G. Vitillo, G. Martra, A. Devaux, I. Miletto, S. Quartieri, G. Calzaferri and E. Fois, *J. Phys. Chem. C*, 2018, **122**, 3401–3418.
4 M. Busby, A. Devaux, C. Blum, V. Subramaniam, G. Calzaferri and L. De Cola, *J. Phys. Chem. C*, 2011, **115**, 5974–5988.
5 A. Devaux, G. Calzaferri, I. Miletto, P. Cao, P. Belser, D. Brühwiler, O. Khorev, R. Häner and A. Kunzmann, *J. Phys. Chem. C*, 2013, **117**, 23034–23047.
6 A. Devaux, G. Calzaferri, P. Belser, P. Cao, D. Brühwiler and A. Kunzmann, *Chem. Mater.*, 2014, **26**, 6878–6885.
7 R. Kumarasinghe, T. Ito and D. A. Higgins, *Anal. Chem.*, 2020, **92**, 1416–1423.
8 X. Zhan, K. Cui, M. Dou, S. Jin, X. Yang and H. Guan, *RSC Adv.*, 2014, **4**, 47081–47086.
9 F. D. J. Trindade, E. R. Triboni, B. Castanheira and S. Brochsztain, *J. Phys. Chem. C*, 2015, **119**, 26989–26998.
10 P. Cao, O. Khorev, A. Devaux, L. Sägesser, A. Kunzmann, A. Ecker, R. Häner, D. Brühwiler, G. Calzaferri and P. Belser, *Chem. - A Eur. J.*, 2016, **22**, 4046–4060.
11 G. Qian, Y. Yang, Z. Wang, C. Yang, Z. Yang and M. Wang, *Chem. Phys. Lett.*, 2003, **368**, 555–560.
12 S. Liang, S. Liang, Y. Wang, Y. Wang, L. Mu, G. She, W. Shi and W. Shi, *Nanotechnology*, 2020, **31**, 7pp.
13 F. Corsini, E. Tatsi, A. Colombo, C. Dragonetti, C. Botta, S. Turri and G. Griffini, *Nano Energy*, 2021, **80**, 105551.
14 M. R. Böhmer and T. A. P. M. Keursten, *J. Sol-Gel Sci. Technol.*, 2000, **19**, 361–364.
15 D. Clarke, S. Mathew, J. Matisons, G. Simon and B. W. Skelton, *Dye. Pigment.*, 2012, **92**, 659–667.
16 E. Lucenti, C. Botta, E. Cariati, S. Righetto, M. Scarpellini, E. Tordin and R. Ugo, *Dye. Pigment.*, 2013, **96**, 748–755.
17 Y. Shao, X. Zhang, K. Liang, J. Wang, Y. Lin, S. Yang, W. Bin Zhang, M. Zhu and B. Sun, *RSC Adv.*, 2017, **7**, 16155–16162.
18 J. Li, J. Wang, J. Sun, S. Bai and X. Wu, *J. Lumin.*, 2019, **206**, 547–553.
19 F. J. Trindade, G. J. T. Fernandes, A. S. Araújo, V. J. Fernandes, B. P. G. Silva, R. Y. Nagayasu, M. J. Politi, F. L. Castro and S. Brochsztain, *Microporous Mesoporous Mater.*, 2008, **113**, 463–471.
20 H. Li, Y. Zhang, B. Chen, Y. Wang, C. Teh, G. H. B. Ng, J. Meng, Z. Huang, W. Dong, M. Y. Tan, X. Sun, X. Sun, X. Li and J. Li, *ACS Appl. Bio Mater.*, 2019, **2**, 1569–1577.
21 B. González-Santiago, O. Medina-Juárez, C. A. Benitez-Delgado, F. Rojas-González, E. Salas-Bañales and M. Á. Garcia-Sanchez, *J. Lumin.*, 2019, **210**, 285–292.
22 M. Schneider and K. Müllen, *Chem. Mater.*, 2000, **12**, 352–362.
23 C. Xing, G. Yu, T. Chen, S. Liu, Q. Sun, Q. Liu, Y. Hu, H. Liu and X. Li, *Appl. Catal. B Environ.*, 2021, **298**, 120534.
24 X. Ning, J. Chittigori, Y. Li, G. Horner, Z. Zhou, C. K. Ullal and L. Schadler, *J. Colloid Interface Sci.*, 2019, **556**, 753–760.
25 H. Wang, K. Schaefer, A. Pich and M. Moeller, *Chem. Mater.*, 2011, **23**, 4748–4755.
26 T. Ribeiro, S. Raja, A. S. Rodrigues, F. Fernandes, J. P. S. Farinha and C. Baleizão, *RSC Adv.*, 2013, **3**, 9171–9174.
27 D. Sriramulu, E. L. Reed, M. Annamalai, T. V. Venkatesan and S. Valiyaveettil, *Sci. Rep.*, 2016, **6**, 1–10.
28 D. Sriramulu, S. P. Turaga, A. A. Bettiol and S. Valiyaveettil, *RSC Adv.*, 2017, **7**, 32692–32702.
29 D. Sriramulu, S. P. Turaga and A. A. Bettiol, *Sci. Rep.*, 2017, **7**, 1–11.
30 J. Shang, H. Tang, H. Ji, W. Ma, C. Chen and J. Zhao, *Cuihua Xuebao/Chinese J. Catal.*, 2017, **38**, 2094–2101.
31 H. Langhals, A. J. Esterbauer and S. Kinzel, *New J. Chem.*, 2009, **33**, 1829–1832.
32 N. Mizoshita, T. Tani and S. Inagaki, *Adv. Funct. Mater.*, 2011, **21**, 3291–3296.
33 A. Cardelli, L. Ricci, G. Ruggeri, S. Borsacchi and M. Geppi, *Eur. Polym. J.*, 2011, **47**, 1589–1600.
34 J. Gorman, R. Pandya, J. R. Allardice, M. B. Price, T. W. Schmidt, R. H. Friend, A. Rao and N. J. L. K. Davis, *J. Phys. Chem. C*, 2019, **123**, 3433–3440.
35 Y. M. Jeon, T. H. Lim, C. W. Lee, J. W. Cheon and M. S. Gong, *J. Ind. Eng. Chem.*, 2007, **13**, 518–522.
36 J. Blechinger, R. Herrmann, D. Kiener, F. J. García-García, C. Scheu, A. Reller and C. Bräuchle, *Small*, 2010, **6**, 2427–2435.

37 Z. Gao, W. Huang, Y. Zheng, Y. Lu, S. You, J. Shen and M. Yin, *RSC Adv.*, 2016, **6**, 46226–46230.
38 Y. Luo and J. Lin, *J. Colloid Interface Sci.*, 2006, **297**, 625–630.
39 V. V. Semenov, N. V. Zolotareva, E. Y. Ladilina, S. A. Lermontova, L. G. Klapshina, I. S. Grigor'ev, M. A. Lopatin, A. I. Kirillov, T. I. Kulikova and G. A. Domrachev, *Russ. J. Gen. Chem.*, 2011, **81**, 1496–1506.
40 R. Herrmann, M. Rennhak and A. Reller, *Beilstein J. Nanotechnol.*, 2014, **5**, 2413–2423.
41 A. Marraccini, F. M. Carlini, G. Bottaccio, A. Pasquale and G. Maranzana, *EP 0 140 688 A2*, 1985, 1–32.
42 N. Mizoshita, T. Tani and S. Inagaki, *Chem. Commun.*, 2012, **48**, 10772–10774.
43 M. A. Wahab, H. Hussain and C. He, *Langmuir*, 2009, **25**, 4743–4750.
44 M. S. Kim and J. Y. Chang, *J. Mater. Chem.*, 2011, **21**, 8766–8771.
45 T. Ribeiro, E. Coutinho, A. S. Rodrigues, C. Baleizão and J. P. S. Farinha, *Nanoscale*, 2017, **9**, 13485–13494.
46 F. L. de Castro, J. G. Santos, G. J. Turolla Fernandes, A. S. de Araújo, V. J. Fernandes, M. J. Politi and S. Brochsztain, *Microporous Mesoporous Mater.*, 2007, **102**, 258–264.
47 N. Mizoshita, T. Tani, H. Shinokubo and S. Inagaki, *Angew. Commun.*, 2012, **51**, 1156–1160.
48 A. S. Rodrigues, T. Ribeiro, F. Fernandes, J. P. S. Farinha and C. Baleizão, *Microsc. Microanal.*, 2013, **19**, 1216–1221.
49 A. I. Carrillo, A. Elhage, M. L. Marin and A. E. Lanterna, *Chem. - A Eur. J.*, 2019, **25**, 14928–14934.
50 Z. Yang, W. Fan, J. Zou, W. Tang, L. Li, L. He, Z. Shen, Z. Wang, O. Jacobson, M. A. Aronova, P. Rong, J. Song, W. Wang and X. Chen, *J. Am. Chem. Soc.*, 2019, **141**, 14687–14698.
51 I. Meazzini, N. Willis-Fox, C. Blayo, J. Arlt, S. Clément and R. C. Evans, *J. Mater. Chem. C*, 2016, **4**, 4049–4059.
52 D. Zhang, J. Liu, S. Y. Gao, M. V. Bermeshev, Z. Chen and X. K. Ren, *J. Mater. Chem. C*, 2021, **9**, 9236–9241.
53 Z. Chen, H. N. Dinh and E. Miller, Eds., *Photoelectrochemical Water Splitting: Standards, Experimental Methods and Protocols*, Springer, 2018.
